# Shotgun metagenomics of honey DNA: Evaluation of a methodological approach to describe a multi-kingdom honey bee derived environmental DNA signature

**DOI:** 10.1371/journal.pone.0205575

**Published:** 2018-10-31

**Authors:** Samuele Bovo, Anisa Ribani, Valerio Joe Utzeri, Giuseppina Schiavo, Francesca Bertolini, Luca Fontanesi

**Affiliations:** 1 Department of Agricultural and Food Sciences, University of Bologna, Bologna, Italy; 2 Department of Bio and Health Informatics, Technical University of Denmark, Kongens Lyngby, Denmark; University of Hyogo, JAPAN

## Abstract

Honey bees are considered large-scale monitoring tools due to their environmental exploration and foraging activities. Traces of these activities can be recovered in the honey that also may reflect the hive ecological micro-conditions in which it has been produced. This study applied a next generation sequencing platform (Ion Torrent) for shotgun metagenomic analysis of honey environmental DNA (eDNA). The study tested a methodological framework to interpret DNA sequence information useful to describe the complex ecosystems of the honey bee colony superorganism, its pathosphere and the heterogeneity of the agroecological environments and environmental sources that left DNA marks in the honey. Analysis of two honeys reported sequence reads from five main organism groups (kingdoms or phyla): arthropods (that mainly included reads from *Apis mellifera*, several other members of the Hymenotpera, in addition to members of the Diptera, Coleoptera and Lepidoptera, as well as aphids and mites), plants (that clearly confirmed the botanical origin of the two honeys, i.e. orange tree blossom and eucalyptus tree blossom honeys), fungi and bacteria (including common hive and honey bee gut microorganisms, honey bee pathogens and plant pathogens), and viruses (which accounted for the largest number of reads in both honeys, mainly assigned to *Apis mellifera* filamentous virus). The shotgun metagenomic approach that was used in this study can be applied in large scale experiments that might have multiple objectives according to the multi-kingdom derived eDNA that is contained in the honey.

## Introduction

Biomonitoring methods traditionally rely on the study and sampling of the organisms under investigation that are first precisely identified and characterized. Environmental monitoring for ecological studies also rely on an appropriately designed sampling strategy to cover the area under investigation [1, 2]. These approaches face several challenges including the high logistic costs of specimen and sample collection (in case many data points are needed), the difficulties in reaching remote environments and in obtaining unbiased and complete inventories of the organisms or materials of the analyzed area. These limitations are in many cases overcome by the sequencing of environmental DNA (eDNA), defined as DNA extracted from environmental- or organismal-related matrices derived by cells or tissue fragments [3–6]. Compared to traditional approaches, eDNA has increased detection sensitivity and precision as it can provide information on organisms without their physical and temporal presence, including elusive species, using recovered information from their DNA traces left in the environment [7–9]. The introduction of next generation sequencing technologies in eDNA analyses have revolutionized this area of investigation facilitating the direct use of DNA information for many different purposes [10–12].

Honey bees are considered large-scale monitoring tools due to their environmental exploration and foraging activities. Analysis of their hive products, i.e. pollen and honey bee derived products (wax, propolis and honey), has been used to detect the level of environmental contaminants and pollutants and to identify their origin [13, 14]. These materials can be also considered interesting sources of eDNA. In particular, honey reflects honey bee activities and honey contained eDNA (i.e. derived from pollen) has been used to evaluate honey bee foraging behavior and the botanical composition for authentication [15–18]. Information from plant-sucking insects producing honeydew can be recovered in honey as honey bees routinely collect this sweet material in which the producer hemipters leave their DNA traces [19]. Other DNA traces left in the honey have been used to identify the bee species and the honey bee subspecies that produced it [17, 20–22]. Amplified fungi and bacterial DNA from honey has been used for forensic and food safety analyses [23, 24]. Honey extracted DNA can be also useful to recover information from honey bee pathogens. For example, *Nosema ceranae* DNA has been detected in honey, which has been also suggested as one of the potential sources of introduction of this microsporidian pathogen in Australia [25]. Other authors proposed the use of polymerase chain reaction (PCR) methods to identify the presence of *Paenibacillus larvae* (causing American foulbrood) spores in naturally infected honey samples [26, 27]. In general, analysis of honey DNA has been reported using i) specific PCR based assays, ii) or using universal primers for barcoding or iii) metabarcoding approaches, when coupled with next generation sequencing, to resolve the taxonomic composition of complex honey eDNA sources [15, 17, 18]. All these methods rely on PCR amplification of targeted DNA regions with inherent limitations due to the design of the assays. For example, the PCR step could unevenly amplify the targeted DNA fragment across all targeted organisms, or some taxa may not be amplified at all, leading to biased biodiversity assessments [3, 4]. Moreover, only by using multiple primer sets (targeting different organelle or genome DNA regions) could it be possible to cover all organisms that might be represented in the honey.

Shotgun metagenomics sequencing, also known as environmental genomics, community genomics or ecogenomics, is based on direct sequencing of eDNA samples without any PCR enrichment [4, 28]. For this reason, it has been considered as an unbiased approach able to describe the complexity of environmental samples that can contain hundreds or even thousands of distinct species belonging to different kingdoms or phyla, that would be difficult to capture or characterize using standard barcoding or metabarcoding approaches [29, 30]. For example, shotgun metagenomic sequencing has been used to identify new microorganism species and reconstruct their genome [31]. The interpretation of taxonomic results is quite challenging because sequencing data are spread all over genome regions from many different organisms and not only on standard DNA gene regions commonly used for taxonomic assignments. Thus, the taxonomic interpretation would be biased towards the organisms for which their genomes are completely sequenced and available in databases. Bioinformatic analyses of these data are also quite challenging as they should overcome: i) the problem derived by the availability of many genomes useful for the comparison, ii) the problem derived by the sequencing depth needed to saturate sequence information of an eDNA with unknown composition and, in turn, iii) the computation problems that eDNA analyses may generate [30].

In this study, we used a next generation semiconductor-based sequencing platform (i.e. Ion Torrent Personal Genome Machine) for shotgun metagenomic sequencing of honey DNA. We tested a methodological framework to interpret eDNA data with a low sequencing depth (needed to reduce sequencing cost) and obtain DNA derived information useful to describe the complex ecosystems of the honey bee colony superorganism, its pathosphere and other agroecological recovered DNA sources. Even using a sparse shotgun metagenomic sequencing approach, we could confirm the potential usefulness of honey bees for biomonitoring environmental areas covered by the colony with possible agroecological cost-effective applications derived by a multi-kingdom DNA signature.

## Materials and methods

### Honey samples and DNA extraction

Two honey samples were provided by beekeepers. One was an orange tree blossom honey produced in Caltanisetta province (Sicily, Italy) in the year 2014. The other one was a eucalyptus tree blossom honey produced in Messina province (Sicily, Italy) in the year 2015. The selections of these two honeys was based by the fact that it could be potentially possible to evaluate their botanical prevalence using a metagenomic approach considering the fact that the genomes of the orange tree (*Citrus sinensis*) and eucalyptus tree (*Eucalyptus grandis*) have been already sequenced [32, 33]. In addition, botanical characterization of these two honeys has been also already obtained by Utzeri et al. [18] using a metabarcoding approach.

DNA was extracted as previously described [18, 22]. Briefly, from each honey sample, a total of 50 g of honey was divided in four aliquots and used for DNA extraction. After a few washing and centrifugation steps [18, 22], the obtained pellet was resuspended in 5 mL of ultrapure water and the content of the four tubes was then merged in a single Falcon tube and further diluted with ultrapure water to reach 45 mL. These two tubes were again centrifuged at 5000 *g* for 25 min at room temperature. The resulting supernatant was discarded and the obtained pellet was resuspended in 0.5 mL of ultrapure water, transferred into a 1.5 mL tube containing 10 glass beads (500 μm) and vortexed for 3 minutes. The liquid was transferred in a new 1.5 mL tube and used for the subsequent DNA extraction steps that was carried out adding one mL of CTAB extraction buffer [2% (w/v) cetyltrimethylammoniumbromide; 1.4 M NaCl; 100 mM Tris-HCl; 20 mM EDTA; pH 8], prepared with 5 μL of RNase A solution (10 mg/mL) and 30 μL of proteinase K (20 mg/mL). The two tubes were then incubated at 65° C for 90 min with gentle mixing. After, they were cooled at room temperature and centrifuged for 10 min at 16,000 *g*. About 700 μL of supernatant from each tube was transferred in another tube containing 500 μL of chloroform/isoamyl alcohol (24:1), vortexed for 30 s and then centrifuged at 16,000 *g* for 15 min at room temperature. The supernatant was transferred in a new 1.5 mL tube and the DNA was precipitated first with isopropanol and then with ethanol/water 70:30 v/v. The DNA pellets were rehydrated with 30 μL of ultrapure H_2_O and stored at -20°C till their use. Extracted DNA was electrophoresed on TBE1X 1% agarose gels and stained with 1X GelRed Nucleic Acid Gel Stain (Biotium Inc., Hayward, CA, USA). This quality control analysis evidenced that DNA from both honeys was degraded, as expected, confirming previous evaluations [22].

### Ion Torrent sequencing

Two Ion Torrent libraries (one for each honey) were prepared following a standard protocol already applied in other experiments (i.e. [34, 35]). In summary, about 200 ng of DNA from each extracted sample was enzymatically sheared, end-repaired and adapter ligated using the Ion Xpress Plus Fragment Library kit (Life Technologies). Then, resulting DNA material was size selected using the e-gel system (Invitrogen, Carlsbad, CA, USA) and bands corresponding to 200 bp of inserts were collected and quantified by qPCR using a StepOnePlus Real-Time PCR System (Life Technologies). Then, DNA was end-repaired and ligated with a specific barcode using the Ion Xpress Plus Fragment Library and Ion Xpress Barcode Adapters 1–16 kits (Thermo Fisher Scientific Inc.). Then, the two libraries were quantified with the Ion Library Quantitation kit (Thermo Fisher Scientific Inc.) by qPCR with the StepOnePlus Real-Time PCR System (Thermo Fisher Scientific Inc.), pooled together and clonally amplified by emulsion PCR with the Ion PGM Hi-Q OT2 kit. Sequencing reactions were obtained on an Ion 318 v2 chip (Thermo Fisher Scientific Inc.) using the Ion PGM Hi-Q Sequencing kit. The same chip was used to sequence at the same time other barcoded libraries used for different purposes.

### Data processing and analyses

Sequence reads were automatically processed by the Ion Torrent Suite v.4.6 on the Ion Torrent Server (Thermo Fisher Scientific Inc.) which (i) eliminated polyclonal sequences and sequences of low quality (Q<10) and (ii) trimmed adapters and low quality 3′-ends. Reads were then separated according to their barcode. Reads obtained from the two analysed honeys were subsequently quality checked with FastQC v.0.11.5 (available at https://www.bioinformatics.babraham.ac.uk/projects/fastqc/). Data inspection highlighted the necessity of an additional processing step to obtain high quality data. Thus, reads were further filtered by using PRINSEQ Lite v.0.20.4 [36] as follows: i) trimming of the 3′-end up to reaching a length of 270 bp, ii) trimming of the 5′- and 3′- ends for poly-A/T sequences > 5, iii) trimming of the 5′- and 3′- ends up to reaching a base with a Q > 20, and iv) exclusion of reads with an average Q < 20.

The National Center for Biotechnology Information (NCBI) nt database (comprising a total of 43,798,648 DNA sequences, download from ftp://ftp.ncbi.nlm.nih.gov/blast/db/FASTA/nt.gz; August, 2017 [37]) was used as annotating resource. BLAST+ v.2.6.0 (ftp://ftp.ncbi.nlm.nih.gov/blast/executables/blast+/2.2.30/) was used to align (command *blastn*, default parameters) reads on the NCBI nt database, previously indexed (command *makeblastdb*), taking into account the NCBI taxonomy identifier (*taxid*). For each read, we retained all the alignments having, at the same time, a sequence coverage, a sequence identity and an E-value equal to the top alignment (i.e. the one with the lowest E-value).

The taxonomic assignment followed the Lowest Common Ancestor (LCA) approach. Taxa information (ID, scientific name, taxonomic level) and relationships were downloaded from the NCBI taxonomy resource ([37]; file ftp://ftp.ncbi.nlm.nih.gov/pub/taxonomy/taxdump.tar.gz; January, 2018). Based on the BLAST statistics, we retained only reads satisfying the following parameters: a sequence length ≥ 30, a coverage ≥ 75% and an E-value ≤ 0.01. The LCA procedure was implemented in Python 2.7 by using the graph library NetworkX v.2.0 (https://networkx.github.io/). Alignments with DNA sequences labelled with “*taxids*” belonging to the “environmental samples” class were not considered during the annotation procedure. Reads were further divided into five datasets representing different “organism groups” (kingdoms or phyla): plants (taxid:33090), arthropods (taxid:6656), fungi (taxid:4751), viruses (taxid:10239) and bacteria (taxid:2). Following Cowart et al. [38] who described a methodology to analyze metagenomic sequence data obtained from marine eDNA, exported datasets were converted to presence or absence of taxonomically assigned putative molecular operational taxonomic units (MOTU), a definition for groups when only DNA sequence information is available. The use of putative MOTU acknowledges the limits of a metagenomic eDNA analysis that has to deal with the incomplete reference database for taxonomic assignment and the limited sequencing depth that would not be able to capture all organisms present in the samples. The use of the presence/absence criterion to report the number of sequences assigned to a given MOTU does not necessarily reflect species abundance [38]. This might be due to various factors other than the abundance of individual organisms of a given species, including the genome size of the organisms of different kingdoms and taxa, the completeness of the genome sequence of the distinct organisms present in the reference database, the number of cells or residues left by the organisms in the honey and the DNA extraction procedure. For these reasons, we reported putative MOTUs using the taxa levels of species and genus. We also evaluated the effect of the sequence identity thresholds on taxa identification by counting the number of putative MOTUs (identified at the NCBI database with the term taxids) and reads as a function of the sequence identity itself (moving the threshold from 100% to 50%). For each honey and dataset, statistics were obtained for the genus and species levels, separately.

Species richness, (i.e. the total number of putative MOTUs) was estimated within each “organism group” (kingdom or phylum), by computing the richness estimator Chao1 [39], as implemented in EstimateS software v. 9.1.0 (http://viceroy.colorado.edu/estimates/index.html). The strict relationship between sequencing efforts and the coverage of the different organism groups was evaluated by means of rarefaction curves. For each honey, the average number of putative species or genera (for each organism group, separately) was plotted as a function of the percentage of sequenced reads, randomly sampled (without replacement). One hundred different sets of randomly sampled reads were used to compute the average number of these taxa (considering only those that were represented by at least two reads).

## Results

### Sequence reads and sequence assignment

Table 1 reports the number of reads that were obtained and then considered for this shotgun metagenomic analysis. Data processing steps filtered away a different number of raw reads from the two libraries (about 20% and 43% of reads from the libraries of the orange tree honey and eucalyptus tree honey, respectively), probably due to different degradation levels of the DNA isolated from the two samples. Anyway, from both libraries, a similar number of filtered reads remained (~270,000) to be considered for further analyses.

**Table 1:** Sequenced and aligned reads of the analyzed honeys.

Honey	Raw reads	Mapped reads	Retained reads^†^
Orange tree blossom honey	339,581	279,709	270,439
Eucalyptus tree blossom honey	467,951	286,484	264,903

^†^ Reads have been retained after pruning reads matching sequence entries labelled with taxids belonging to “environmental samples” class. A few other retained sequences (<1,000 per honey) were discarded later since they did not match the considered kingdoms or phyla (see Table A in S1 File).

Filtered reads were divided according to the mapping results (based on the first matched sequences in BLASTN analysis) into five datasets, representing five main honey “organism groups” (kingdoms or phyla) identified in this food matrix: arthropods (NCBI taxid: 6656), plants (taxid: 33090), fungi (taxid: 4751), bacteria (taxid: 2) and viruses (taxid: 10239).

**Fig 1:**
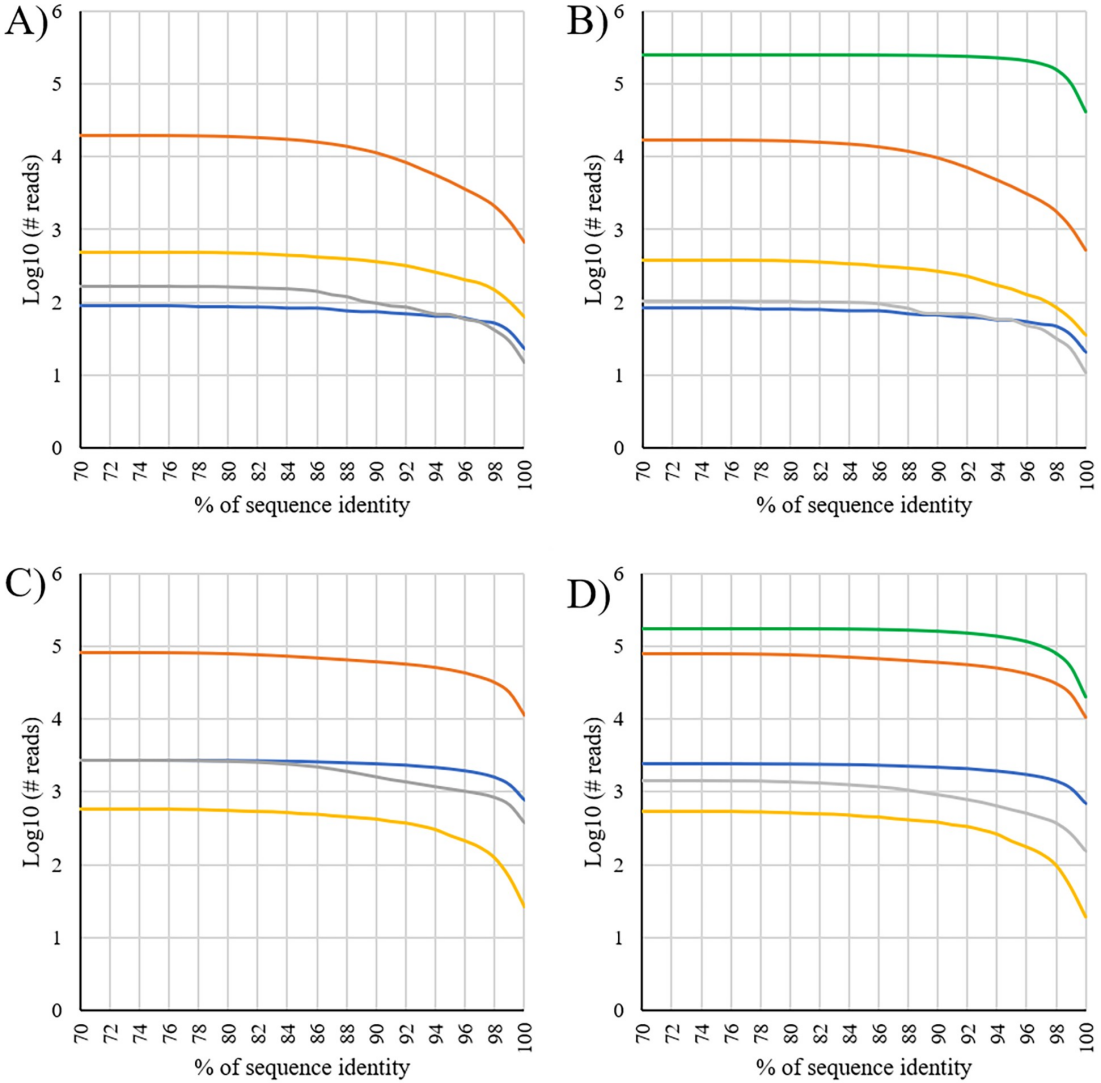
Effect of minimum sequence identity cut-off values on the number of mapped sequenced reads (considering the genus and species levels). A) Orange tree blossom honey, genus level; B) Orange tree blossom honey, species level; C) Eucalyptus tree blossom honey, genus level; D) Eucalyptus tree blossom honey, species level. Colors are as follow: blue, arthropods; yellow, plants; gray, fungi; orange, bacteria; green, viruses. Data are not reported for viruses at the genus level since this level is missing for some of them, leading to unreliable statistics. Plots are reported for a sequence identity ranging from 100 to 70% (from the left to the right) as lower values did not change the number of assigned sequences (as evidenced from the plateau reached at all levels).

Analyses evaluated the level of sequence identity that could be set to annotate reads with a certain degree of confidence, based on the increase of the number of reads and taxa (considering both genus and species for each organism group) by decreasing the identity of match from 100% to 50% (Fig 1; Figure A in S1 File). Sequence identity from which there was no increase of information (in terms of assigned reads and identified taxa) ranged from 88% (fungi, for number of species) to 73% (bacteria, for number of reads at the species and genus levels; Table 2). That means that the database used to annotate the obtained sequence reads was able to capture a quite precise metagenomic signature contained in the honey.

**Table 2:** Lowest percentage of sequence identity below which the comparison against the NCBI nt database did not report any decrease in the number of annotated reads and identified taxa.

	Orange tree blossom honey				Eucalyptus tree blossom honey			
	Level: species		Level: genus^‡^		Level: species		Level: genus^‡^	
Datasets	Reads	Taxa	Reads	Taxa	Reads	Taxa	Reads	Taxa
Arthropods	77^†^	83	77	87	75	77	75	77
Plants	78	78	78	81	77	77	77	79
Fungi	77	88	77	82	75	75	75	78
Bacteria	74	74	74	76	73	75	73	75
Viruses	78	87	-	-	75	81	-	-

^†^ Percentage of sequence identity.

^‡^ Data are not reported for viruses since the genus level is missing for some of them, leading to unreliable statistics.

### Taxonomic profiles and richness

To report the results divided by genus and species for the five organism groups, a few sequence identity levels were set up (Table A in S1 File): <75%, that means that no threshold was considered; ≥75% that corresponded to the identity that was able to annotate the largest number of reads and taxa in most organisms/species/genus combinations; ≥97% that would consider almost identical sequences, accounting also for a 3% of sequencing error rate of the Ion Torrent platform (according to the average error rate calculated from previous experiments [34, 35].

**Fig 2:**
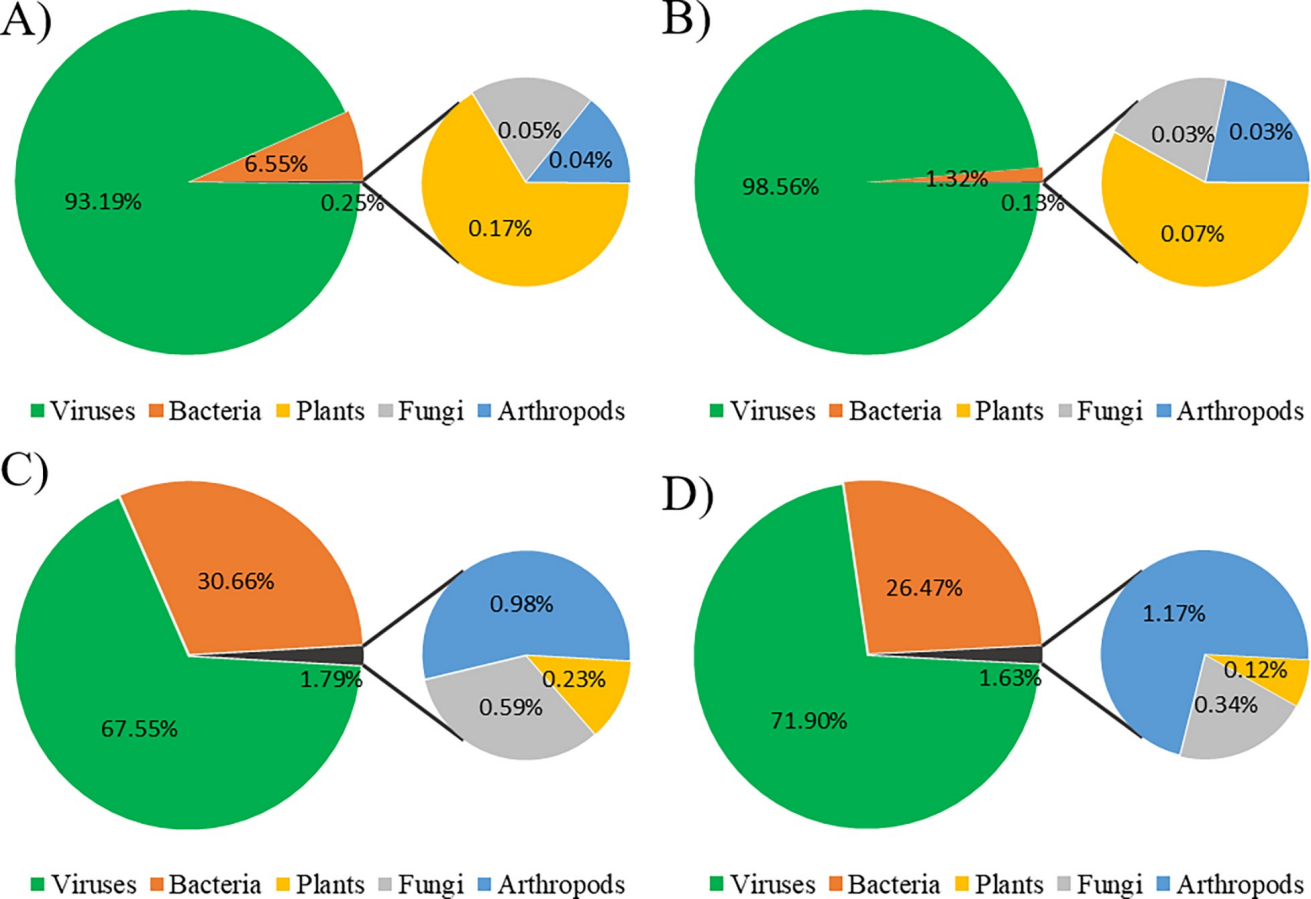
Proportion of reads assigned to the five organism groups in the orange tree and eucalyptus tree blossom honeys using different sequence identity. Orange tree blossom honey: sequence identity ≥ 75% (A) and ≥ 97% (B); eucalyptus tree blossom: sequence identity ≥ 75% (C) and ≥ 97% (D). Percentages are computed considering only reads annotated at the taxonomic rank of species (see Table 3).

Table 3 reports the distribution of the number of distinct genera and species that could be identified from the mapped reads using the three thresholds of sequence identity. It was quite surprising to note that the largest number matched virus sequences in both honey samples (see below for a detailed description; Table 3 and Table A in S1 File). This was evident without setting any defined threshold for sequence identity (91.75% and 65.03% of total reads in the orange tree blossom honey and in the eucalyptus tree blossom honey, respectively) as well as considering all other identity thresholds (Table 3 and Table A in S1 File; Fig 2 and Figure B in S1 File). The second most represented kingdom in both honeys was bacteria for which the fraction of mapped reads ranged from 7.42% and 32.31% (for the two honeys without any sequence identity threshold) to 1.52% and 27.33% (for the same samples but setting the 97% sequence identity threshold). The other organism groups were (in decreasing order of number of mapped reads): plants, fungi and arthropods, for the orange tree blossom honey (at all sequence identity thresholds); fungi, arthropods and plants (considering no thresholds for read mapping) or arthropods, fungi and plants (with the 97% threshold for read mapping) for the eucalyptus tree blossom honey (Fig 2).

**Table 3:** Number of putative MOTUs at the species and genera levels characterizing the two honey samples. Data are presented for each dataset, considering three different thresholds of sequence identity (<75%, that means without any threshold; ≥ 75%; ≥ 97%).

Honey		Orange tree blossom honey						Eucalyptus tree blossom honey					
Sequence identity		< 75%		≥ 75%		≥ 97%		< 75%		≥ 75%		≥ 97%	
Dataset^†^	Level	N. of reads	N. of MOTUs	N. of reads	N. of MOTUs	N. of reads	N. of MOTUs	N. of reads	N. of MOTUs	N. of reads	N. of MOTUs	N. of reads	N. of MOTUs
**Arthropods**	**Species**	96	23	96	23	53	7	2500	58	2497	56	1620	20
	**Genus**	104	21	104	21	58	7	2709	42	2706	40	1793	11
**Plants**	**Species**	442	98	442	98	141	38	579	89	578	88	165	36
	**Genus**	538	86	538	86	209	38	614	74	613	73	191	32
**Fungi**	**Species**	129	42	129	42	49	13	1497	101	1495	101	467	40
	**Genus**	180	33	180	33	59	12	2747	54	2746	54	958	23
**Bacteria**	**Species**	17465	688	17452	683	2514	121	78332	846	78199	834	36648	229
	**Genus**	19867	280	19843	278	2834	65	83343	321	83192	318	37939	92
**Viruses**	**Species**	248139	4	248139	4	188360	3	172260	14	172258	14	99540	4
** **	**Genus**^**1**^	-	-	-	-	-	-	-	-	-	-	-	-

^†^ Data are not reported for viruses since the genus level is missing for some of them, leading to unreliable statistics.

Rarefaction curves reached the plateau for a few organism groups for which a lower number of species or genera was identified (i.e. arthropods, viruses and fungi) but not for the other groups (Figures C-D in S1 File). This might be due to the low sequencing depth that we applied. Species richness index (Chao1) was higher in the eucalyptus tree blossom honey for all organism groups, except viruses for which it was the same (Table B in S1 File). Eucalyptus tree blossom honey was richer than the orange tree blossom honey in terms of overall number of genera and species at all sequence identity levels for most of organism groups (Table 3). For example, the number of distinct bacteria and fungi species and genera were almost the double in the eucalyptus tree blossom honey than in the other honey. It was interesting to note that virus sequences (that accounted for the largest number of sequenced reads) were derived from one (orange) or three (eucalyptus) species only (see below).

### Characterization of reads at different taxa levels

The most represented species and genera for each of the five main organism groups are reported in Table 4 and Table C in S1 File. For sake of clarity, the two tables list MOTUs counting each one at least the 5% of reads over the whole set of reads annotated within a specific organism group, and having at least 75% of sequence identity. Complete lists of identified species and genera considering the ≥75% and ≥97% sequence identity levels are reported in Tables D-G in S1 File. The subsequent descriptions are referred to the ≥75% sequence identity level, unless otherwise stated.

**Table 4:** Predominant species identified in the two analyzed honeys considering the ≥75% sequence identity level based on reads accounting >5% overall reads of the identified organism groups.

Honey	Dataset	Taxid		Scientific name	N. of reads	% of reads^†^	‰ of reads_TOT_^‡^
**Orange tree blossom honey**	**Arthropods**	7460		*Apis mellifera*	41	39.05	0.15
		7176		*Culex quinquefasciatus*	8	7.62	0.03
		28612		*Rhagoletis zephyria*	8	7.62	0.03
		7029		*Acyrthosiphon pisum*	7	6.67	0.03
	**Plants**	2711		*Citrus sinensis*	72	11.92	0.27
		29760		*Vitis vinifera*	51	8.44	0.19
		85681		*Citrus clementina*	41	6.79	0.15
		3880		*Medicago truncatula*	37	6.13	0.14
	**Fungi**	1108849		*Penicillium rubens*	27	14.52	0.10
		4956		*Zygosaccharomyces rouxii*	19	10.22	0.07
		500148		*Metarhizium brunneum*	15	8.06	0.06
	**Bacteria**	1906742		*Microbacterium sp*. *BH-3-3-3*	3,479	17.36	12.86
		2033		*Microbacterium testaceum*	3,075	15.34	11.37
		148814		*Lactobacillus kunkeei*	1,989	9.92	7.35
	**Viruses**	1100043		*Apis mellifera filamentous virus*	248,133	100.00	917.52
**Eucalyptus tree blossom honey**	**Arthropods**	7460		*Apis mellifera*	1,820	66.50	6.87
		7461		*Apis cerana*	165	6.03	0.62
		597456		*Habropoda laboriosa*	162	5.92	0.61
	**Plants**	71139		*Eucalyptus grandis*	185	28.20	0.70
		223129		*Orobanche rapum-genistae*	112	17.07	0.42
		4686		*Asparagus officinalis*	55	8.38	0.21
	**Fungi**	4956		*Zygosaccharomyces rouxii*	456	16.26	1.72
		1365886		*Zygosaccharomyces parabailii*	282	10.05	1.06
		4896		*Schizosaccharomyces pombe*	225	8.02	0.85
	**Bacteria**	148814		*Lactobacillus kunkeei*	37,752	44.11	142.51
		542		*Zymomonas mobilis*	11,721	13.70	44.25
	**Viruses**	1100043		*Apis mellifera filamentous virus*	172,213	99.97	650.10

^†^ Data considers as background the specific number of reads representing the five subsets and the honeys, as reported in Table A in S1 File (columns “sequence identity <75%”).

^‡^ Data considers as background the total number of annotated reads of that honey, as reported in Table A in S1 File (columns “sequence identity <75%”).

#### Arthropods

As expected, *Apis mellifera* was the species with the largest number of arthropod reads in both honeys. *Apis* genus accounted for 52.38% of reads in the orange tree blossom honey and 82.94% of reads in the eucalyptus tree blossom honey. Reads identified also other insects in both analysed samples. A few of them might potentially be considered as false positives, probably derived by high homology of their genome with parts of the *A*. *mellifera* genome. For example, reads assigned to *A*. *cerana*, *A*. *dorsata* and *A*. *florea* from the eucalyptus tree blossom honey might be due to this problem. This could also be (at least in part) the reason for which a few other species of the Apidae family have been captured by this shotgun metagenomic analysis (including read assigned to the southeastern blueberry bee *Habropoda laboriosa*, that is mainly distributed in the United States; Table 4 and Tables D-G in S1 File). However, the identification of reads of several other Apidae species (some of which present in the investigated honey area: i.e. *Megachile rotundata* in both honeys; Tables D-G in S1 File) together with species of the order Diptera, that are well known to take sugar meals from plants, might extend the interpretation of the honey eDNA signature that is able to capture information from insects that feed on sugar rich plant derived secretions that might be used by honey bees as source of sugar material for the subsequent production of honey. This is also demonstrated by the presence of reads assigned to *Acyrthosiphon pisum* (>5% of reads from the orange tree blossom honey) and other aphids (Table 4 and Tables D-G in S1 File) that confirmed that it is possible to identify in the honey DNA the signature of plant-sucking insects that produced honeydew that was collected by the honey bees that, in turn, produced the honey [18]. Other reads were from Lepidoptera (including *Galleria mellonella*, known as greater wax moth or honeycomb moth, a damaging moth living in the hives), a few Coleoptera and Aracnidae species, including *Varroa destructor* (the most damaging honey bee parasite) and the pink citrus rust mite *Aculops pelekassi* (an important eriophyoid pest of commercial citrus plants), identified in the orange blossom tree honey (Tables D-G in S1 File).

#### Plants

The plant species and genera that matched the largest number of reads in the two honeys were in agreement with their botanical origin. *Citrus sinensis* (i.e. orange tree; 11.92% of plant reads) or *Citrus* spp. (31.62% of plant reads) were the most represented species and genera in the orange tree blossom honey whereas *Eucalyptus grandis* (28.20% of plant reads) or *Eucalyptus* spp. (32.16% of plant reads) were the most represented species and genera in the eucalyptus tree blossom honey (Table 4 and Tables D-G in S1 File). These results also confirm the metabarcoding data obtained from the analysis of the chloroplast *trnL-UAA* DNA region on the same two honeys that however obtained, respectively, a resolution at the family (Rutaceae) or subfamily (Myrtoideae) levels for their most important floral contributors [18]. Metagenomic derived reads assigned to other plants provided signatures of the honey bee foraging areas that were recovered from the analysed honeys. The botanical signature obtained for the eucalyptus tree blossom honey was close to that previously obtained with a metabarcoding approach [18]. The two other most represented genera, *Orobanche* spp. and *Asparagus* spp., accounting for 21.65% and 8.54% of plant reads in the current experiment, were reported to be among the second (17.4% of *trnL-UAA* reads) and the fifth (6.4% of the indicated chloroplast reads) plant groups in the metabarcoding study [18]. The results reported by Utzeri et al. [18] indicated that the genus *Oxalis* was the second most represented group of plants in the orange tree blossom honey in addition to several other minor plants (in terms of number of reads) that were not reported or that accounted for just few reads in the shotgun metagenomic analysis. However, the metagenomic analysis captured *Vitis* and *Medicago* as the two genera with more than 5% of total plant reads in this honey (Table C in S1 File).

#### Fungi

A larger number of fungus reads were identified in the eucalyptus tree blossom honey than in the other honey (Tables 3 and 4; Tables D-G in S1 File). These fungi could be classified according to their main role/action or prevalent ecological niche and possible origin:

1. A few fungi (yeasts) are well known for their high sugar-concentration and ethanol tolerance and were already described in honey isolates. Some of them are fermentative agents involved in the aroma production of food and beverages [40, 41, 42]; *Zygosaccharomyces* was the most represented genus in both honeys (about 42% and 60% of reads, respectively), with reads mainly matching a few species (*Z*. *mellis*, *Z*. *parabailii* and *Z*. *rouxii*); *Schizosaccharomyces* spp. accounted for more than 5% of reads in the eucalyptus tree blossom honey; several reads were from *Lachancea*, *Saccharomyces* and *Torulaspora* spp.
2. Others are known as direct or indirect honey bee or insect pathogens: *Metarhizium* spp. (a genus of entomopathogenic fungi) accounted for more than 5% of reads in the orange tree blossom honey; other fungi of this group listed: *Nosema ceranae*, the nosemiasi agent; *Bettsia alvei*, the beehive mold that growths on stored pollen; and *Aspergillus* spp., which can infect brood causing stonebrood.
3. Some of them are plant pathogens: *Alternaria alternata*, an opportunistic ascomycete causing leaf spots on a large number of plant species and one of the most common airborne fungi in many environments; *Ustilago bromivora*, a biotrophic smut fungus infecting *Brachypodium* spp.; *Botrytis cinerea*, that affects many different plants and commonly known in viticulture as the agent of the botrytis bunch rot).
4. A few fungi might play defensive function: *Penicillium* spp. (well known for the production of penicillin) accounted for more than 5% of reads in the orange tree blossom honey.

#### Bacteria

**Fig 3:**
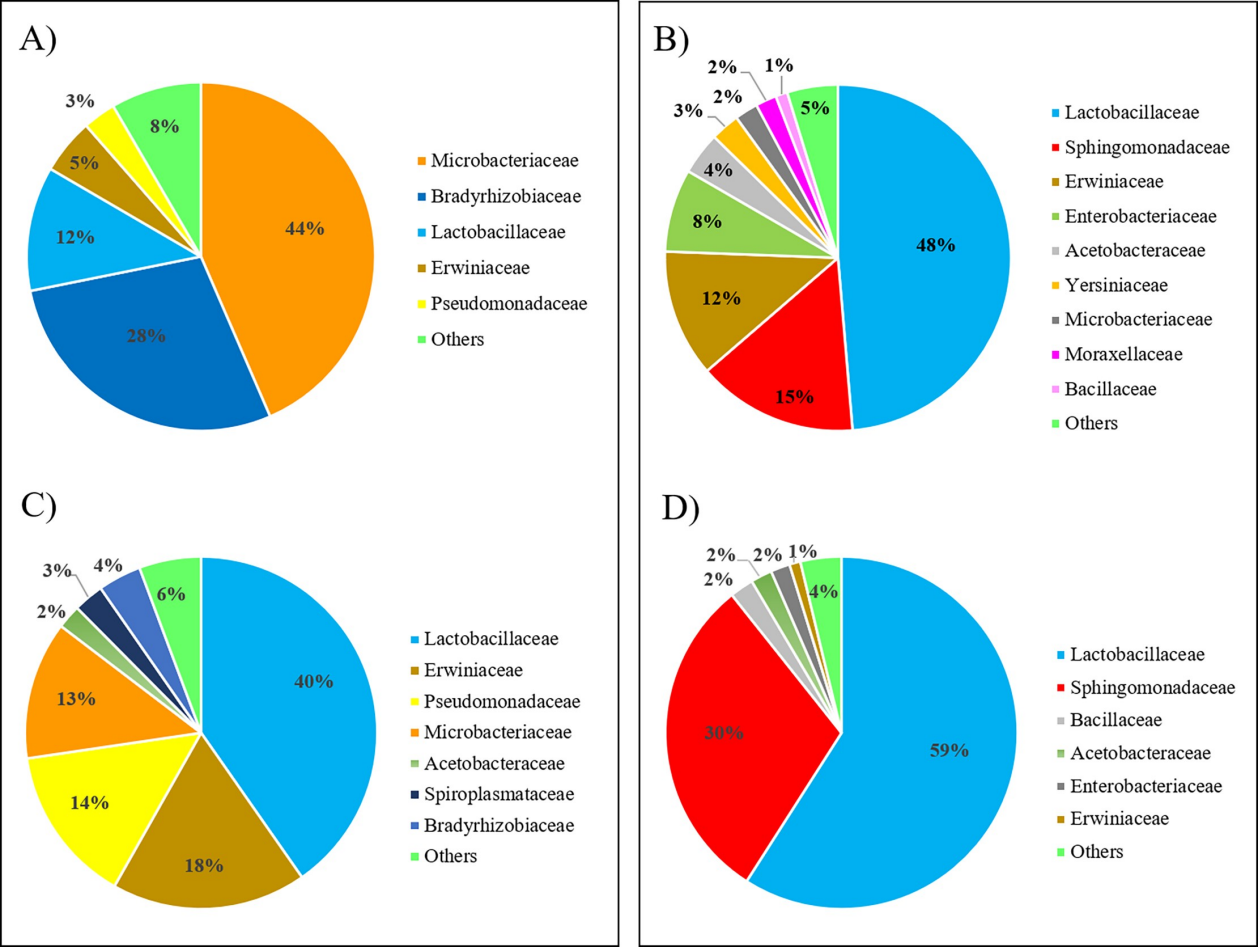
Proportion of reads assigned to distinct bacteria families (derived by the sum of reads of the species belonging to the family) in the two analyzed honeys. Orange tree blossom honey: sequence identity set at ≥75% (A) and ≥97% (C); eucalyptus tree blossom honey: sequence identity set at ≥75% (B) and ≥97% (D). “Others” indicates all other families detected that did not pass the 1% of reads over all bacteria reads.

They represented the largest number of species and genera in the analyzed samples (Table 3 and Table 4). The two honeys were however quite different in terms of bacterial signatures that, to some extent, may further enlarge the microorganism differences evidenced for the fungi. To obtain an overall picture, reads assigned to distinct species at the two sequence identity thresholds (75% and 97%) were, re-grouped at the family level (Tables D-G in S1 File; Fig 3; Figure E in S1 File). A total of 105 and 123 families were identified in the orange tree blossom honey and in the eucalyptus tree blossom honey, respectively. Only five and nine families accounted each for at least 1% of the overall bacterial reads reported in the two samples (Fig 3; Figure E in S1 File). Microbacteriaceae and Lactobacillaceae were the most represented families (in terms of identified reads) in the orange and eucalyptus tree blossom honeys, respectively. A more detailed analysis of reads assigned to the bacteria kingdom (i.e. going down to the species level and using the 97% identity threshold to be more precise in the identification) could obtain a preliminary vision of the putative prevalent origin or role of the bacterial signatures in the two honeys. In the orange tree blossom honey, at this level of identity, the largest number of reads were assigned to four species (*Lactobacillus kunkeei*, ~~5%; *Pantoea agglomerans*, ~2%; *Pseudomonas syringae*, ~~1.5%; and *Microbacterium testaceum*; ~~1.0%), that in absolute terms accounted for more than 100 reads each (Tables D-G in S1 File). In the eucalyptus tree, the species that accounted for more than 100 reads were 11, among which *L*. *kunkeei* was again the most represented species (contributing for more than 25% of bacterial reads in this honey), followed by *Zymomonas mobilis* (~13% of bacterial reads) and *Bacillus cereus* (~~0.9% of bacterial reads). Similar to the fungi, it could be possible to group these bacteria, combining their main role/action or prevalent ecological niche and their putative origin:

1. typical bacteria of the hive micro-environment, adapted to the high sucrose concentration of the honey (also common components of the honey bee gut microbiota): we can list *L*. *kunkeei* (the most represented bacteria in both honeys; an obligate fructophilic lactic acid bacterium, found in fructose-rich niches), *Parasaccharibacter apium*, *Gilliamella apicola*, *Frischella perrara*, among several other species, that are common microorganisms of the honey bee gut microbiota;
2. honey bee pathogens: *Melissococcus plutonius* (the aetiological agent of the European foulbrood disease), frequent in the eucalyptus tree blossom honey (identified with >150 reads); *Spiroplama apis* (the agent of the spiroplasmosis, identified in the eucalyptus tree blossom honey: 97% sequence identity); and *Paenibacillus larvae* (determining the American foulbrood, identified in both honeys);
3. plant pathogens: *Pseudomonas syringae* (a ubiquitous pathogen that can infect a wide range of plant species), *Erwinia amilovora* (the fire blight agent), *Spiroplasma citri* (the causative agent of Citrus stubborn disease, identified in the orange tree blossom honey) among several other species that play pathogenic actions against plants;
4. ubiquitous and specialized species: i.e. *Escherichia coli*, *Bacillus cereus*, *Salmonella enterica* that present several strains, some of which relevant in food safety;
5. antagonistic bacteria like *P*. *agglomerans*, that is a ubiquitous bacterium commonly found on plant surfaces and throughout a honeybee's environment, thought to have possible actions against the fire blight agent *Erwinia amylovora* [43].

#### Viruses

As mentioned above, almost all virus reads were assigned to just one species: *Apis mellifera* filamentous virus (AmFV). This is a honey bee ubiquitous dsDNA virus that affects many apiaries throughout Europe with mild pathogenetic effects [44, 45]. Sequenced reads covered almost completely the 0.5 Mbp of the AmFV genome with a depth of about 91X in the orange tree blossom honey and 67X in the eucalyptus tree blossom honey, with a coverage higher than 99% in both honeys. Aligning the obtained reads to the AmFV genome sequence (GenBank accession number KR819915), a quite large number of sequence differences could be identified: in the orange tree blossom honey we identified a total of 3,126 single nucleotide polymorphisms (SNPs) and 644 indels and in the eucalyptus tree blossom honey we identified 20,526 SNPs and 1,835 indels for a total of 20,842 SNPs and 2,075 indels discovered across the two samples (2,810 SNPs and 408 indels were in common in the two honeys). Few reads also matched other virus sequences (in the orange tree blossom honey: *Spiroplasma* virus SVTS2 and *Heliothis virescens ascovirus 3a*; in the eucalyptus tree blossom honey: *Musca hytrovirus*, *Lactobacillus* virus Lb338-1, *Erwinia* phage phiEt88) that might provide additional environmental or ecological signatures of the hive microorganisms.

## Discussion

Honey bees, during their activities, visit so many niches and, for this reason, they can come into intimate contact with many environmentally derived sources of DNA. This DNA might define a complex eDNA signature that, in turn, is transferred to the honey. Honey accumulates not only external DNA but also reflects the superorganism ecology and internal hive conditions by acquiring DNA from all biological components that in some way have had relationships or contributed to the first definition, maturation and then final production of this sucrose-rich honey bee product. Honey tends to be transformed, at least in part, by its microorganism composition over time [40]. Our study, demonstrated that a low-density shotgun metagenomic experiment can disclose the multi-kingdom signature residing in the honey DNA. We tested the possibility to interpret data derived by a low-density shotgun metagenomic analysis to verify the usefulness of this cost-effective approach for its envisaged routine application on a larger number of honey samples. An extended use would be important for the development of innovative biomonitoring tools that could investigate, from one hand, the factors affecting colony collapse disorder (that is jeopardizing the apiculture sector worldwide; i.e. [46, 47]) and, from the other hand, could capture other environmental information taking advantage from the explorative efficiency of the honey bees. In their activities, honey bees can reach inaccessible sites and cover a quite large area during the foraging flights [13, 14].

The interpretation of metagenomic data mainly relies in the completeness of the sequence database that is used for the bioinformatic analyses. This is at least in part demonstrated by the results that we obtained. For example, plant assigned reads in the honeys confirmed their predominant botanical origin, i.e. orange tree and eucalyptus tree, respectively. The genomes of *Citrus sinensis* (sweet orange tree) and *Eucalyptus grandis* (eucalyptus tree) are already sequenced and the NCBI nt database that was used for the comparison includes many entries covering the whole published sequenced genomes of these species [31, 32]. The availability of these data increased the probability to assign randomly sequenced metagenomic fragments, derived by the honey DNA (of pollen origin), to regions of the genome of these plant species. It was interesting to note that the proportion of plant reads matching the orange tree and the eucalyptus tree genomes was very close to the proportion of reads assigned to these plant species in a metabarcoding experiment we did on the same two honeys [18]. These results indicate that, at least for honeys that have as prevalent botanical composition a plant for which the genome is already completely sequenced (in our cases, the orange and eucalyptus trees), a low density (and for this reason cost-effective) metagenomic analysis may become a convenient alternative to the metabarcoding approach to identify the dominant floral composition. It would be interesting to evaluate the correlation between metabarcoding sequencing and this cost effective metagenomic analysis also for the organisms of other kingdoms, i.e. microbiota analysis using 16S targets.

The sequence identity that defined a minimum threshold to map a read was evaluated. The adopted approach was based on the increase of the number of taxa that could be identified by reducing the sequence identity percentage. This aspect might be relevant in the assignment process of reads to the corresponding taxa and then for the fine interpretation of the results. This approach was able to annotate many reads but with a low resolution (in term of taxa identification) that we could accept as a first descriptive step, considering the incompleteness of the sequence database available at present and used for data comparison. As expected, when the stringency was increased (i.e. 97% of sequence identity to assign a species) the number of species detected decreased whereas the confidence in species richness estimates increased. These results were refined for a more precise evaluation in all organism groups. Other species could be identified increasing the sequencing depth, even if it seems that the most important species-derived signature was captured in both honeys.

The interpretation of the derived eDNA signature was complicated by the various sources from which the DNA originated. Microbial signature in the honey derives from the hive ecology and pollination landscape [48]. The shotgun metagenomic approach of honey DNA was able to capture microorganism signatures similarly to those described by metabarcoding studies of the honey bee gut microbiota [49, 50]. Fungi and bacteria signatures could be grouped according to the role and origin of these microorganisms considering a few characteristics: i) microorganisms that commonly live in the hive (of both external and/or honey bee gut origin), that are well adapted to this environment or to the high sucrose concentration of the honey (and that may contribute to give to this food product its aroma variety or its properties); ii) microorganisms of environmental origin that might be relevant in food safety (some of which are part of the previous categories); iii) microorganisms with some antagonistic effects against pathogenic organisms, that could be important to control or modulate the health status of the honey bee superorganism; iv) microorganisms that are plant pathogens that are collected by honey bee workers during their foraging activities and v) microorganisms that are considered honey bee pathogens.

Honey bee symbiont microorganisms might become central to studies on honey bee health and to understand the factor affecting colony collapse disorder [51]. Most of the potential microorganisms relevant in food safety are expected to be in inactive forms as they cannot survive in honey because of its several properties including hygroscopicity, hyperosmolarity, antibiotic content and activities of some other microorganisms, among other properties. The presence of antibiotic producing microorganisms might support therapeutic potential properties of some honeys [52]. The possible role or involvement of the honey bees in the transfer and monitoring of plant pathogen microorganism distribution has been already reported and discussed by other authors [53–55]. The presence of plant pathogen reads could be integrated with information from plant pests (insects or other arthropods; i.e. this study identified *Aculops pelekassi*, that damages Rutaceae species) and used to model plant disease epidemiological levels in the environment. The same could be applied considering the organisms involved in apiary diseases integrating microorganisms, arthropods (reads belonged to the most important honey bee parasite, *Varroa destructor*, and to the greater wax moth) and viruses. The virus signature was mainly determined by one highly represented virus (AmFV). This virus is considered of low pathogenic impact on honey bees even if it has been associated with winter honeybee colony loss in a few occasions and has been reported in association with *Nosema apis* [45]. The high level of AmFV reads that we obtained in both honeys is quite puzzling and needs further investigations to understand the reason for which DNA of this virus was highly represented in the shotgun sequencing experiments.

All these results provided a multi-kingdom host-pathogen signature that could pave the way for the definition of a comprehensive eDNA derived colony health index that would account for all possible disease-causing organisms present in the hive. Correlation with other colony health parameters would be important to consider honey eDNA derived information as useful indicators or predictors of colony collapse disorder.

The description of eDNA information assigned to non-honey bee insects might open interesting opportunities on the use of honey DNA to monitor the environmental distribution and density of other members of the order Hymenoptera and Diptera that might leave unexpected traces in honey. These traces could probably be derived from the common sucrose rich sources visited by both honey bees and the other insects. This aspect need further analyses and comparisons with metagenomic data that would be captured from a higher sequencing depth.

## Conclusions

This study demonstrated that honey eDNA can be used to obtain an overall picture of the colony ecosystem and of the landscapes from which honey bees take their nutrients. Using this source of information, it is possible to extend the usefulness of honey bees as biomonitoring tools. In addition, the reported method and the obtained results could open new avenues to describe the honey bee superorganism pathosphere that might be useful to understand factors involved in the colony collapse disorder.

The shotgun metagenomic approach that was used in this study can identify all possible organisms that in some way interact or define the honey production activities and phases, overcoming the specialization of metabarcoding methods that are usually designed to generate sequence information for just a group of organisms (i.e. bacteria, fungi or others). The depth of sequencing could be a limiting factor together with an incomplete reference database that would not be able to annotate all obtained sequences. However, a low-density shotgun metagenomic analysis might be cost-effective considering both the sequencing cost and the data analysis step. Therefore, it seems that the approach tested in this study could be applied in large scale experiments that can have multiple objectives according to the multi-kingdom derived eDNA that is contained in honey.

## Supporting information

S1 File Supplementary material: Tables A-G and Figures A-E.
(DOCX)
