# Supplementary material for: Shotgun metagenomics of honey DNA: Evaluation of a methodological approach to describe a multi-kingdom honey bee derived environmental DNA signature
Source: PLoS One. 2018 Oct 31;13(10):e0205575. doi: 10.1371/journal.pone.0205575 (PMC6209200; doi:10.1371/journal.pone.0205575)
Supplement: S1 File — Supplementary material: Tables A-G and Figures A-E. (DOCX) [file pone.0205575.s001.docx]

**Supplementary File 1**

**Shotgun metagenomics of honey DNA: evaluation of a methodological approach to describe a multi-kingdom honey bee derived environmental DNA signature**

Samuele Bovo, Anisa Ribani, Valerio Joe Utzeri, Giuseppina Schiavo, Francesca Bertolini, Luca Fontanesi

**Figure A.** Effect of minimum sequence identity cut-off values on the number of identified taxa (considering the genus and species levels). A) Orange tree blossom honey, genus level; B) Orange tree blossom honey, specie level; C) Eucalyptus tree blossom honey, genus level; D) Eucalyptus tree blossom honey, specie level. Colours are as follow: blue, arthropods; yellow, plants; gray, fungi; orange, bacteria; green, viruses. Data are not reported for virus at the genus level since this level is missing for some of them, leading to unreliable statistics. Plots are reported for a sequence identity ranging from 100% to 70% (from the left to the right) as lower values did not change the number of assigned sequences (as evidenced from the plateau reached in all plots for all organism groups).


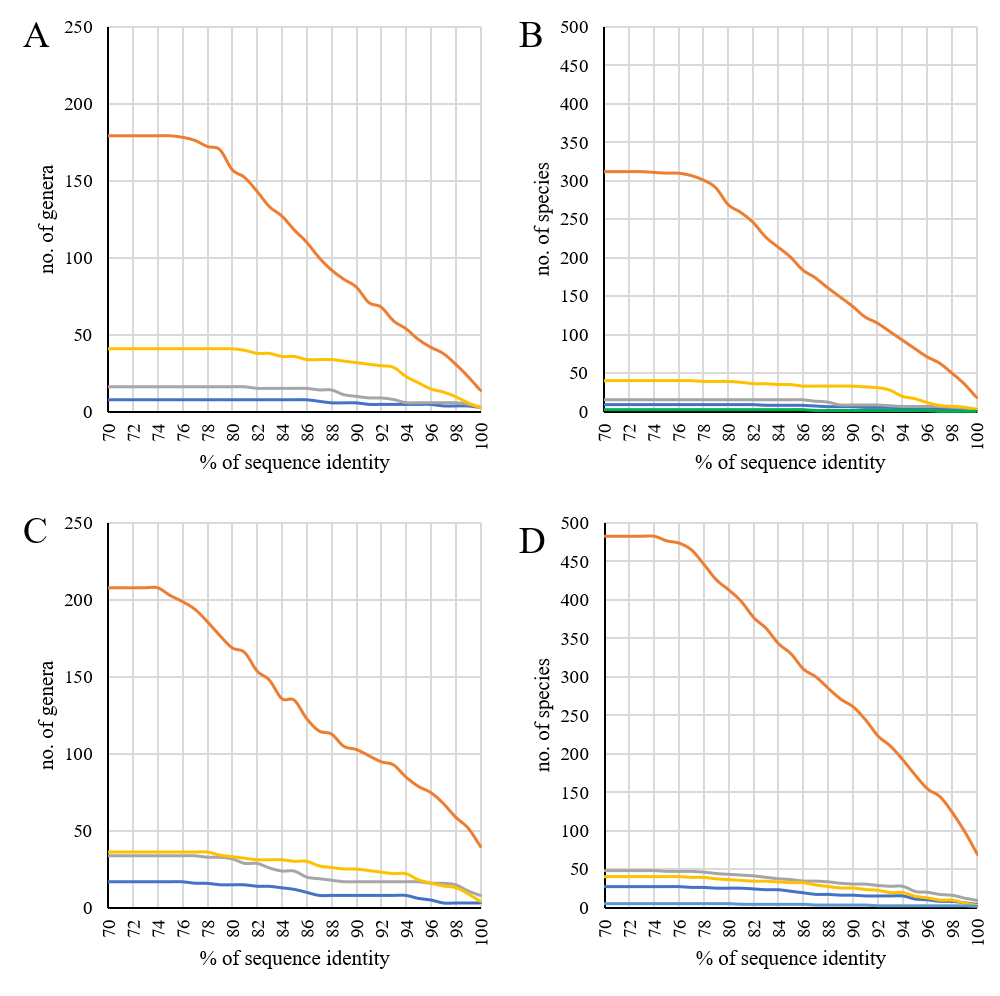


**Figure B.** Proportion of reads assigned to the five “organism groups” in the orange tree blossom honey using sequence identity ≥ 75% (A) and ≥ 97% (B) and in the eucalyptus tree blossom honey using sequence identity ≥ 75% (C) and ≥ 97% (D). Percentages are computed considering reads annotated to each organism group independently from the taxonomic rank (Table A).


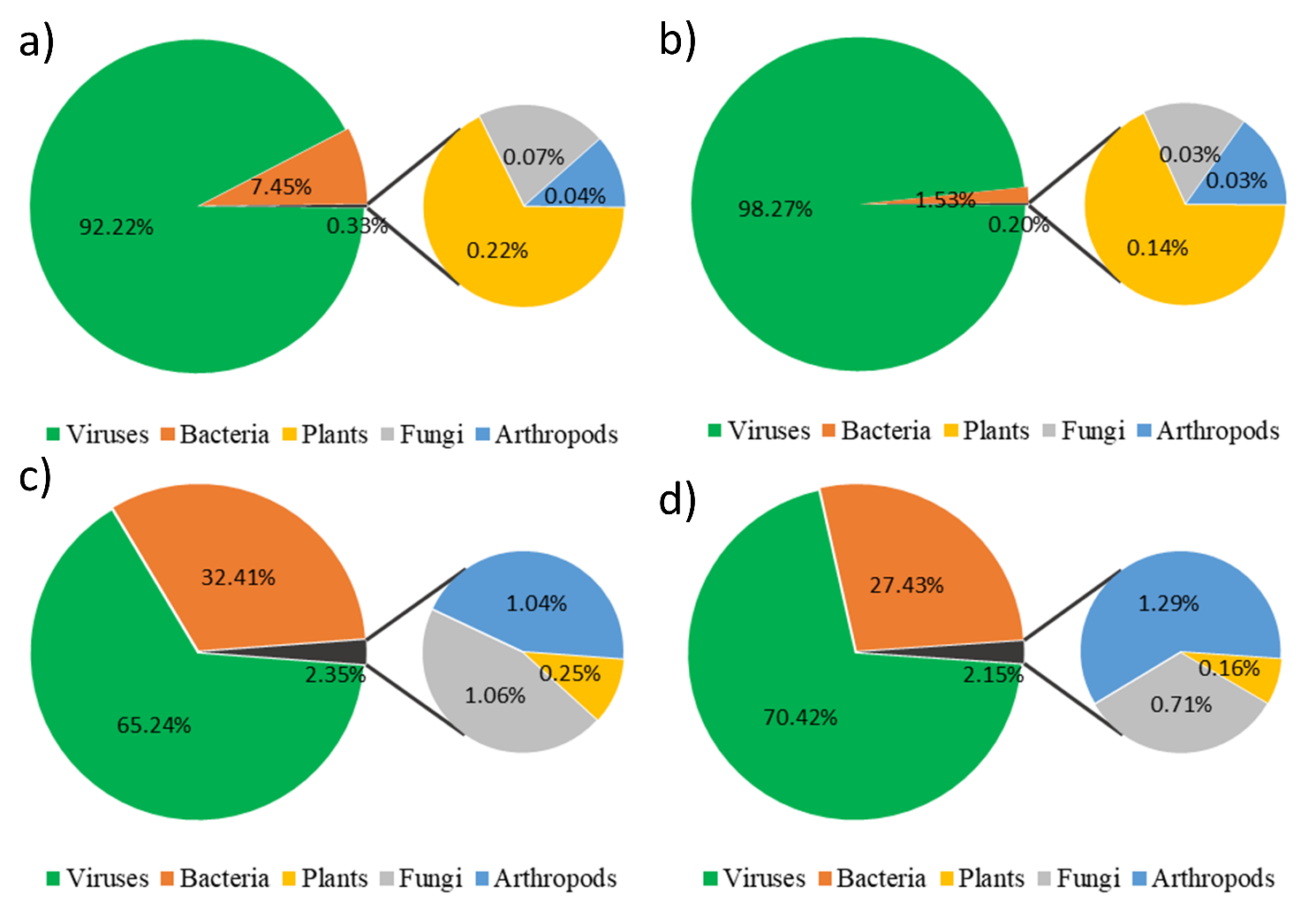


**Figure C.** Rarefaction curves at sequence identity ≥ 75%. Each curve has been built considering only the reads belonging to each organism group. A) Orange tree blossom honey, genus level; B) Orange tree blossom honey, species level; C) Eucalyptus tree blossom honey, genus level; D) Eucalyptus tree blossom honey, species level. Colours are as follow: blue, arthropods; yellow, plants; gray, fungi; orange, bacteria; green, viruses. Data are not reported for virus at the genus level since this level is missing for some of them, leading to unreliable statistics.


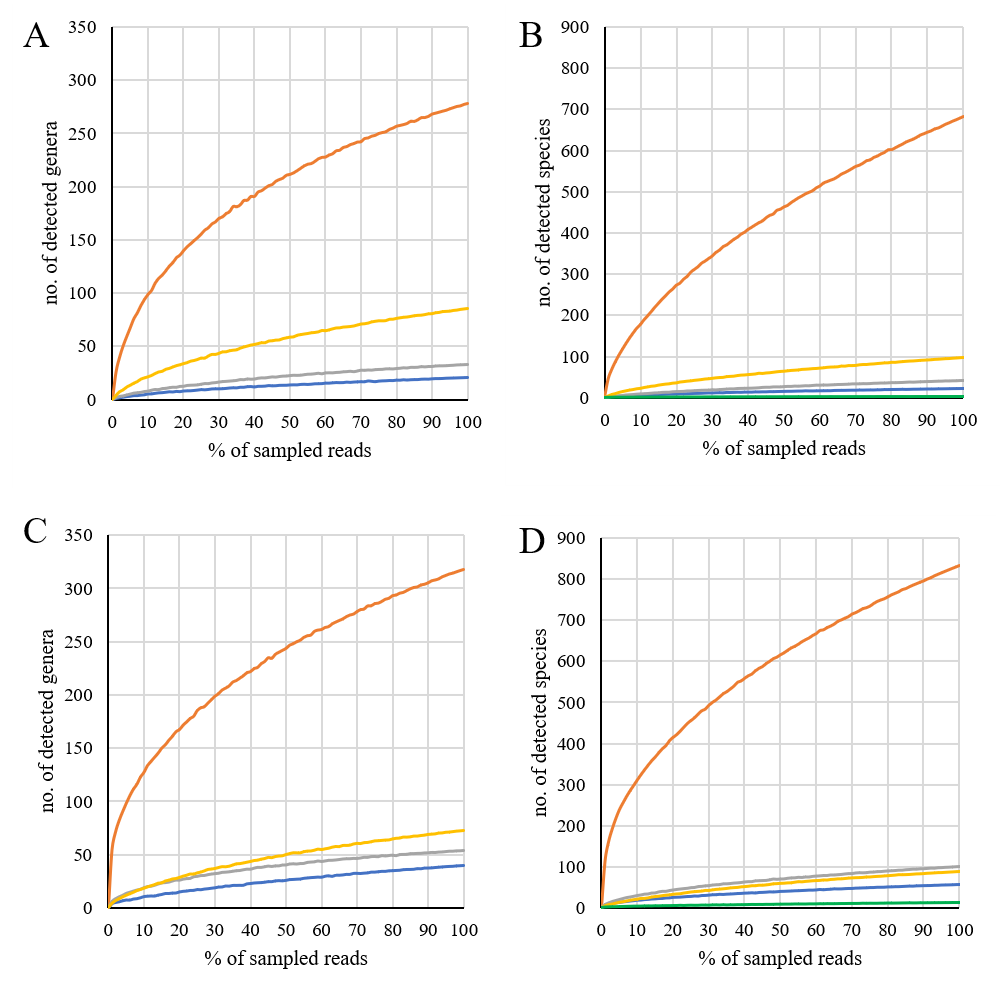


**Figure D.** Rarefaction curves at sequence identity ≥ 97%. Each curve has been built considering only the reads belonging to each organism group. A) Orange tree blossom honey, genus level; B) Orange tree blossom honey, species level; C) Eucalyptus tree blossom honey, genus level; D) Eucalyptus tree blossom honey, species level. Colours are as follow: blue, arthropods; yellow, plants; gray, fungi; orange, bacteria; green, viruses. Data are not reported for virus at the genus level since this level is missing for some of them, leading to unreliable statistics.


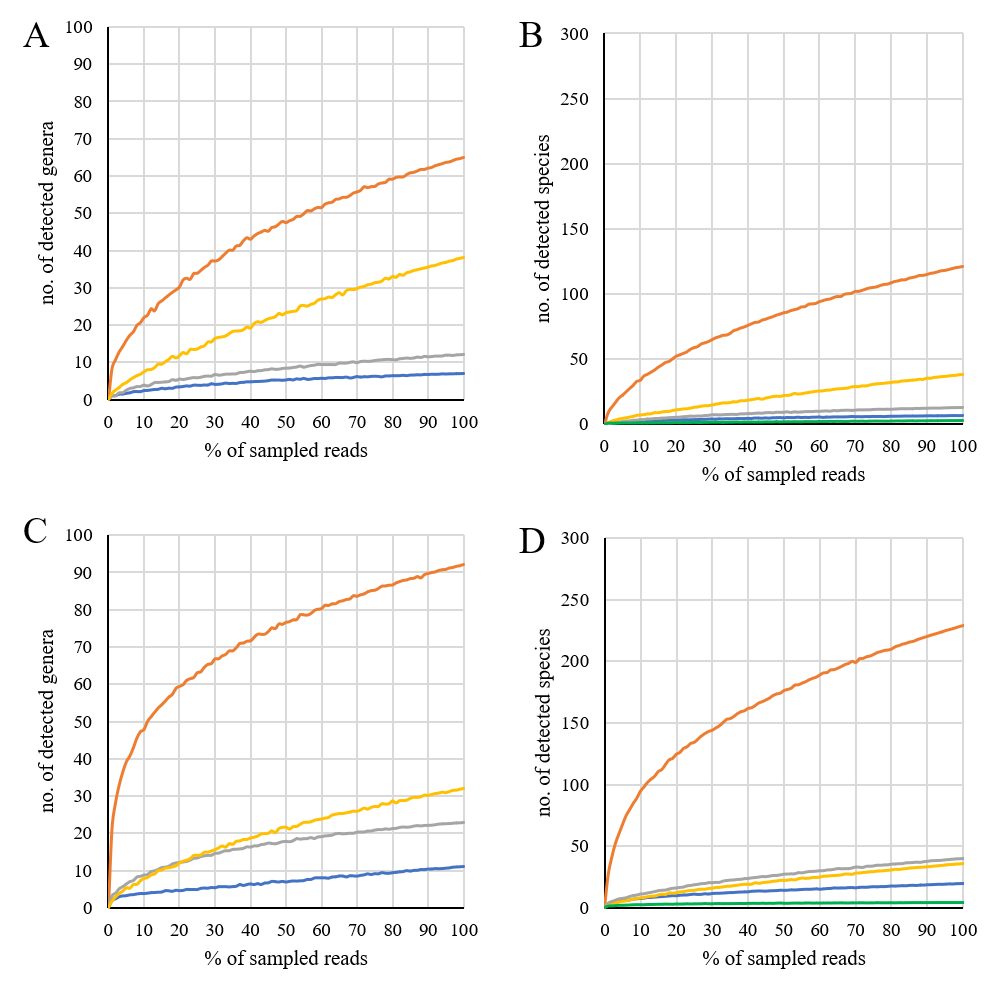


**Figure E.** Phylogenetic relationships of bacteria families for which reads (reported as proportion of reads assigned to distinct families, as derived by the sum of reads of the species belonging to the family) were identified in the orange tree blossom honey (OTBH) and in the eucalyptus tree blossom honey (ETBH) using sequence identity (SI) set at ≥75% and ≥97%.

**
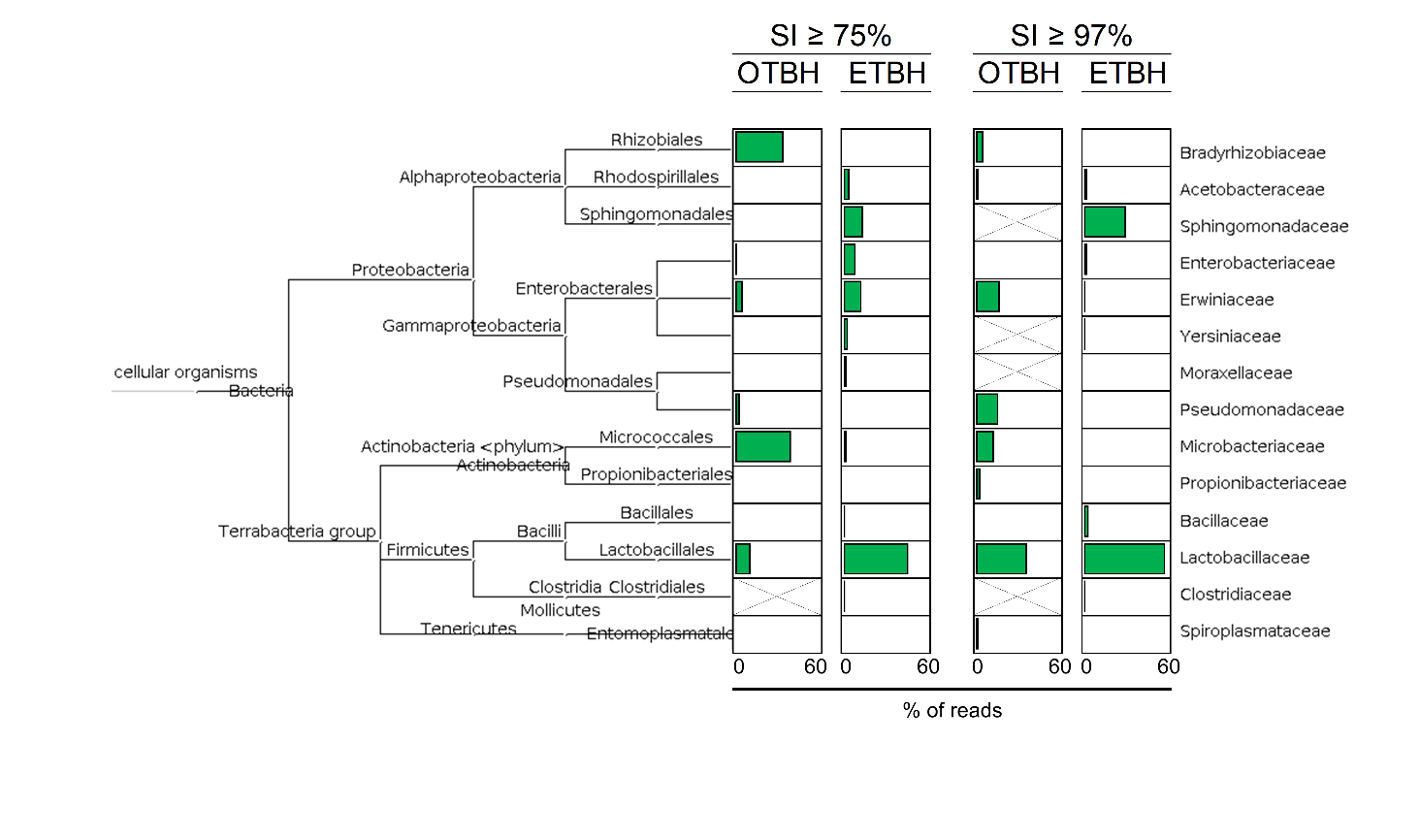
**

**Table A**. Number of reads characterizing the two analyzed honey samples, annotated either directly or by means of the Lowest Common Ancestor (LCA) approach. Data are presented for each sub-dataset (i.e. organism groups defined in the text), considering three different thresholds of sequence identity (<75%, that means without any threshold; ≥ 75%; and ≥ 97%).

| Honey | Groups | Sequence identity < 75% | | | Sequence identity ≥ 75% | | | Sequence identity ≥ 97% | | |
| --- | --- | --- | --- | --- | --- | --- | --- | --- | --- | --- |
|  |  | LCA | Direct | Total | LCA | Direct | Total | LCA | Direct | Total |
| Orange tree blossom honey | Arthropods | 9 | 96 | 105 | 9 | 96 | 105 | 6 | 53 | 59 |
|  | Plants | 162 | 442 | 604 | 162 | 442 | 604 | 162 | 101 | 263 |
|  | Fungi | 58 | 128 | 186 | 58 | 128 | 186 | 16 | 48 | 64 |
|  | Bacteria | 2845 | 17223 | 20068 | 2844 | 17200 | 20044 | 534 | 2390 | 2924 |
|  | Viruses | 1 | 248138 | 248139 | 1 | 248138 | 248139 | 0 | 188360 | 188360 |
|  | Total | 3075 | 266027 | 269102^1^ | 3074 | 266004 | 269078 | 718 | 190952 | 191670 |
| Eucalyptus tree blossom honey | Arthropods | 303 | 2434 | 2737 | 303 | 2431 | 2734 | 256 | 1561 | 1817 |
|  | Plants | 77 | 579 | 656 | 77 | 578 | 655 | 59 | 165 | 224 |
|  | Fungi | 1315 | 1490 | 2805 | 1315 | 1488 | 2803 | 538 | 464 | 1002 |
|  | Bacteria | 18379 | 67205 | 85584 | 18359 | 67074 | 85433 | 10772 | 28004 | 38776 |
|  | Viruses | 6 | 172260 | 172266 | 6 | 172258 | 172264 | 6 | 99534 | 99540 |
|  | Total | 20080 | 243968 | 264048^†^ | 20060 | 243829 | 263889 | 11631 | 129728 | 141359 |

^†^ A few other reads matched sequence entries not classified in the five main organism groups evaluated in this study and they were not considered in the further analyses.

**Table B**. Chao1 richness index (± standard deviation) obtained for the two analysed honeys considering species identified at the 97% identity level and for the five organism groups.

| Honey | Dataset | Chao1 |
| --- | --- | --- |
| Orange tree blossom honey | Arthropods | 9.94 ± 4.36 |
|  | Plants | 186.94 ± 102.47 |
|  | Fungi | 21.82 ± 9.97 |
|  | Bacteria | 209.49 ± 32.26 |
|  | Viruses | 4 ± 2.12 |
| Eucalyptus tree blossom honey | Arthropods | 74.97 ± 34.89 |
|  | Plants | 203.98 ±136.44 |
|  | Fungi | 105.98 ± 43.72 |
|  | Bacteria | 345.53 ± 34.55 |
|  | Viruses | 4 ± 0.5 |

**Table C.** Predominant genera in the two analysed honeys considering the ≥75% sequence identity level based on reads accounting >5% overall reads of the identified organism groups.

| **Honey** | **Groups**^†^ | **Taxid** | **Scientific name** | **Reads** | **% of reads**^‡^ | **‰ of reads_TOT_**^§^ |
| --- | --- | --- | --- | --- | --- | --- |
| **Orange tree blossom honey** | **Arthropods** | 7459 | *Apis* | 55 | 52.38 | 0.20 |
|  |  | 7174 | *Culex* | 8 | 7.62 | 0.03 |
|  |  | 28609 | *Rhagoletis* | 8 | 7.62 | 0.03 |
|  |  | 7028 | *Acyrthosiphon* | 7 | 6.67 | 0.03 |
|  | **Plants** | 2706 | *Citrus* | 191 | 31.62 | 0.71 |
|  |  | 3603 | *Vitis* | 53 | 8.77 | 0.20 |
|  |  | 3877 | *Medicago* | 39 | 6.46 | 0.14 |
|  | **Fungi** | 4953 | *Zygosaccharomyces* | 78 | 41.94 | 0.29 |
|  |  | 5073 | *Penicillium* | 28 | 15.05 | 0.10 |
|  |  | 5529 | *Metarhizium* | 17 | 9.14 | 0.06 |
|  | **Bacteria** | 33882 | *Microbacterium* | 7478 | 37.31 | 27.65 |
|  |  | 374 | *Bradyrhizobium* | 6164 | 30.75 | 22.79 |
|  |  | 1578 | *Lactobacillus* | 2027 | 10.11 | 7.50 |
| **Eucalyptus tree blossom honey** | **Arthropods** | 7459 | *Apis* | 2270 | 82.94 | 8.57 |
|  |  | 7215 | *Drosophila* | 178 | 6.50 | 0.67 |
|  |  | 117248 | *Habropoda* | 162 | 5.92 | 0.61 |
|  | **Plants** | 3932 | *Eucalyptus* | 211 | 32.16 | 0.80 |
|  |  | 36747 | *Orobanche* | 142 | 21.65 | 0.54 |
|  |  | 4685 | *Asparagus* | 56 | 8.54 | 0.21 |
|  | **Fungi** | 4953 | *Zygosaccharomyces* | 1661 | 59.22 | 6.27 |
|  |  | 4895 | *Schizosaccharomyces* | 556 | 19.82 | 2.10 |
|  | **Bacteria** | 1578 | *Lactobacillus* | 38167 | 44.60 | 144.08 |
|  |  | 541 | *Zymomonas* | 11882 | 13.88 | 44.85 |
|  |  | 551 | *Erwinia* | 6154 | 7.19 | 23.23 |

^†^ Viruses are not reported since the ones presenting the genus level have a percentage of reads < 5%.

^‡^ Data are based on the specific number of reads representing the five subsets and the honeys, as reported in Table A (columns “sequence identity <75%).

^§^ Data are based on the total number of annotated reads of that honey, as reported in Table A (columns “sequence identity <75%).

**Table D.** List of species identified in the orange tree blossom honey and number of reads assigned considering the ≥75% identity level (see Table C for explanation of % of reads).

| **Groups** | **Taxid** | **Scientific name - species** | **No. of reads** | **% of reads** | **‰ of reads_TOT_** | **Family** | **Order** |
| --- | --- | --- | --- | --- | --- | --- | --- |
| Arthropods | 7460 | *Apis mellifera* | 41 | 39.048 | 0.152 | Apidae | Hymenoptera |
| Arthropods | 7176 | *Culex quinquefasciatus* | 8 | 7.619 | 0.030 | Culicidae | Diptera |
| Arthropods | 28612 | *Rhagoletis zephyria* | 8 | 7.619 | 0.030 | Tephritidae | Diptera |
| Arthropods | 7029 | *Acyrthosiphon pisum* | 7 | 6.667 | 0.026 | Aphididae | Hemiptera |
| Arthropods | 597456 | *Habropoda laboriosa* | 5 | 4.762 | 0.018 | Apidae | Hymenoptera |
| Arthropods | 7260 | *Drosophila willistoni* | 4 | 3.810 | 0.015 | Drosophilidae | Diptera |
| Arthropods | 7461 | *Apis cerana* | 4 | 3.810 | 0.015 | Apidae | Hymenoptera |
| Arthropods | 7038 | *Bemisia tabaci* | 2 | 1.905 | 0.007 | Aleyrodidae | Hemiptera |
| Arthropods | 7137 | *Galleria mellonella* | 2 | 1.905 | 0.007 | Pyralidae | Lepidoptera |
| Arthropods | 7463 | *Apis florea* | 2 | 1.905 | 0.007 | Apidae | Hymenoptera |
| Arthropods | 1326094 | *Nitidula flavomaculata* | 1 | 0.952 | 0.004 | Nitidulidae | Coleoptera |
| Arthropods | 41112 | *Oryzaephilus surinamensis* | 1 | 0.952 | 0.004 | Silvanidae | Coleoptera |
| Arthropods | 143995 | *Megachile rotundata* | 1 | 0.952 | 0.004 | Megachilidae | Hymenoptera |
| Arthropods | 6945 | *Ixodes scapularis* | 1 | 0.952 | 0.004 | Ixodidae | Ixodida |
| Arthropods | 7070 | *Tribolium castaneum* | 1 | 0.952 | 0.004 | Tenebrionidae | Coleoptera |
| Arthropods | 1535316 | *Aculops pelekassi* | 1 | 0.952 | 0.004 | Eriophyidae | Acarida |
| Arthropods | 75184 | *Bacillus rossius* | 1 | 0.952 | 0.004 | Bacillidae | Phasmatodea |
| Arthropods | 1670774 | *Tydeus sp. F186* | 1 | 0.952 | 0.004 | Tydeidae | Trombidiformes |
| Arthropods | 116153 | *Aethina tumida* | 1 | 0.952 | 0.004 | Nitidulidae | Coleoptera |
| Arthropods | 143948 | *Diuraphis noxia* | 1 | 0.952 | 0.004 | Aphididae | Hemiptera |
| Arthropods | 104421 | *Camponotus floridanus* | 1 | 0.952 | 0.004 | Formicidae | Hymenoptera |
| Arthropods | 7468 | *Apis koschevnikovi* | 1 | 0.952 | 0.004 | Apidae | Hymenoptera |
| Arthropods | 104508 | *Euphydryas editha* | 1 | 0.952 | 0.004 | Nymphalidae | Lepidoptera |
| Plants | 2711 | *Citrus sinensis* | 72 | 11.921 | 0.266 | Rutaceae |  |
| Plants | 29760 | *Vitis vinifera* | 51 | 8.444 | 0.189 | Vitaceae |  |
| Plants | 85681 | *Citrus clementina* | 41 | 6.788 | 0.152 | Rutaceae |  |
| Plants | 3880 | *Medicago truncatula* | 37 | 6.126 | 0.137 | Fabaceae |  |
| Plants | 3847 | *Glycine max* | 16 | 2.649 | 0.059 | Fabaceae |  |
| Plants | 3914 | *Vigna angularis* | 16 | 2.649 | 0.059 | Fabaceae |  |
| Plants | 37690 | *Citrus trifoliata* | 15 | 2.483 | 0.055 | Rutaceae |  |
| Plants | 34305 | *Lotus japonicus* | 14 | 2.318 | 0.052 | Fabaceae |  |
| Plants | 4146 | *Olea europaea* | 10 | 1.656 | 0.037 | Oleaceae |  |
| Plants | 4565 | *Triticum aestivum* | 10 | 1.656 | 0.037 | Poaceae |  |
| Plants | 3827 | *Cicer arietinum* | 9 | 1.490 | 0.033 | Fabaceae |  |
| Plants | 3694 | *Populus trichocarpa* | 9 | 1.490 | 0.033 | Salicaceae |  |
| Plants | 3922 | *Wisteria floribunda* | 6 | 0.993 | 0.022 | Fabaceae |  |
| Plants | 55188 | *Citrus unshiu* | 6 | 0.993 | 0.022 | Rutaceae |  |
| Plants | 4522 | *Lolium perenne* | 6 | 0.993 | 0.022 | Poaceae |  |
| Plants | 3711 | *Brassica rapa* | 4 | 0.662 | 0.015 | Brassicaceae |  |
| Plants | 3821 | *Cajanus cajan* | 4 | 0.662 | 0.015 | Fabaceae |  |
| Plants | 28974 | *Averrhoa carambola* | 4 | 0.662 | 0.015 | Oxalidaceae |  |
| Plants | 2708 | *Citrus limon* | 3 | 0.497 | 0.011 | Rutaceae |  |
| Plants | 299721 | *Sulla coronaria* | 3 | 0.497 | 0.011 | Fabaceae |  |
| Plants | 3505 | *Betula pendula* | 3 | 0.497 | 0.011 | Betulaceae |  |
| Plants | 1641311 | *Caragana kozlowii* | 3 | 0.497 | 0.011 | Fabaceae |  |
| Plants | 3899 | *Trifolium repens* | 3 | 0.497 | 0.011 | Fabaceae |  |
| Plants | 3871 | *Lupinus angustifolius* | 3 | 0.497 | 0.011 | Fabaceae |  |
| Plants | 3726 | *Raphanus sativus* | 3 | 0.497 | 0.011 | Brassicaceae |  |
| Plants | 3906 | *Vicia faba* | 3 | 0.497 | 0.011 | Fabaceae |  |
| Plants | 3067 | *Volvox carteri* | 3 | 0.497 | 0.011 | Volvocaceae |  |
| Plants | 28526 | *Solanum pennellii* | 3 | 0.497 | 0.011 | Solanaceae |  |
| Plants | 15368 | *Brachypodium distachyon* | 2 | 0.331 | 0.007 | Poaceae |  |
| Plants | 4039 | *Daucus carota* | 2 | 0.331 | 0.007 | Apiaceae |  |
| Plants | 4550 | *Secale cereale* | 2 | 0.331 | 0.007 | Poaceae |  |
| Plants | 981085 | *Morus notabilis* | 2 | 0.331 | 0.007 | Moraceae |  |
| Plants | 51503 | *Cynomorium coccineum* | 2 | 0.331 | 0.007 | Cynomoriaceae |  |
| Plants | 38783 | *Avena strigosa* | 2 | 0.331 | 0.007 | Poaceae |  |
| Plants | 126429 | *Hesperelaea palmeri* | 2 | 0.331 | 0.007 | Oleaceae |  |
| Plants | 451732 | *Taverniera cuneifolia* | 2 | 0.331 | 0.007 | Fabaceae |  |
| Plants | 37656 | *Citrus x paradisi* | 2 | 0.331 | 0.007 | Rutaceae |  |
| Plants | 74613 | *Glycyrrhiza uralensis* | 2 | 0.331 | 0.007 | Fabaceae |  |
| Plants | 3885 | *Phaseolus vulgaris* | 2 | 0.331 | 0.007 | Fabaceae |  |
| Plants | 3988 | *Ricinus communis* | 2 | 0.331 | 0.007 | Euphorbiaceae |  |
| Plants | 4577 | *Zea mays* | 1 | 0.166 | 0.004 | Poaceae |  |
| Plants | 75702 | *Populus euphratica* | 1 | 0.166 | 0.004 | Salicaceae |  |
| Plants | 38942 | *Quercus robur* | 1 | 0.166 | 0.004 | Fagaceae |  |
| Plants | 4679 | *Allium cepa* | 1 | 0.166 | 0.004 | Amaryllidaceae |  |
| Plants | 3854 | *Lathyrus aphaca* | 1 | 0.166 | 0.004 | Fabaceae |  |
| Plants | 159030 | *Murraya koenigii* | 1 | 0.166 | 0.004 | Rutaceae |  |
| Plants | 28964 | *Geranium maderense* | 1 | 0.166 | 0.004 | Geraniaceae |  |
| Plants | 2709 | *Citrus cavaleriei* | 1 | 0.166 | 0.004 | Rutaceae |  |
| Plants | 3483 | *Cannabis sativa* | 1 | 0.166 | 0.004 | Cannabaceae |  |
| Plants | 223129 | *Orobanche rapum-genistae* | 1 | 0.166 | 0.004 | Orobanchaceae |  |
| Plants | 4558 | *Sorghum bicolor* | 1 | 0.166 | 0.004 | Poaceae |  |
| Plants | 109171 | *Pachyrhizus erosus* | 1 | 0.166 | 0.004 | Fabaceae |  |
| Plants | 32222 | *Chamaebatiaria millefolium* | 1 | 0.166 | 0.004 | Rosaceae |  |
| Plants | 4498 | *Avena sativa* | 1 | 0.166 | 0.004 | Poaceae |  |
| Plants | 946049 | *Eremosparton songoricum* | 1 | 0.166 | 0.004 | Fabaceae |  |
| Plants | 29730 | *Gossypium raimondii* | 1 | 0.166 | 0.004 | Malvaceae |  |
| Plants | 57918 | *Fragaria vesca* | 1 | 0.166 | 0.004 | Rosaceae |  |
| Plants | 269377 | *Poa palustris* | 1 | 0.166 | 0.004 | Poaceae |  |
| Plants | 539103 | *Indigofera linnaei* | 1 | 0.166 | 0.004 | Fabaceae |  |
| Plants | 3750 | *Malus domestica* | 1 | 0.166 | 0.004 | Rosaceae |  |
| Plants | 4436 | *Batis maritima* | 1 | 0.166 | 0.004 | Bataceae |  |
| Plants | 3900 | *Trifolium subterraneum* | 1 | 0.166 | 0.004 | Fabaceae |  |
| Plants | 185702 | *Apios americana* | 1 | 0.166 | 0.004 | Fabaceae |  |
| Plants | 130453 | *Arachis duranensis* | 1 | 0.166 | 0.004 | Fabaceae |  |
| Plants | 168011 | *Anemone hepatica* | 1 | 0.166 | 0.004 | Ranunculaceae |  |
| Plants | 3860 | *Lathyrus sativus* | 1 | 0.166 | 0.004 | Fabaceae |  |
| Plants | 229543 | *Hibiscus cannabinus* | 1 | 0.166 | 0.004 | Malvaceae |  |
| Plants | 3728 | *Sinapis alba* | 1 | 0.166 | 0.004 | Brassicaceae |  |
| Plants | 326968 | *Ziziphus jujuba* | 1 | 0.166 | 0.004 | Rhamnaceae |  |
| Plants | 4371 | *Drosera regia* | 1 | 0.166 | 0.004 | Droseraceae |  |
| Plants | 3882 | *Onobrychis viciifolia* | 1 | 0.166 | 0.004 | Fabaceae |  |
| Plants | 3888 | *Pisum sativum* | 1 | 0.166 | 0.004 | Fabaceae |  |
| Plants | 4521 | *Lolium multiflorum* | 1 | 0.166 | 0.004 | Poaceae |  |
| Plants | 13451 | *Corylus avellana* | 1 | 0.166 | 0.004 | Betulaceae |  |
| Plants | 47080 | *Glycyrrhiza lepidota* | 1 | 0.166 | 0.004 | Fabaceae |  |
| Plants | 20805 | *Oxytropis lambertii* | 1 | 0.166 | 0.004 | Fabaceae |  |
| Plants | 161934 | *Beta vulgaris* | 1 | 0.166 | 0.004 | Chenopodiaceae |  |
| Plants | 198548 | *Begonia aspleniifolia* | 1 | 0.166 | 0.004 | Begoniaceae |  |
| Plants | 4081 | *Solanum lycopersicum* | 1 | 0.166 | 0.004 | Solanaceae |  |
| Plants | 1857654 | *Castilleja paramensis* | 1 | 0.166 | 0.004 | Orobanchaceae |  |
| Plants | 157791 | *Vigna radiata* | 1 | 0.166 | 0.004 | Fabaceae |  |
| Plants | 85571 | *Citrus reticulata* | 1 | 0.166 | 0.004 | Rutaceae |  |
| Plants | 204362 | *Pinguicula vulgaris* | 1 | 0.166 | 0.004 | Lentibulariaceae |  |
| Plants | 42345 | *Phoenix dactylifera* | 1 | 0.166 | 0.004 | Arecaceae |  |
| Plants | 1573227 | *Heuchera parviflora* | 1 | 0.166 | 0.004 | Saxifragaceae |  |
| Plants | 39329 | *Lavandula angustifolia* | 1 | 0.166 | 0.004 | Lamiaceae |  |
| Plants | 93977 | *Osmanthus fragrans* | 1 | 0.166 | 0.004 | Oleaceae |  |
| Plants | 13894 | *Cocos nucifera* | 1 | 0.166 | 0.004 | Arecaceae |  |
| Plants | 4513 | *Hordeum vulgare* | 1 | 0.166 | 0.004 | Poaceae |  |
| Plants | 56065 | *Millettia pinnata* | 1 | 0.166 | 0.004 | Fabaceae |  |
| Plants | 872936 | *Onobrychis caput-galli* | 1 | 0.166 | 0.004 | Fabaceae |  |
| Plants | 245239 | *Oxalis latifolia* | 1 | 0.166 | 0.004 | Oxalidaceae |  |
| Plants | 37682 | *Aegilops tauschii* | 1 | 0.166 | 0.004 | Poaceae |  |
| Plants | 37334 | *Citrus maxima* | 1 | 0.166 | 0.004 | Rutaceae |  |
| Plants | 130454 | *Arachis ipaensis* | 1 | 0.166 | 0.004 | Fabaceae |  |
| Plants | 223102 | *Orobanche crenata* | 1 | 0.166 | 0.004 | Orobanchaceae |  |
| Plants | 163079 | *Ehretia acuminata* | 1 | 0.166 | 0.004 | Ehretiaceae |  |
| Plants | 3981 | *Hevea brasiliensis* | 1 | 0.166 | 0.004 | Euphorbiaceae |  |
| Fungi | 1108849 | *Penicillium rubens* | 27 | 14.516 | 0.100 | Aspergillaceae |  |
| Fungi | 4956 | *Zygosaccharomyces rouxii* | 19 | 10.215 | 0.070 | Saccharomycetaceae |  |
| Fungi | 500148 | *Metarhizium brunneum* | 15 | 8.065 | 0.055 | Clavicipitaceae |  |
| Fungi | 1365886 | *Zygosaccharomyces parabailii* | 9 | 4.839 | 0.033 | Saccharomycetaceae |  |
| Fungi | 42258 | *Zygosaccharomyces mellis* | 5 | 2.688 | 0.018 | Saccharomycetaceae |  |
| Fungi | 5599 | *Alternaria alternata* | 5 | 2.688 | 0.018 | Pleosporaceae |  |
| Fungi | 307758 | *Ustilago bromivora* | 3 | 1.613 | 0.011 | Ustilaginaceae |  |
| Fungi | 1042127 | *Aureobasidium subglaciale* | 3 | 1.613 | 0.011 | Saccotheciaceae |  |
| Fungi | 655981 | *Pseudogymnoascus destructans* | 3 | 1.613 | 0.011 | Pseudeurotiaceae |  |
| Fungi | 1708541 | *Wallemia mellicola* | 2 | 1.075 | 0.007 | NO_NAME |  |
| Fungi | 40559 | *Botrytis cinerea* | 2 | 1.075 | 0.007 | Sclerotiniaceae |  |
| Fungi | 342668 | *Pseudogymnoascus verrucosus* | 2 | 1.075 | 0.007 | Pseudeurotiaceae |  |
| Fungi | 1802957 | *Cairneyella variabilis* | 2 | 1.075 | 0.007 | Helotiaceae |  |
| Fungi | 300276 | *Lachancea meyersii* | 2 | 1.075 | 0.007 | Saccharomycetaceae |  |
| Fungi | 4959 | *Debaryomyces hansenii* | 2 | 1.075 | 0.007 | Debaryomycetaceae |  |
| Fungi | 559561 | *Aureobasidium namibiae* | 2 | 1.075 | 0.007 | Saccotheciaceae |  |
| Fungi | 1041607 | *Wickerhamomyces ciferrii* | 1 | 0.538 | 0.004 | Phaffomycetaceae |  |
| Fungi | 227369 | *Zygosaccharomyces pseudorouxii* | 1 | 0.538 | 0.004 | Saccharomycetaceae |  |
| Fungi | 28564 | *Talaromyces stipitatus* | 1 | 0.538 | 0.004 | Trichocomaceae |  |
| Fungi | 112493 | *Teratosphaeria molleriana* | 1 | 0.538 | 0.004 | Teratosphaeriaceae |  |
| Fungi | 568076 | *Metarhizium robertsii* | 1 | 0.538 | 0.004 | Clavicipitaceae |  |
| Fungi | 80637 | *Coniophora puteana* | 1 | 0.538 | 0.004 | Coniophoraceae |  |
| Fungi | 1071840 | *Bettsia alvei* | 1 | 0.538 | 0.004 | NO_NAME |  |
| Fungi | 53485 | *Pyrenophora teres* | 1 | 0.538 | 0.004 | Pleosporaceae |  |
| Fungi | 80884 | *Colletotrichum higginsianum* | 1 | 0.538 | 0.004 | Glomerellaceae |  |
| Fungi | 236234 | *Diplodia corticola* | 1 | 0.538 | 0.004 | Botryosphaeriaceae |  |
| Fungi | 393283 | *Pestalotiopsis fici* | 1 | 0.538 | 0.004 | Sporocadaceae |  |
| Fungi | 97096 | *Eutypa lata* | 1 | 0.538 | 0.004 | Diatrypaceae |  |
| Fungi | 280754 | *Pochonia chlamydosporia* | 1 | 0.538 | 0.004 | Clavicipitaceae |  |
| Fungi | 5145 | *Podospora anserina* | 1 | 0.538 | 0.004 | Lasiosphaeriaceae |  |
| Fungi | 60169 | *Penicillium polonicum* | 1 | 0.538 | 0.004 | Aspergillaceae |  |
| Fungi | 1681229 | *Blastomyces gilchristii* | 1 | 0.538 | 0.004 | Ajellomycetaceae |  |
| Fungi | 159246 | *Monascus eremophilus* | 1 | 0.538 | 0.004 | Aspergillaceae |  |
| Fungi | 35629 | *Torulaspora pretoriensis* | 1 | 0.538 | 0.004 | Saccharomycetaceae |  |
| Fungi | 162425 | *Aspergillus nidulans* | 1 | 0.538 | 0.004 | Aspergillaceae |  |
| Fungi | 4934 | *Lachancea kluyveri* | 1 | 0.538 | 0.004 | Saccharomycetaceae |  |
| Fungi | 79858 | *Pseudogymnoascus pannorum* | 1 | 0.538 | 0.004 | Pseudeurotiaceae |  |
| Fungi | 318829 | *Magnaporthe oryzae* | 1 | 0.538 | 0.004 | Magnaporthaceae |  |
| Fungi | 45130 | *Bipolaris sorokiniana* | 1 | 0.538 | 0.004 | Pleosporaceae |  |
| Fungi | 4950 | *Torulaspora delbrueckii* | 1 | 0.538 | 0.004 | Saccharomycetaceae |  |
| Fungi | 698440 | *Marssonina brunnea* | 1 | 0.538 | 0.004 | Dermateaceae |  |
| Fungi | 113608 | *Tetrapisispora phaffii* | 1 | 0.538 | 0.004 | Saccharomycetaceae |  |
| Bacteria | 1906742 | *Microbacterium sp. BH-3-3-3* | 3479 | 17.357 | 12.864 | Microbacteriaceae |  |
| Bacteria | 2033 | *Microbacterium testaceum* | 3075 | 15.341 | 11.370 | Microbacteriaceae |  |
| Bacteria | 148814 | *Lactobacillus kunkeei* | 1989 | 9.923 | 7.355 | Lactobacillaceae |  |
| Bacteria | 1355477 | *Bradyrhizobium diazoefficiens* | 807 | 4.026 | 2.984 | Bradyrhizobiaceae |  |
| Bacteria | 376 | *Bradyrhizobium sp.* | 738 | 3.682 | 2.729 | Bradyrhizobiaceae |  |
| Bacteria | 375 | *Bradyrhizobium japonicum* | 644 | 3.213 | 2.381 | Bradyrhizobiaceae |  |
| Bacteria | 335659 | *Bradyrhizobium sp. S23321* | 642 | 3.203 | 2.374 | Bradyrhizobiaceae |  |
| Bacteria | 549 | *Pantoea agglomerans* | 600 | 2.993 | 2.219 | Erwiniaceae |  |
| Bacteria | 1223566 | *Bradyrhizobium sp. CCGE-LA001* | 387 | 1.931 | 1.431 | Bradyrhizobiaceae |  |
| Bacteria | 317 | *Pseudomonas syringae* | 372 | 1.856 | 1.376 | Pseudomonadaceae |  |
| Bacteria | 1437360 | *Bradyrhizobium erythrophlei* | 270 | 1.347 | 0.998 | Bradyrhizobiaceae |  |
| Bacteria | 1882747 | *Afipia sp. GAS231* | 246 | 1.227 | 0.910 | Bradyrhizobiaceae |  |
| Bacteria | 1076 | *Rhodopseudomonas palustris* | 233 | 1.162 | 0.862 | Bradyrhizobiaceae |  |
| Bacteria | 1274631 | *Bradyrhizobium icense* | 199 | 0.993 | 0.736 | Bradyrhizobiaceae |  |
| Bacteria | 722472 | *Bradyrhizobium lablabi* | 199 | 0.993 | 0.736 | Bradyrhizobiaceae |  |
| Bacteria | 638 | *Arsenophonus nasoniae* | 120 | 0.599 | 0.444 | Morganellaceae |  |
| Bacteria | 1714373 | *Microbacterium sp. No. 7* | 109 | 0.544 | 0.403 | Microbacteriaceae |  |
| Bacteria | 300019 | *Microbacterium paludicola* | 104 | 0.519 | 0.385 | Microbacteriaceae |  |
| Bacteria | 44255 | *Bradyrhizobium oligotrophicum* | 102 | 0.509 | 0.377 | Bradyrhizobiaceae |  |
| Bacteria | 255045 | *Bradyrhizobium canariense* | 96 | 0.479 | 0.355 | Bradyrhizobiaceae |  |
| Bacteria | 115808 | *Bradyrhizobium sp. ORS 285* | 91 | 0.454 | 0.336 | Bradyrhizobiaceae |  |
| Bacteria | 114615 | *Bradyrhizobium sp. ORS 278* | 90 | 0.449 | 0.333 | Bradyrhizobiaceae |  |
| Bacteria | 288000 | *Bradyrhizobium sp. BTAi1* | 82 | 0.409 | 0.303 | Bradyrhizobiaceae |  |
| Bacteria | 84292 | *Microbacterium chocolatum* | 81 | 0.404 | 0.300 | Microbacteriaceae |  |
| Bacteria | 36805 | *Microbacterium aurum* | 78 | 0.389 | 0.288 | Microbacteriaceae |  |
| Bacteria | 912630 | *Microbacterium sp. LKL04* | 66 | 0.329 | 0.244 | Microbacteriaceae |  |
| Bacteria | 2133 | *Spiroplasma citri* | 66 | 0.329 | 0.244 | Spiroplasmataceae |  |
| Bacteria | 370764 | *Microbacterium pygmaeum* | 61 | 0.304 | 0.226 | Microbacteriaceae |  |
| Bacteria | 1510841 | *Parasaccharibacter apium* | 59 | 0.294 | 0.218 | Acetobacteraceae |  |
| Bacteria | 338565 | *Erwinia tasmaniensis* | 55 | 0.274 | 0.203 | Erwiniaceae |  |
| Bacteria | 552 | *Erwinia amylovora* | 52 | 0.259 | 0.192 | Erwiniaceae |  |
| Bacteria | 182337 | *Erwinia billingiae* | 51 | 0.254 | 0.189 | Erwiniaceae |  |
| Bacteria | 367477 | *Microbacterium sp. XT11* | 48 | 0.239 | 0.177 | Microbacteriaceae |  |
| Bacteria | 33069 | *Pseudomonas viridiflava* | 47 | 0.234 | 0.174 | Pseudomonadaceae |  |
| Bacteria | 1938334 | *Microbacterium sp. TPU 3598* | 45 | 0.225 | 0.166 | Microbacteriaceae |  |
| Bacteria | 104336 | *Microbacterium foliorum* | 41 | 0.205 | 0.152 | Microbacteriaceae |  |
| Bacteria | 28447 | *Clavibacter michiganensis* | 39 | 0.195 | 0.144 | Microbacteriaceae |  |
| Bacteria | 453304 | *Agromyces aureus* | 39 | 0.195 | 0.144 | Microbacteriaceae |  |
| Bacteria | 399736 | *Agrococcus jejuensis* | 34 | 0.170 | 0.126 | Microbacteriaceae |  |
| Bacteria | 284016 | *Phenylobacterium zucineum* | 33 | 0.165 | 0.122 | Caulobacteraceae |  |
| Bacteria | 931866 | *Bradyrhizobium ottawaense* | 32 | 0.160 | 0.118 | Bradyrhizobiaceae |  |
| Bacteria | 1575 | *Leifsonia xyli* | 29 | 0.145 | 0.107 | Microbacteriaceae |  |
| Bacteria | 1795053 | *Microbacterium sp. PAMC 28756* | 29 | 0.145 | 0.107 | Microbacteriaceae |  |
| Bacteria | 215689 | *Erwinia sp. Ejp617* | 27 | 0.135 | 0.100 | Erwiniaceae |  |
| Bacteria | 329 | *Ralstonia pickettii* | 24 | 0.120 | 0.089 | Burkholderiaceae |  |
| Bacteria | 199592 | *Microbacterium paraoxydans* | 24 | 0.120 | 0.089 | Microbacteriaceae |  |
| Bacteria | 589382 | *Agromyces flavus* | 23 | 0.115 | 0.085 | Microbacteriaceae |  |
| Bacteria | 150123 | *Plantibacter flavus* | 23 | 0.115 | 0.085 | Microbacteriaceae |  |
| Bacteria | 79967 | *Erwinia pyrifoliae* | 23 | 0.115 | 0.085 | Erwiniaceae |  |
| Bacteria | 912 | *Nitrobacter hamburgensis* | 23 | 0.115 | 0.085 | Bradyrhizobiaceae |  |
| Bacteria | 470934 | *Pantoea vagans* | 22 | 0.110 | 0.081 | Erwiniaceae |  |
| Bacteria | 2137 | *Spiroplasma apis* | 22 | 0.110 | 0.081 | Spiroplasmataceae |  |
| Bacteria | 412690 | *Microterricola viridarii* | 20 | 0.100 | 0.074 | Microbacteriaceae |  |
| Bacteria | 1619308 | *Cnuibacter physcomitrellae* | 19 | 0.095 | 0.070 | Microbacteriaceae |  |
| Bacteria | 615 | *Serratia marcescens* | 18 | 0.090 | 0.067 | Yersiniaceae |  |
| Bacteria | 1980001 | *Cellulosimicrobium sp. TH-20* | 17 | 0.085 | 0.063 | Promicromonosporaceae |  |
| Bacteria | 1561023 | *Curtobacterium sp. MR_MD2014* | 16 | 0.080 | 0.059 | Microbacteriaceae |  |
| Bacteria | 76758 | *Pseudomonas orientalis* | 16 | 0.080 | 0.059 | Pseudomonadaceae |  |
| Bacteria | 1905847 | *Curtobacterium sp. BH-2-1-1* | 15 | 0.075 | 0.055 | Microbacteriaceae |  |
| Bacteria | 550 | *Enterobacter cloacae* | 15 | 0.075 | 0.055 | Enterobacteriaceae |  |
| Bacteria | 1619313 | *Erwinia gerundensis* | 15 | 0.075 | 0.055 | Erwiniaceae |  |
| Bacteria | 366602 | *Caulobacter sp. K31* | 14 | 0.070 | 0.052 | Caulobacteraceae |  |
| Bacteria | 40137 | *Oligotropha carboxidovorans* | 14 | 0.070 | 0.052 | Bradyrhizobiaceae |  |
| Bacteria | 1484157 | *Pantoea sp. PSNIH2* | 13 | 0.065 | 0.048 | Erwiniaceae |  |
| Bacteria | 294 | *Pseudomonas fluorescens* | 13 | 0.065 | 0.048 | Pseudomonadaceae |  |
| Bacteria | 1793722 | *Curtobacterium sp. 9128* | 13 | 0.065 | 0.048 | Microbacteriaceae |  |
| Bacteria | 670052 | *Cryobacterium arcticum* | 13 | 0.065 | 0.048 | Microbacteriaceae |  |
| Bacteria | 1545702 | *Lactobacillus sp. wkB8* | 13 | 0.065 | 0.048 | Lactobacillaceae |  |
| Bacteria | 88688 | *Caulobacter segnis* | 13 | 0.065 | 0.048 | Caulobacteraceae |  |
| Bacteria | 280 | *Xanthobacter autotrophicus* | 12 | 0.060 | 0.044 | Xanthobacteraceae |  |
| Bacteria | 1696072 | *Microbacterium sp. CGR1* | 12 | 0.060 | 0.044 | Microbacteriaceae |  |
| Bacteria | 155892 | *Caulobacter vibrioides* | 11 | 0.055 | 0.041 | Caulobacteraceae |  |
| Bacteria | 717785 | *Hyphomicrobium sp. MC1* | 11 | 0.055 | 0.041 | Hyphomicrobiaceae |  |
| Bacteria | 1938605 | *Brevundimonas sp. LM2* | 11 | 0.055 | 0.041 | Caulobacteraceae |  |
| Bacteria | 1916917 | *Microbacterium sp. 1.5R* | 11 | 0.055 | 0.041 | Microbacteriaceae |  |
| Bacteria | 69395 | *Caulobacter henricii* | 10 | 0.050 | 0.037 | Caulobacteraceae |  |
| Bacteria | 29438 | *Pseudomonas savastanoi* | 10 | 0.050 | 0.037 | Pseudomonadaceae |  |
| Bacteria | 53399 | *Hyphomicrobium denitrificans* | 10 | 0.050 | 0.037 | Hyphomicrobiaceae |  |
| Bacteria | 1267021 | *Frischella perrara* | 10 | 0.050 | 0.037 | Orbaceae |  |
| Bacteria | 674703 | *Rhodoplanes sp. Z2-YC6860* | 10 | 0.050 | 0.037 | Hyphomicrobiaceae |  |
| Bacteria | 33934 | *Anoxybacillus flavithermus* | 10 | 0.050 | 0.037 | Bacillaceae |  |
| Bacteria | 29448 | *Bradyrhizobium elkanii* | 10 | 0.050 | 0.037 | Bradyrhizobiaceae |  |
| Bacteria | 293 | *Brevundimonas diminuta* | 9 | 0.045 | 0.033 | Caulobacteraceae |  |
| Bacteria | 69373 | *Curtobacterium pusillum* | 9 | 0.045 | 0.033 | Microbacteriaceae |  |
| Bacteria | 1686310 | *Bartonella apis* | 9 | 0.045 | 0.033 | Bartonellaceae |  |
| Bacteria | 270351 | *Methylobacterium aquaticum* | 9 | 0.045 | 0.033 | Methylobacteriaceae |  |
| Bacteria | 891974 | *Plautia stali symbiont* | 9 | 0.045 | 0.033 | Enterobacteriaceae |  |
| Bacteria | 158822 | *Cedecea neteri* | 9 | 0.045 | 0.033 | Enterobacteriaceae |  |
| Bacteria | 1196095 | *Gilliamella apicola* | 9 | 0.045 | 0.033 | Orbaceae |  |
| Bacteria | 33888 | *Rathayibacter tritici* | 8 | 0.040 | 0.030 | Microbacteriaceae |  |
| Bacteria | 37927 | *Sinomonas atrocyanea* | 8 | 0.040 | 0.030 | Micrococcaceae |  |
| Bacteria | 1921510 | *Sphingomonas sp. JJ-A5* | 8 | 0.040 | 0.030 | Sphingomonadaceae |  |
| Bacteria | 256701 | *Glutamicibacter arilaitensis* | 8 | 0.040 | 0.030 | Micrococcaceae |  |
| Bacteria | 103817 | *Janibacter terrae* | 8 | 0.040 | 0.030 | Intrasporangiaceae |  |
| Bacteria | 592316 | *Pantoea sp. At-9b* | 8 | 0.040 | 0.030 | Erwiniaceae |  |
| Bacteria | 921 | *Starkeya novella* | 8 | 0.040 | 0.030 | Xanthobacteraceae |  |
| Bacteria | 40324 | *Stenotrophomonas maltophilia* | 8 | 0.040 | 0.030 | Xanthomonadaceae |  |
| Bacteria | 573 | *Klebsiella pneumoniae* | 8 | 0.040 | 0.030 | Enterobacteriaceae |  |
| Bacteria | 1891675 | *Pantoea alhagi* | 8 | 0.040 | 0.030 | Erwiniaceae |  |
| Bacteria | 1526658 | *Bosea vaviloviae* | 8 | 0.040 | 0.030 | Bradyrhizobiaceae |  |
| Bacteria | 562 | *Escherichia coli* | 8 | 0.040 | 0.030 | Enterobacteriaceae |  |
| Bacteria | 408 | *Methylobacterium extorquens* | 7 | 0.035 | 0.026 | Methylobacteriaceae |  |
| Bacteria | 1196083 | *Snodgrassella alvi* | 7 | 0.035 | 0.026 | Neisseriaceae |  |
| Bacteria | 554 | *Pectobacterium carotovorum* | 7 | 0.035 | 0.026 | Pectobacteriaceae |  |
| Bacteria | 1532555 | *Brevundimonas sp. DS20* | 7 | 0.035 | 0.026 | Caulobacteraceae |  |
| Bacteria | 2015316 | *Bosea sp. AS-1* | 7 | 0.035 | 0.026 | Bradyrhizobiaceae |  |
| Bacteria | 82996 | *Serratia plymuthica* | 6 | 0.030 | 0.022 | Yersiniaceae |  |
| Bacteria | 158836 | *Enterobacter hormaechei* | 6 | 0.030 | 0.022 | Enterobacteriaceae |  |
| Bacteria | 1795630 | *Frondihabitans sp. PAMC 28766* | 6 | 0.030 | 0.022 | Microbacteriaceae |  |
| Bacteria | 1583341 | *Pseudomonas cerasi* | 6 | 0.030 | 0.022 | Pseudomonadaceae |  |
| Bacteria | 1076550 | *Pantoea rwandensis* | 6 | 0.030 | 0.022 | Erwiniaceae |  |
| Bacteria | 74313 | *Brevundimonas subvibrioides* | 6 | 0.030 | 0.022 | Caulobacteraceae |  |
| Bacteria | 587753 | *Pseudomonas chlororaphis* | 6 | 0.030 | 0.022 | Pseudomonadaceae |  |
| Bacteria | 381 | *Mesorhizobium loti* | 6 | 0.030 | 0.022 | Phyllobacteriaceae |  |
| Bacteria | 47834 | *Spiroplasma kunkelii* | 6 | 0.030 | 0.022 | Spiroplasmataceae |  |
| Bacteria | 47878 | *Pseudomonas azotoformans* | 6 | 0.030 | 0.022 | Pseudomonadaceae |  |
| Bacteria | 1747 | *Cutibacterium acnes* | 6 | 0.030 | 0.022 | Propionibacteriaceae |  |
| Bacteria | 279828 | *Microcella alkaliphila* | 5 | 0.025 | 0.018 | Microbacteriaceae |  |
| Bacteria | 426117 | *Methylobacterium sp. 4-46* | 5 | 0.025 | 0.018 | Methylobacteriaceae |  |
| Bacteria | 588932 | *Brevundimonas naejangsanensis* | 5 | 0.025 | 0.018 | Caulobacteraceae |  |
| Bacteria | 66269 | *Pantoea stewartii* | 5 | 0.025 | 0.018 | Erwiniaceae |  |
| Bacteria | 139208 | *Isoptericola variabilis* | 5 | 0.025 | 0.018 | Promicromonosporaceae |  |
| Bacteria | 1798223 | *Leifsonia sp. 21MFCrub1.1* | 5 | 0.025 | 0.018 | Microbacteriaceae |  |
| Bacteria | 684552 | *Agrococcus carbonis* | 5 | 0.025 | 0.018 | Microbacteriaceae |  |
| Bacteria | 1711 | *Cellulomonas flavigena* | 5 | 0.025 | 0.018 | Cellulomonadaceae |  |
| Bacteria | 1710 | *Cellulosimicrobium cellulans* | 5 | 0.025 | 0.018 | Promicromonosporaceae |  |
| Bacteria | 497725 | *Kosakonia oryzae* | 5 | 0.025 | 0.018 | Enterobacteriaceae |  |
| Bacteria | 223967 | *Methylobacterium populi* | 5 | 0.025 | 0.018 | Methylobacteriaceae |  |
| Bacteria | 528244 | *Azospirillum thiophilum* | 5 | 0.025 | 0.018 | Rhodospirillaceae |  |
| Bacteria | 33996 | *Gluconacetobacter diazotrophicus* | 5 | 0.025 | 0.018 | Acetobacteraceae |  |
| Bacteria | 399497 | *Tessaracoccus flavescens* | 5 | 0.025 | 0.018 | Propionibacteriaceae |  |
| Bacteria | 384 | *Rhizobium leguminosarum* | 5 | 0.025 | 0.018 | Rhizobiaceae |  |
| Bacteria | 571 | *Klebsiella oxytoca* | 5 | 0.025 | 0.018 | Enterobacteriaceae |  |
| Bacteria | 1861 | *Geodermatophilus obscurus* | 5 | 0.025 | 0.018 | Geodermatophilaceae |  |
| Bacteria | 413497 | *Cronobacter dublinensis* | 5 | 0.025 | 0.018 | Enterobacteriaceae |  |
| Bacteria | 68249 | *Streptomyces pactum* | 5 | 0.025 | 0.018 | Streptomycetaceae |  |
| Bacteria | 913 | *Nitrobacter winogradskyi* | 5 | 0.025 | 0.018 | Bradyrhizobiaceae |  |
| Bacteria | 563 | *Shimwellia blattae* | 5 | 0.025 | 0.018 | Enterobacteriaceae |  |
| Bacteria | 1702325 | *Chelatococcus sp. CO-6* | 4 | 0.020 | 0.015 | Beijerinckiaceae |  |
| Bacteria | 1484158 | *Pantoea sp. PSNIH1* | 4 | 0.020 | 0.015 | Erwiniaceae |  |
| Bacteria | 7 | *Azorhizobium caulinodans* | 4 | 0.020 | 0.015 | Xanthobacteraceae |  |
| Bacteria | 640511 | *Burkholderia sp. CCGE1002* | 4 | 0.020 | 0.015 | Burkholderiaceae |  |
| Bacteria | 2020377 | *Sinomonas sp. R1AF57* | 4 | 0.020 | 0.015 | Micrococcaceae |  |
| Bacteria | 1479019 | *Methylobacterium sp. C1* | 4 | 0.020 | 0.015 | Methylobacteriaceae |  |
| Bacteria | 553 | *Pantoea ananatis* | 4 | 0.020 | 0.015 | Erwiniaceae |  |
| Bacteria | 232721 | *Acidovorax sp. JS42* | 4 | 0.020 | 0.015 | Comamonadaceae |  |
| Bacteria | 216142 | *Pseudomonas rhizosphaerae* | 4 | 0.020 | 0.015 | Pseudomonadaceae |  |
| Bacteria | 1827469 | *Brevundimonas sp. GW460-12-10-14-LB2* | 4 | 0.020 | 0.015 | Caulobacteraceae |  |
| Bacteria | 47917 | *Serratia fonticola* | 4 | 0.020 | 0.015 | Yersiniaceae |  |
| Bacteria | 614 | *Serratia liquefaciens* | 4 | 0.020 | 0.015 | Yersiniaceae |  |
| Bacteria | 655015 | *Methylocystis bryophila* | 4 | 0.020 | 0.015 | Methylocystaceae |  |
| Bacteria | 1166130 | *Enterobacter sp. R4-368* | 4 | 0.020 | 0.015 | Enterobacteriaceae |  |
| Bacteria | 1163710 | *Cronobacter condimenti* | 4 | 0.020 | 0.015 | Enterobacteriaceae |  |
| Bacteria | 546 | *Citrobacter freundii* | 4 | 0.020 | 0.015 | Enterobacteriaceae |  |
| Bacteria | 545 | *Citrobacter koseri* | 4 | 0.020 | 0.015 | Enterobacteriaceae |  |
| Bacteria | 28901 | *Salmonella enterica* | 4 | 0.020 | 0.015 | Enterobacteriaceae |  |
| Bacteria | 208223 | *Kosakonia cowanii* | 4 | 0.020 | 0.015 | Enterobacteriaceae |  |
| Bacteria | 642780 | *Marmoricola scoriae* | 4 | 0.020 | 0.015 | Nocardioidaceae |  |
| Bacteria | 1586287 | *Lentzea guizhouensis* | 4 | 0.020 | 0.015 | Pseudonocardiaceae |  |
| Bacteria | 34073 | *Variovorax paradoxus* | 4 | 0.020 | 0.015 | Comamonadaceae |  |
| Bacteria | 103731 | *Saccharothrix espanaensis* | 4 | 0.020 | 0.015 | Pseudonocardiaceae |  |
| Bacteria | 364410 | *Granulibacter bethesdensis* | 4 | 0.020 | 0.015 | Acetobacteraceae |  |
| Bacteria | 54291 | *Raoultella ornithinolytica* | 4 | 0.020 | 0.015 | Enterobacteriaceae |  |
| Bacteria | 251701 | *Pseudomonas syringae group genomosp. 3* | 4 | 0.020 | 0.015 | Pseudomonadaceae |  |
| Bacteria | 186189 | *Xylanimonas cellulosilytica* | 4 | 0.020 | 0.015 | Promicromonosporaceae |  |
| Bacteria | 1608473 | *Acinetobacter sp. NCu2D-2* | 4 | 0.020 | 0.015 | Moraxellaceae |  |
| Bacteria | 879274 | *Shinella sp. HZN7* | 4 | 0.020 | 0.015 | Rhizobiaceae |  |
| Bacteria | 1792307 | *Bosea sp. PAMC 26642* | 3 | 0.015 | 0.011 | Bradyrhizobiaceae |  |
| Bacteria | 1085 | *Rhodospirillum rubrum* | 3 | 0.015 | 0.011 | Rhodospirillaceae |  |
| Bacteria | 1670800 | *Mesorhizobium sp. B7* | 3 | 0.015 | 0.011 | Phyllobacteriaceae |  |
| Bacteria | 946333 | *Rhizobacter gummiphilus* | 3 | 0.015 | 0.011 | NO_NAME |  |
| Bacteria | 28104 | *Mesorhizobium huakuii* | 3 | 0.015 | 0.011 | Phyllobacteriaceae |  |
| Bacteria | 179636 | *Alicycliphilus denitrificans* | 3 | 0.015 | 0.011 | Comamonadaceae |  |
| Bacteria | 93064 | *Sphingomonas koreensis* | 3 | 0.015 | 0.011 | Sphingomonadaceae |  |
| Bacteria | 935293 | *Yersinia entomophaga* | 3 | 0.015 | 0.011 | Yersiniaceae |  |
| Bacteria | 37919 | *Rhodococcus opacus* | 3 | 0.015 | 0.011 | Nocardiaceae |  |
| Bacteria | 1274 | *Dermacoccus nishinomiyaensis* | 3 | 0.015 | 0.011 | Dermacoccaceae |  |
| Bacteria | 40567 | *Actinosynnema mirum* | 3 | 0.015 | 0.011 | Pseudonocardiaceae |  |
| Bacteria | 266779 | *Chelativorans sp. BNC1* | 3 | 0.015 | 0.011 | Phyllobacteriaceae |  |
| Bacteria | 35703 | *Citrobacter amalonaticus* | 3 | 0.015 | 0.011 | Enterobacteriaceae |  |
| Bacteria | 1798224 | *Jiangella sp. DSM 45060* | 3 | 0.015 | 0.011 | Jiangellaceae |  |
| Bacteria | 34018 | *Rhodospirillum centenum* | 3 | 0.015 | 0.011 | Rhodospirillaceae |  |
| Bacteria | 40214 | *Acinetobacter johnsonii* | 3 | 0.015 | 0.011 | Moraxellaceae |  |
| Bacteria | 114616 | *Methylobacterium nodulans* | 3 | 0.015 | 0.011 | Methylobacteriaceae |  |
| Bacteria | 1089444 | *Dickeya solani* | 3 | 0.015 | 0.011 | Pectobacteriaceae |  |
| Bacteria | 106590 | *Cupriavidus necator* | 3 | 0.015 | 0.011 | Burkholderiaceae |  |
| Bacteria | 1283312 | *Sphingomonas sp. DC-6* | 3 | 0.015 | 0.011 | Sphingomonadaceae |  |
| Bacteria | 348824 | *Rhizobium favelukesii* | 3 | 0.015 | 0.011 | Rhizobiaceae |  |
| Bacteria | 47850 | *Micromonospora aurantiaca* | 3 | 0.015 | 0.011 | Micromonosporaceae |  |
| Bacteria | 105560 | *Methylibium petroleiphilum* | 3 | 0.015 | 0.011 | NO_NAME |  |
| Bacteria | 2751 | *Carnobacterium maltaromaticum* | 3 | 0.015 | 0.011 | Carnobacteriaceae |  |
| Bacteria | 69 | *Lysobacter enzymogenes* | 3 | 0.015 | 0.011 | Xanthomonadaceae |  |
| Bacteria | 1653478 | *Rhodococcus sp. PBTS 1* | 3 | 0.015 | 0.011 | Nocardiaceae |  |
| Bacteria | 263478 | *rice phyllosphere bacterium A* | 3 | 0.015 | 0.011 | NO_NAME |  |
| Bacteria | 444444 | *Chelatococcus daeguensis* | 3 | 0.015 | 0.011 | Beijerinckiaceae |  |
| Bacteria | 413882 | *[Polyangium] brachysporum* | 3 | 0.015 | 0.011 | NO_NAME |  |
| Bacteria | 61645 | *Enterobacter asburiae* | 3 | 0.015 | 0.011 | Enterobacteriaceae |  |
| Bacteria | 108981 | *Acinetobacter schindleri* | 3 | 0.015 | 0.011 | Moraxellaceae |  |
| Bacteria | 1604 | *Lactobacillus amylovorus* | 3 | 0.015 | 0.011 | Lactobacillaceae |  |
| Bacteria | 359 | *Agrobacterium rhizogenes* | 3 | 0.015 | 0.011 | Rhizobiaceae |  |
| Bacteria | 372663 | *Isoptericola dokdonensis* | 3 | 0.015 | 0.011 | Promicromonosporaceae |  |
| Bacteria | 287 | *Pseudomonas aeruginosa* | 3 | 0.015 | 0.011 | Pseudomonadaceae |  |
| Bacteria | 1609977 | *Sphingomonas hengshuiensis* | 3 | 0.015 | 0.011 | Sphingomonadaceae |  |
| Bacteria | 94132 | *Ramlibacter tataouinensis* | 3 | 0.015 | 0.011 | Comamonadaceae |  |
| Bacteria | 419479 | *Jiangella alkaliphila* | 3 | 0.015 | 0.011 | Jiangellaceae |  |
| Bacteria | 1759437 | *Serratia sp. YD25* | 3 | 0.015 | 0.011 | Yersiniaceae |  |
| Bacteria | 158899 | *Collimonas fungivorans* | 3 | 0.015 | 0.011 | Oxalobacteraceae |  |
| Bacteria | 200450 | *Pseudomonas trivialis* | 3 | 0.015 | 0.011 | Pseudomonadaceae |  |
| Bacteria | 569 | *Hafnia alvei* | 3 | 0.015 | 0.011 | Hafniaceae |  |
| Bacteria | 1909395 | *Nonomuraea sp. ATCC 55076* | 3 | 0.015 | 0.011 | Streptosporangiaceae |  |
| Bacteria | 67825 | *Citrobacter rodentium* | 3 | 0.015 | 0.011 | Enterobacteriaceae |  |
| Bacteria | 1148157 | *Acinetobacter oleivorans* | 3 | 0.015 | 0.011 | Moraxellaceae |  |
| Bacteria | 200451 | *Pseudomonas poae* | 3 | 0.015 | 0.011 | Pseudomonadaceae |  |
| Bacteria | 1789224 | *Acinetobacter larvae* | 3 | 0.015 | 0.011 | Moraxellaceae |  |
| Bacteria | 1349819 | *Aureimonas sp. AU20* | 2 | 0.010 | 0.007 | Aurantimonadaceae |  |
| Bacteria | 48296 | *Acinetobacter pittii* | 2 | 0.010 | 0.007 | Moraxellaceae |  |
| Bacteria | 1708 | *Cellulomonas fimi* | 2 | 0.010 | 0.007 | Cellulomonadaceae |  |
| Bacteria | 193 | *Azospirillum lipoferum* | 2 | 0.010 | 0.007 | Rhodospirillaceae |  |
| Bacteria | 192 | *Azospirillum brasilense* | 2 | 0.010 | 0.007 | Rhodospirillaceae |  |
| Bacteria | 83655 | *Leclercia adecarboxylata* | 2 | 0.010 | 0.007 | Enterobacteriaceae |  |
| Bacteria | 644 | *Aeromonas hydrophila* | 2 | 0.010 | 0.007 | Aeromonadaceae |  |
| Bacteria | 1470176 | *Actinoalloteichus hoggarensis* | 2 | 0.010 | 0.007 | Pseudonocardiaceae |  |
| Bacteria | 553510 | *Streptomyces gilvosporeus* | 2 | 0.010 | 0.007 | Streptomycetaceae |  |
| Bacteria | 1114980 | *Spiroplasma atrichopogonis* | 2 | 0.010 | 0.007 | Spiroplasmataceae |  |
| Bacteria | 28141 | *Cronobacter sakazakii* | 2 | 0.010 | 0.007 | Enterobacteriaceae |  |
| Bacteria | 1906741 | *Jeongeupia sp. USM3* | 2 | 0.010 | 0.007 | Chromobacteriaceae |  |
| Bacteria | 2003315 | *Porphyrobacter sp. CACIAM 03H1* | 2 | 0.010 | 0.007 | Erythrobacteraceae |  |
| Bacteria | 60920 | *Sanguibacter keddieii* | 2 | 0.010 | 0.007 | Sanguibacteraceae |  |
| Bacteria | 1804984 | *Burkholderia sp. OLGA172* | 2 | 0.010 | 0.007 | Burkholderiaceae |  |
| Bacteria | 1282 | *Staphylococcus epidermidis* | 2 | 0.010 | 0.007 | Staphylococcaceae |  |
| Bacteria | 745310 | *Sphingomonas sp. MM-1* | 2 | 0.010 | 0.007 | Sphingomonadaceae |  |
| Bacteria | 1546149 | *Diaphorobacter polyhydroxybutyrativorans* | 2 | 0.010 | 0.007 | Comamonadaceae |  |
| Bacteria | 1081039 | *Microbacterium sp. StLB018* | 2 | 0.010 | 0.007 | Microbacteriaceae |  |
| Bacteria | 278153 | *Mesorhizobium sp. WSM1497* | 2 | 0.010 | 0.007 | Phyllobacteriaceae |  |
| Bacteria | 413501 | *Cronobacter muytjensii* | 2 | 0.010 | 0.007 | Enterobacteriaceae |  |
| Bacteria | 348043 | *Streptomyces davawensis* | 2 | 0.010 | 0.007 | Streptomycetaceae |  |
| Bacteria | 1702170 | *Citrobacter sp. FDAARGOS_156* | 2 | 0.010 | 0.007 | Enterobacteriaceae |  |
| Bacteria | 1882682 | *Microvirga ossetica* | 2 | 0.010 | 0.007 | Methylobacteriaceae |  |
| Bacteria | 68895 | *Cupriavidus basilensis* | 2 | 0.010 | 0.007 | Burkholderiaceae |  |
| Bacteria | 654 | *Aeromonas veronii* | 2 | 0.010 | 0.007 | Aeromonadaceae |  |
| Bacteria | 28095 | *Burkholderia gladioli* | 2 | 0.010 | 0.007 | Burkholderiaceae |  |
| Bacteria | 1915 | *Streptomyces lincolnensis* | 2 | 0.010 | 0.007 | Streptomycetaceae |  |
| Bacteria | 11 | *Cellulomonas gilvus* | 2 | 0.010 | 0.007 | Cellulomonadaceae |  |
| Bacteria | 85698 | *Achromobacter xylosoxidans* | 2 | 0.010 | 0.007 | Alcaligenaceae |  |
| Bacteria | 1643450 | *Devosia sp. H5989* | 2 | 0.010 | 0.007 | Hyphomicrobiaceae |  |
| Bacteria | 1218496 | *Lactobacillus sp. Fhon2N* | 2 | 0.010 | 0.007 | Lactobacillaceae |  |
| Bacteria | 29486 | *Yersinia ruckeri* | 2 | 0.010 | 0.007 | Yersiniaceae |  |
| Bacteria | 120107 | *Sphingobium cloacae* | 2 | 0.010 | 0.007 | Sphingomonadaceae |  |
| Bacteria | 161896 | *Corynebacterium camporealensis* | 2 | 0.010 | 0.007 | Corynebacteriaceae |  |
| Bacteria | 1684 | *Bifidobacterium asteroides* | 2 | 0.010 | 0.007 | Bifidobacteriaceae |  |
| Bacteria | 1590 | *Lactobacillus plantarum* | 2 | 0.010 | 0.007 | Lactobacillaceae |  |
| Bacteria | 1294143 | *Pseudomonas sp. ATCC 13867* | 2 | 0.010 | 0.007 | Pseudomonadaceae |  |
| Bacteria | 78527 | *Rhizobium undicola* | 2 | 0.010 | 0.007 | Rhizobiaceae |  |
| Bacteria | 199596 | *Methylocella silvestris* | 2 | 0.010 | 0.007 | Beijerinckiaceae |  |
| Bacteria | 379067 | *Streptomyces bingchenggensis* | 2 | 0.010 | 0.007 | Streptomycetaceae |  |
| Bacteria | 152682 | *Sphingomonas melonis* | 2 | 0.010 | 0.007 | Sphingomonadaceae |  |
| Bacteria | 78587 | *Asticcacaulis excentricus* | 2 | 0.010 | 0.007 | Caulobacteraceae |  |
| Bacteria | 693444 | *Enterobacteriaceae bacterium strain FGI 57* | 2 | 0.010 | 0.007 | Enterobacteriaceae |  |
| Bacteria | 1981173 | *Rhizobium sp. NXC14* | 2 | 0.010 | 0.007 | Rhizobiaceae |  |
| Bacteria | 926 | *Thiomonas intermedia* | 2 | 0.010 | 0.007 | NO_NAME |  |
| Bacteria | 1358 | *Lactococcus lactis* | 2 | 0.010 | 0.007 | Streptococcaceae |  |
| Bacteria | 1549858 | *Sphingomonas taxi* | 2 | 0.010 | 0.007 | Sphingomonadaceae |  |
| Bacteria | 1229727 | *Thiobacimonas profunda* | 2 | 0.010 | 0.007 | Rhodobacteraceae |  |
| Bacteria | 267128 | *Sphingopyxis granuli* | 2 | 0.010 | 0.007 | Sphingomonadaceae |  |
| Bacteria | 204042 | *Dickeya zeae* | 2 | 0.010 | 0.007 | Pectobacteriaceae |  |
| Bacteria | 1814 | *Amycolatopsis methanolica* | 2 | 0.010 | 0.007 | Pseudonocardiaceae |  |
| Bacteria | 465541 | *Streptomyces sp. Mg1* | 2 | 0.010 | 0.007 | Streptomycetaceae |  |
| Bacteria | 61648 | *Kluyvera intermedia* | 2 | 0.010 | 0.007 | Enterobacteriaceae |  |
| Bacteria | 61646 | *Lelliottia amnigena* | 2 | 0.010 | 0.007 | Enterobacteriaceae |  |
| Bacteria | 61647 | *Pluralibacter gergoviae* | 2 | 0.010 | 0.007 | Enterobacteriaceae |  |
| Bacteria | 39645 | *Mesorhizobium ciceri* | 2 | 0.010 | 0.007 | Phyllobacteriaceae |  |
| Bacteria | 34029 | *Leptothrix cholodnii* | 2 | 0.010 | 0.007 | NO_NAME |  |
| Bacteria | 354 | *Azotobacter vinelandii* | 2 | 0.010 | 0.007 | Pseudomonadaceae |  |
| Bacteria | 1663591 | *Magnetospirillum sp. XM-1* | 2 | 0.010 | 0.007 | Rhodospirillaceae |  |
| Bacteria | 1334193 | *Enterobacter lignolyticus* | 2 | 0.010 | 0.007 | Enterobacteriaceae |  |
| Bacteria | 1324350 | *Acinetobacter equi* | 2 | 0.010 | 0.007 | Moraxellaceae |  |
| Bacteria | 339 | *Xanthomonas campestris* | 2 | 0.010 | 0.007 | Xanthomonadaceae |  |
| Bacteria | 53408 | *Pseudomonas citronellolis* | 2 | 0.010 | 0.007 | Pseudomonadaceae |  |
| Bacteria | 29466 | *Veillonella parvula* | 2 | 0.010 | 0.007 | Veillonellaceae |  |
| Bacteria | 1079 | *Blastochloris viridis* | 2 | 0.010 | 0.007 | Hyphomicrobiaceae |  |
| Bacteria | 1646498 | *Acinetobacter sp. TTH0-4* | 2 | 0.010 | 0.007 | Moraxellaceae |  |
| Bacteria | 93944 | *Nonomuraea gerenzanensis* | 2 | 0.010 | 0.007 | Streptosporangiaceae |  |
| Bacteria | 266 | *Paracoccus denitrificans* | 2 | 0.010 | 0.007 | Rhodobacteraceae |  |
| Bacteria | 43669 | *Brachybacterium faecium* | 2 | 0.010 | 0.007 | Dermabacteraceae |  |
| Bacteria | 2020312 | *Rhizobium sp. CIAT894* | 2 | 0.010 | 0.007 | Rhizobiaceae |  |
| Bacteria | 686597 | *Martelella sp. AD-3* | 2 | 0.010 | 0.007 | Aurantimonadaceae |  |
| Bacteria | 48935 | *Novosphingobium aromaticivorans* | 2 | 0.010 | 0.007 | Sphingomonadaceae |  |
| Bacteria | 334852 | *Methylobacterium oryzae* | 2 | 0.010 | 0.007 | Methylobacteriaceae |  |
| Bacteria | 593909 | *Mesorhizobium opportunistum* | 2 | 0.010 | 0.007 | Phyllobacteriaceae |  |
| Bacteria | 399742 | *Enterobacter sp. 638* | 2 | 0.010 | 0.007 | Enterobacteriaceae |  |
| Bacteria | 208962 | *Escherichia albertii* | 2 | 0.010 | 0.007 | Enterobacteriaceae |  |
| Bacteria | 305 | *Ralstonia solanacearum* | 2 | 0.010 | 0.007 | Burkholderiaceae |  |
| Bacteria | 303 | *Pseudomonas putida* | 2 | 0.010 | 0.007 | Pseudomonadaceae |  |
| Bacteria | 38305 | *Corynebacterium vitaeruminis* | 2 | 0.010 | 0.007 | Corynebacteriaceae |  |
| Bacteria | 53336 | *Tatumella citrea* | 2 | 0.010 | 0.007 | Erwiniaceae |  |
| Bacteria | 80867 | *Acidovorax avenae* | 2 | 0.010 | 0.007 | Comamonadaceae |  |
| Bacteria | 80869 | *Acidovorax citrulli* | 2 | 0.010 | 0.007 | Comamonadaceae |  |
| Bacteria | 84595 | *Gordonia polyisoprenivorans* | 2 | 0.010 | 0.007 | Gordoniaceae |  |
| Bacteria | 1136941 | *Gordonia sp. QH-11* | 2 | 0.010 | 0.007 | Gordoniaceae |  |
| Bacteria | 1639108 | *Chania multitudinisentens* | 2 | 0.010 | 0.007 | Yersiniaceae |  |
| Bacteria | 1263550 | *Edwardsiella piscicida* | 2 | 0.010 | 0.007 | Hafniaceae |  |
| Bacteria | 413502 | *Cronobacter turicensis* | 2 | 0.010 | 0.007 | Enterobacteriaceae |  |
| Bacteria | 118060 | *Enterococcus rotai* | 2 | 0.010 | 0.007 | Enterococcaceae |  |
| Bacteria | 535744 | *Cronobacter universalis* | 2 | 0.010 | 0.007 | Enterobacteriaceae |  |
| Bacteria | 283686 | *Kosakonia radicincitans* | 2 | 0.010 | 0.007 | Enterobacteriaceae |  |
| Bacteria | 41276 | *Brevundimonas vesicularis* | 2 | 0.010 | 0.007 | Caulobacteraceae |  |
| Bacteria | 1560345 | *Sphingomonas panacis* | 2 | 0.010 | 0.007 | Sphingomonadaceae |  |
| Bacteria | 1682113 | *Mycobacterium sp. YC-RL4* | 2 | 0.010 | 0.007 | Mycobacteriaceae |  |
| Bacteria | 2001 | *Streptosporangium roseum* | 2 | 0.010 | 0.007 | Streptosporangiaceae |  |
| Bacteria | 488446 | *Burkholderia latens* | 2 | 0.010 | 0.007 | Burkholderiaceae |  |
| Bacteria | 712633 | *Streptococcus sp. oral taxon 431* | 1 | 0.005 | 0.004 | Streptococcaceae |  |
| Bacteria | 1658671 | *Arsenicicoccus sp. oral taxon 190* | 1 | 0.005 | 0.004 | Intrasporangiaceae |  |
| Bacteria | 291112 | *Photorhabdus asymbiotica* | 1 | 0.005 | 0.004 | Morganellaceae |  |
| Bacteria | 216778 | *Stenotrophomonas rhizophila* | 1 | 0.005 | 0.004 | Xanthomonadaceae |  |
| Bacteria | 1118963 | *Arthrobacter sp. Rue61a* | 1 | 0.005 | 0.004 | Micrococcaceae |  |
| Bacteria | 1649596 | *Streptomyces gandocaensis* | 1 | 0.005 | 0.004 | Streptomycetaceae |  |
| Bacteria | 54736 | *Salmonella bongori* | 1 | 0.005 | 0.004 | Enterobacteriaceae |  |
| Bacteria | 84531 | *Lysobacter antibioticus* | 1 | 0.005 | 0.004 | Xanthomonadaceae |  |
| Bacteria | 1678128 | *Limnohabitans sp. 63ED37-2* | 1 | 0.005 | 0.004 | Comamonadaceae |  |
| Bacteria | 1491 | *Clostridium botulinum* | 1 | 0.005 | 0.004 | Clostridiaceae |  |
| Bacteria | 235066 | *Afipia sp. B-91-007287* | 1 | 0.005 | 0.004 | Bradyrhizobiaceae |  |
| Bacteria | 1069 | *Rhodomicrobium vannielii* | 1 | 0.005 | 0.004 | Hyphomicrobiaceae |  |
| Bacteria | 1703 | *Brevibacterium linens* | 1 | 0.005 | 0.004 | Brevibacteriaceae |  |
| Bacteria | 703222 | *Kibdelosporangium sp. MJ126-NF4* | 1 | 0.005 | 0.004 | Pseudonocardiaceae |  |
| Bacteria | 1636152 | *Planctomyces sp. SH-PL62* | 1 | 0.005 | 0.004 | Planctomycetaceae |  |
| Bacteria | 1063 | *Rhodobacter sphaeroides* | 1 | 0.005 | 0.004 | Rhodobacteraceae |  |
| Bacteria | 34038 | *Rahnella aquatilis* | 1 | 0.005 | 0.004 | Yersiniaceae |  |
| Bacteria | 292 | *Burkholderia cepacia* | 1 | 0.005 | 0.004 | Burkholderiaceae |  |
| Bacteria | 43771 | *Corynebacterium urealyticum* | 1 | 0.005 | 0.004 | Corynebacteriaceae |  |
| Bacteria | 33910 | *Amycolatopsis mediterranei* | 1 | 0.005 | 0.004 | Pseudonocardiaceae |  |
| Bacteria | 332056 | *Sphingobium japonicum* | 1 | 0.005 | 0.004 | Sphingomonadaceae |  |
| Bacteria | 69370 | *Microbacterium trichothecenolyticum* | 1 | 0.005 | 0.004 | Microbacteriaceae |  |
| Bacteria | 146939 | *Azospira oryzae* | 1 | 0.005 | 0.004 | Rhodocyclaceae |  |
| Bacteria | 925818 | *Methylobacterium sp. AMS5* | 1 | 0.005 | 0.004 | Methylobacteriaceae |  |
| Bacteria | 33959 | *Lactobacillus johnsonii* | 1 | 0.005 | 0.004 | Lactobacillaceae |  |
| Bacteria | 28037 | *Streptococcus mitis* | 1 | 0.005 | 0.004 | Streptococcaceae |  |
| Bacteria | 117157 | *Actinopolymorpha singaporensis* | 1 | 0.005 | 0.004 | Nocardioidaceae |  |
| Bacteria | 991904 | *Polymorphum gilvum* | 1 | 0.005 | 0.004 | NO_NAME |  |
| Bacteria | 1570939 | *Rhodococcus sp. 2G* | 1 | 0.005 | 0.004 | Nocardiaceae |  |
| Bacteria | 742013 | *Delftia sp. Cs1-4* | 1 | 0.005 | 0.004 | Comamonadaceae |  |
| Bacteria | 948107 | *Paraburkholderia sprentiae* | 1 | 0.005 | 0.004 | Burkholderiaceae |  |
| Bacteria | 716816 | *Oceanicoccus sagamiensis* | 1 | 0.005 | 0.004 | Spongiibacteraceae |  |
| Bacteria | 75612 | *Pseudomonas mandelii* | 1 | 0.005 | 0.004 | Pseudomonadaceae |  |
| Bacteria | 634113 | *Arsenophonus symbiont of Lipoptena fortisetosa* | 1 | 0.005 | 0.004 | Morganellaceae |  |
| Bacteria | 901 | *Desulfovibrio piger* | 1 | 0.005 | 0.004 | Desulfovibrionaceae |  |
| Bacteria | 1245526 | *Pseudomonas guangdongensis* | 1 | 0.005 | 0.004 | Pseudomonadaceae |  |
| Bacteria | 2041 | *Aeromicrobium erythreum* | 1 | 0.005 | 0.004 | Nocardioidaceae |  |
| Bacteria | 296591 | *Polaromonas sp. JS666* | 1 | 0.005 | 0.004 | Comamonadaceae |  |
| Bacteria | 34 | *Myxococcus xanthus* | 1 | 0.005 | 0.004 | Myxococcaceae |  |
| Bacteria | 531844 | *Flavobacteriaceae bacterium 3519-10* | 1 | 0.005 | 0.004 | Flavobacteriaceae |  |
| Bacteria | 438 | *Acetobacter pasteurianus* | 1 | 0.005 | 0.004 | Acetobacteraceae |  |
| Bacteria | 235362 | *Bradyrhizobium sp. CPP* | 1 | 0.005 | 0.004 | Bradyrhizobiaceae |  |
| Bacteria | 219572 | *Pseudomonas antarctica* | 1 | 0.005 | 0.004 | Pseudomonadaceae |  |
| Bacteria | 435 | *Acetobacter aceti* | 1 | 0.005 | 0.004 | Acetobacteraceae |  |
| Bacteria | 2017485 | *Brachybacterium sp. VR2415* | 1 | 0.005 | 0.004 | Dermabacteraceae |  |
| Bacteria | 1407071 | *Acinetobacter sp. TGL-Y2* | 1 | 0.005 | 0.004 | Moraxellaceae |  |
| Bacteria | 330 | *Pseudomonas pseudoalcaligenes* | 1 | 0.005 | 0.004 | Pseudomonadaceae |  |
| Bacteria | 1280052 | *Acinetobacter sp. M131* | 1 | 0.005 | 0.004 | Moraxellaceae |  |
| Bacteria | 190721 | *Ralstonia insidiosa* | 1 | 0.005 | 0.004 | Burkholderiaceae |  |
| Bacteria | 1907 | *Streptomyces glaucescens* | 1 | 0.005 | 0.004 | Streptomycetaceae |  |
| Bacteria | 225992 | *Comamonas kerstersii* | 1 | 0.005 | 0.004 | Comamonadaceae |  |
| Bacteria | 82983 | *Obesumbacterium proteus* | 1 | 0.005 | 0.004 | Hafniaceae |  |
| Bacteria | 1870819 | *Paenibacillus sp. BIHB4019* | 1 | 0.005 | 0.004 | Paenibacillaceae |  |
| Bacteria | 164348 | *Streptomyces puniciscabiei* | 1 | 0.005 | 0.004 | Streptomycetaceae |  |
| Bacteria | 1850250 | *Rhodobacter sp. LPB0142* | 1 | 0.005 | 0.004 | Rhodobacteraceae |  |
| Bacteria | 32002 | *Achromobacter denitrificans* | 1 | 0.005 | 0.004 | Alcaligenaceae |  |
| Bacteria | 1226968 | *Azospirillum humicireducens* | 1 | 0.005 | 0.004 | Rhodospirillaceae |  |
| Bacteria | 244562 | *Bradyrhizobium sp. RD5-C2* | 1 | 0.005 | 0.004 | Bradyrhizobiaceae |  |
| Bacteria | 343 | *Xanthomonas translucens* | 1 | 0.005 | 0.004 | Xanthomonadaceae |  |
| Bacteria | 1115907 | *Bradyrhizobium sp. USDA 4354* | 1 | 0.005 | 0.004 | Bradyrhizobiaceae |  |
| Bacteria | 244366 | *Klebsiella variicola* | 1 | 0.005 | 0.004 | Enterobacteriaceae |  |
| Bacteria | 245188 | *Loktanella vestfoldensis* | 1 | 0.005 | 0.004 | Rhodobacteraceae |  |
| Bacteria | 304895 | *Catenulispora acidiphila* | 1 | 0.005 | 0.004 | Catenulisporaceae |  |
| Bacteria | 81479 | *Rhodoferax antarcticus* | 1 | 0.005 | 0.004 | Comamonadaceae |  |
| Bacteria | 1625 | *Lactobacillus sanfranciscensis* | 1 | 0.005 | 0.004 | Lactobacillaceae |  |
| Bacteria | 1624 | *Lactobacillus salivarius* | 1 | 0.005 | 0.004 | Lactobacillaceae |  |
| Bacteria | 263819 | *Yersinia aleksiciae* | 1 | 0.005 | 0.004 | Yersiniaceae |  |
| Bacteria | 320497 | *Neoasaia chiangmaiensis* | 1 | 0.005 | 0.004 | Acetobacteraceae |  |
| Bacteria | 361575 | *Pseudarthrobacter phenanthrenivorans* | 1 | 0.005 | 0.004 | Micrococcaceae |  |
| Bacteria | 1812935 | *Enterobacter cloacae complex 'Hoffmann cluster IV'* | 1 | 0.005 | 0.004 | Enterobacteriaceae |  |
| Bacteria | 1812934 | *Enterobacter cloacae complex 'Hoffmann cluster III'* | 1 | 0.005 | 0.004 | Enterobacteriaceae |  |
| Bacteria | 312306 | *Pseudomonas entomophila* | 1 | 0.005 | 0.004 | Pseudomonadaceae |  |
| Bacteria | 43948 | *Tolumonas auensis* | 1 | 0.005 | 0.004 | Aeromonadaceae |  |
| Bacteria | 351671 | *Xenorhabdus doucetiae* | 1 | 0.005 | 0.004 | Morganellaceae |  |
| Bacteria | 238 | *Elizabethkingia meningoseptica* | 1 | 0.005 | 0.004 | Flavobacteriaceae |  |
| Bacteria | 401472 | *Corynebacterium ureicelerivorans* | 1 | 0.005 | 0.004 | Corynebacteriaceae |  |
| Bacteria | 298654 | *Frankia inefficax* | 1 | 0.005 | 0.004 | Frankiaceae |  |
| Bacteria | 860235 | *Kibdelosporangium phytohabitans* | 1 | 0.005 | 0.004 | Pseudonocardiaceae |  |
| Bacteria | 71433 | *Mesorhizobium amorphae* | 1 | 0.005 | 0.004 | Phyllobacteriaceae |  |
| Bacteria | 68214 | *Streptomyces griseochromogenes* | 1 | 0.005 | 0.004 | Streptomycetaceae |  |
| Bacteria | 627192 | *Sphingobium sp. SYK-6* | 1 | 0.005 | 0.004 | Sphingomonadaceae |  |
| Bacteria | 1926494 | *Paraburkholderia sp. SOS3* | 1 | 0.005 | 0.004 | Burkholderiaceae |  |
| Bacteria | 95300 | *Pseudomonas vancouverensis* | 1 | 0.005 | 0.004 | Pseudomonadaceae |  |
| Bacteria | 1276 | *Kytococcus sedentarius* | 1 | 0.005 | 0.004 | Dermacoccaceae |  |
| Bacteria | 1134435 | *Thauera humireducens* | 1 | 0.005 | 0.004 | Zoogloeaceae |  |
| Bacteria | 662325 | *Microbacterium sp. EX104* | 1 | 0.005 | 0.004 | Microbacteriaceae |  |
| Bacteria | 728066 | *Pseudarthrobacter equi* | 1 | 0.005 | 0.004 | Micrococcaceae |  |
| Bacteria | 126162 | *secondary endosymbiont of Glycaspis brimblecombei* | 1 | 0.005 | 0.004 | Enterobacteriaceae |  |
| Bacteria | 1280 | *Staphylococcus aureus* | 1 | 0.005 | 0.004 | Staphylococcaceae |  |
| Bacteria | 323415 | *Dokdonella koreensis* | 1 | 0.005 | 0.004 | Rhodanobacteraceae |  |
| Bacteria | 1849491 | *Auricoccus indicus* | 1 | 0.005 | 0.004 | Staphylococcaceae |  |
| Bacteria | 76885 | *Pseudomonas sp. K-62* | 1 | 0.005 | 0.004 | Pseudomonadaceae |  |
| Bacteria | 134537 | *Paraburkholderia fungorum* | 1 | 0.005 | 0.004 | Burkholderiaceae |  |
| Bacteria | 1572644 | *Microbacterium proteolyticum* | 1 | 0.005 | 0.004 | Microbacteriaceae |  |
| Bacteria | 1437453 | *Streptomyces leeuwenhoekii* | 1 | 0.005 | 0.004 | Streptomycetaceae |  |
| Bacteria | 24 | *Shewanella putrefaciens* | 1 | 0.005 | 0.004 | Shewanellaceae |  |
| Bacteria | 1833 | *Rhodococcus erythropolis* | 1 | 0.005 | 0.004 | Nocardiaceae |  |
| Bacteria | 1836 | *Saccharopolyspora erythraea* | 1 | 0.005 | 0.004 | Pseudonocardiaceae |  |
| Bacteria | 758793 | *Burkholderia sp. RPE64* | 1 | 0.005 | 0.004 | Burkholderiaceae |  |
| Bacteria | 2151 | *Mesoplasma florum* | 1 | 0.005 | 0.004 | Entomoplasmataceae |  |
| Bacteria | 471 | *Acinetobacter calcoaceticus* | 1 | 0.005 | 0.004 | Moraxellaceae |  |
| Bacteria | 105219 | *Ralstonia mannitolilytica* | 1 | 0.005 | 0.004 | Burkholderiaceae |  |
| Bacteria | 1030157 | *Sphingomonas sp. KC8* | 1 | 0.005 | 0.004 | Sphingomonadaceae |  |
| Bacteria | 1034889 | *Variovorax sp. HW608* | 1 | 0.005 | 0.004 | Comamonadaceae |  |
| Bacteria | 1842727 | *Rhodoferax sp. DCY110* | 1 | 0.005 | 0.004 | Comamonadaceae |  |
| Bacteria | 43263 | *Pseudomonas alcaligenes* | 1 | 0.005 | 0.004 | Pseudomonadaceae |  |
| Bacteria | 797277 | *Pseudomonas litoralis* | 1 | 0.005 | 0.004 | Pseudomonadaceae |  |
| Bacteria | 377615 | *Thermobacillus composti* | 1 | 0.005 | 0.004 | Paenibacillaceae |  |
| Bacteria | 93222 | *Pandoraea sputorum* | 1 | 0.005 | 0.004 | Burkholderiaceae |  |
| Bacteria | 1335048 | *Defluviimonas alba* | 1 | 0.005 | 0.004 | Rhodobacteraceae |  |
| Bacteria | 1660076 | *Campylobacter sp. RM12175* | 1 | 0.005 | 0.004 | Campylobacteraceae |  |
| Bacteria | 83617 | *Stenotrophomonas nitritireducens* | 1 | 0.005 | 0.004 | Xanthomonadaceae |  |
| Bacteria | 758802 | *Mycobacterium litorale* | 1 | 0.005 | 0.004 | Mycobacteriaceae |  |
| Bacteria | 1390395 | *Sphingomonas sp. LK11* | 1 | 0.005 | 0.004 | Sphingomonadaceae |  |
| Bacteria | 564064 | *Spirosoma rigui* | 1 | 0.005 | 0.004 | Cytophagaceae |  |
| Bacteria | 396 | *Rhizobium phaseoli* | 1 | 0.005 | 0.004 | Rhizobiaceae |  |
| Bacteria | 399 | *Neorhizobium galegae* | 1 | 0.005 | 0.004 | Rhizobiaceae |  |
| Bacteria | 398 | *Rhizobium tropici* | 1 | 0.005 | 0.004 | Rhizobiaceae |  |
| Bacteria | 359110 | *Pseudomonas extremaustralis* | 1 | 0.005 | 0.004 | Pseudomonadaceae |  |
| Bacteria | 29449 | *Rhizobium etli* | 1 | 0.005 | 0.004 | Rhizobiaceae |  |
| Bacteria | 1718 | *Corynebacterium glutamicum* | 1 | 0.005 | 0.004 | Corynebacteriaceae |  |
| Bacteria | 285473 | *Streptomyces rubrolavendulae* | 1 | 0.005 | 0.004 | Streptomycetaceae |  |
| Bacteria | 1862950 | *Rhizobiales bacterium NRL2* | 1 | 0.005 | 0.004 | NO_NAME |  |
| Bacteria | 138336 | *Blastococcus saxobsidens* | 1 | 0.005 | 0.004 | Geodermatophilaceae |  |
| Bacteria | 40215 | *Acinetobacter junii* | 1 | 0.005 | 0.004 | Moraxellaceae |  |
| Bacteria | 648999 | *Micromonospora sp. L5* | 1 | 0.005 | 0.004 | Micromonosporaceae |  |
| Bacteria | 47883 | *Pseudomonas synxantha* | 1 | 0.005 | 0.004 | Pseudomonadaceae |  |
| Bacteria | 1296536 | *Enterobacter xiangfangensis* | 1 | 0.005 | 0.004 | Enterobacteriaceae |  |
| Bacteria | 575406 | *Bradyrhizobium sp. MAFF 211645* | 1 | 0.005 | 0.004 | Bradyrhizobiaceae |  |
| Bacteria | 29430 | *Acinetobacter haemolyticus* | 1 | 0.005 | 0.004 | Moraxellaceae |  |
| Bacteria | 1909732 | *Tessaracoccus sp. T2.5-30* | 1 | 0.005 | 0.004 | Propionibacteriaceae |  |
| Bacteria | 622 | *Shigella dysenteriae* | 1 | 0.005 | 0.004 | Enterobacteriaceae |  |
| Bacteria | 342113 | *Burkholderia oklahomensis* | 1 | 0.005 | 0.004 | Burkholderiaceae |  |
| Bacteria | 624 | *Shigella sonnei* | 1 | 0.005 | 0.004 | Enterobacteriaceae |  |
| Bacteria | 47770 | *Lactobacillus crispatus* | 1 | 0.005 | 0.004 | Lactobacillaceae |  |
| Bacteria | 134676 | *Actinoplanes sp. SE50/110* | 1 | 0.005 | 0.004 | Micromonosporaceae |  |
| Bacteria | 370526 | *Mycobacterium rutilum* | 1 | 0.005 | 0.004 | Mycobacteriaceae |  |
| Bacteria | 28090 | *Acinetobacter lwoffii* | 1 | 0.005 | 0.004 | Moraxellaceae |  |
| Bacteria | 2005046 | *Stenotrophomonas sp. WZN-1* | 1 | 0.005 | 0.004 | Xanthomonadaceae |  |
| Bacteria | 28258 | *Cobetia marina* | 1 | 0.005 | 0.004 | Halomonadaceae |  |
| Bacteria | 1785145 | *Pseudomonas sp. MS586* | 1 | 0.005 | 0.004 | Pseudomonadaceae |  |
| Bacteria | 1502 | *Clostridium perfringens* | 1 | 0.005 | 0.004 | Clostridiaceae |  |
| Bacteria | 651 | *Aeromonas media* | 1 | 0.005 | 0.004 | Aeromonadaceae |  |
| Bacteria | 1500686 | *Pseudomonas sp. Os17* | 1 | 0.005 | 0.004 | Pseudomonadaceae |  |
| Bacteria | 1828 | *Rhodococcus fascians* | 1 | 0.005 | 0.004 | Nocardiaceae |  |
| Bacteria | 211114 | *Allokutzneria albata* | 1 | 0.005 | 0.004 | Pseudonocardiaceae |  |
| Bacteria | 488729 | *Burkholderia metallica* | 1 | 0.005 | 0.004 | Burkholderiaceae |  |
| Bacteria | 61015 | *Staphylococcus succinus* | 1 | 0.005 | 0.004 | Staphylococcaceae |  |
| Bacteria | 248026 | *Cupriavidus pinatubonensis* | 1 | 0.005 | 0.004 | Burkholderiaceae |  |
| Bacteria | 1331258 | *Bordetella pseudohinzii* | 1 | 0.005 | 0.004 | Alcaligenaceae |  |
| Bacteria | 1239307 | *Sodalis praecaptivus* | 1 | 0.005 | 0.004 | Pectobacteriaceae |  |
| Bacteria | 75105 | *Paraburkholderia caribensis* | 1 | 0.005 | 0.004 | Burkholderiaceae |  |
| Bacteria | 1827481 | *Enterobacter sp. ODB01* | 1 | 0.005 | 0.004 | Enterobacteriaceae |  |
| Bacteria | 62977 | *Acinetobacter sp. ADP1* | 1 | 0.005 | 0.004 | Moraxellaceae |  |
| Bacteria | 106592 | *Ensifer adhaerens* | 1 | 0.005 | 0.004 | Rhizobiaceae |  |
| Bacteria | 1963400 | *Bradyrhizobium sp. SM-2017A* | 1 | 0.005 | 0.004 | Bradyrhizobiaceae |  |
| Bacteria | 61652 | *Serratia rubidaea* | 1 | 0.005 | 0.004 | Yersiniaceae |  |
| Bacteria | 53461 | *Nakamurella multipartita* | 1 | 0.005 | 0.004 | Nakamurellaceae |  |
| Bacteria | 1639348 | *Magnetospirillum sp. ME-1* | 1 | 0.005 | 0.004 | Rhodospirillaceae |  |
| Bacteria | 441950 | *Bradyrhizobium iriomotense* | 1 | 0.005 | 0.004 | Bradyrhizobiaceae |  |
| Bacteria | 60552 | *Burkholderia vietnamiensis* | 1 | 0.005 | 0.004 | Burkholderiaceae |  |
| Bacteria | 1843368 | *Sphingobium sp. RAC03* | 1 | 0.005 | 0.004 | Sphingomonadaceae |  |
| Bacteria | 83263 | *Aminobacter aminovorans* | 1 | 0.005 | 0.004 | Phyllobacteriaceae |  |
| Bacteria | 106654 | *Acinetobacter nosocomialis* | 1 | 0.005 | 0.004 | Moraxellaceae |  |
| Bacteria | 418699 | *Azoarcus olearius* | 1 | 0.005 | 0.004 | Zoogloeaceae |  |
| Bacteria | 163275 | *Bradyrhizobium sp. Ppau3-41* | 1 | 0.005 | 0.004 | Bradyrhizobiaceae |  |
| Bacteria | 1879023 | *Mycobacterium sp. djl-10* | 1 | 0.005 | 0.004 | Mycobacteriaceae |  |
| Bacteria | 119219 | *Cupriavidus metallidurans* | 1 | 0.005 | 0.004 | Burkholderiaceae |  |
| Bacteria | 28450 | *Burkholderia pseudomallei* | 1 | 0.005 | 0.004 | Burkholderiaceae |  |
| Bacteria | 76761 | *Pseudomonas veronii* | 1 | 0.005 | 0.004 | Pseudomonadaceae |  |
| Bacteria | 450734 | *Nocardioides dokdonensis* | 1 | 0.005 | 0.004 | Nocardioidaceae |  |
| Bacteria | 47858 | *Micromonospora echinofusca* | 1 | 0.005 | 0.004 | Micromonosporaceae |  |
| Bacteria | 1795874 | *Burkholderia sp. PAMC 28687* | 1 | 0.005 | 0.004 | Burkholderiaceae |  |
| Bacteria | 161895 | *Corynebacterium phocae* | 1 | 0.005 | 0.004 | Corynebacteriaceae |  |
| Bacteria | 86840 | *Pseudomonas cannabina* | 1 | 0.005 | 0.004 | Pseudomonadaceae |  |
| Bacteria | 279113 | *Collimonas pratensis* | 1 | 0.005 | 0.004 | Oxalobacteraceae |  |
| Bacteria | 1503054 | *Burkholderia stagnalis* | 1 | 0.005 | 0.004 | Burkholderiaceae |  |
| Bacteria | 43675 | *Rothia mucilaginosa* | 1 | 0.005 | 0.004 | Micrococcaceae |  |
| Bacteria | 795665 | *Hydrogenophaga sp. PBC* | 1 | 0.005 | 0.004 | Comamonadaceae |  |
| Bacteria | 101564 | *Pseudomonas alcaliphila* | 1 | 0.005 | 0.004 | Pseudomonadaceae |  |
| Bacteria | 1464 | *Paenibacillus larvae* | 1 | 0.005 | 0.004 | Paenibacillaceae |  |
| Bacteria | 129817 | *Pseudomonas brenneri* | 1 | 0.005 | 0.004 | Pseudomonadaceae |  |
| Bacteria | 1882833 | *Frankineae bacterium MT45* | 1 | 0.005 | 0.004 | NO_NAME |  |
| Bacteria | 208224 | *Enterobacter kobei* | 1 | 0.005 | 0.004 | Enterobacteriaceae |  |
| Bacteria | 152480 | *Burkholderia ambifaria* | 1 | 0.005 | 0.004 | Burkholderiaceae |  |
| Bacteria | 1528693 | *Burkholderia sp. AD24* | 1 | 0.005 | 0.004 | Burkholderiaceae |  |
| Bacteria | 482957 | *Burkholderia lata* | 1 | 0.005 | 0.004 | Burkholderiaceae |  |
| Bacteria | 1758689 | *Serinicoccus sp. JLT9* | 1 | 0.005 | 0.004 | Intrasporangiaceae |  |
| Bacteria | 1736691 | *Aeromicrobium choanae* | 1 | 0.005 | 0.004 | Nocardioidaceae |  |
| Bacteria | 1637853 | *Burkholderia sp. NRF60-BP8* | 1 | 0.005 | 0.004 | Burkholderiaceae |  |
| Bacteria | 74318 | *Maricaulis maris* | 1 | 0.005 | 0.004 | Hyphomonadaceae |  |
| Bacteria | 477641 | *Modestobacter marinus* | 1 | 0.005 | 0.004 | Geodermatophilaceae |  |
| Bacteria | 1768242 | *Paucibacter sp. KCTC 42545* | 1 | 0.005 | 0.004 | NO_NAME |  |
| Bacteria | 1404 | *Bacillus megaterium* | 1 | 0.005 | 0.004 | Bacillaceae |  |
| Bacteria | 183795 | *Pseudomonas mediterranea* | 1 | 0.005 | 0.004 | Pseudomonadaceae |  |
| Bacteria | 2066 | *Kitasatospora setae* | 1 | 0.005 | 0.004 | Streptomycetaceae |  |
| Bacteria | 574096 | *Pantoea allii* | 1 | 0.005 | 0.004 | Erwiniaceae |  |
| Bacteria | 794903 | *Opitutaceae bacterium TAV5* | 1 | 0.005 | 0.004 | Opitutaceae |  |
| Bacteria | 316 | *Pseudomonas stutzeri* | 1 | 0.005 | 0.004 | Pseudomonadaceae |  |
| Bacteria | 1926 | *Streptomyces reticuli* | 1 | 0.005 | 0.004 | Streptomycetaceae |  |
| Bacteria | 252970 | *Paraburkholderia phenoliruptrix* | 1 | 0.005 | 0.004 | Burkholderiaceae |  |
| Bacteria | 43357 | *Kutzneria albida* | 1 | 0.005 | 0.004 | Pseudonocardiaceae |  |
| Bacteria | 95486 | *Burkholderia cenocepacia* | 1 | 0.005 | 0.004 | Burkholderiaceae |  |
| Bacteria | 95485 | *Burkholderia stabilis* | 1 | 0.005 | 0.004 | Burkholderiaceae |  |
| Bacteria | 73141 | *Thiocystis violascens* | 1 | 0.005 | 0.004 | Chromatiaceae |  |
| Bacteria | 46677 | *Pseudomonas agarici* | 1 | 0.005 | 0.004 | Pseudomonadaceae |  |
| Bacteria | 1307078 | *Bradyrhizobium sp. CCBAU 43224* | 1 | 0.005 | 0.004 | Bradyrhizobiaceae |  |
| Bacteria | 2021 | *Thermobifida fusca* | 1 | 0.005 | 0.004 | Nocardiopsaceae |  |
| Bacteria | 1685010 | *Chryseobacterium sp. IHB B 10212* | 1 | 0.005 | 0.004 | Flavobacteriaceae |  |
| Bacteria | 1442136 | *Ectothiorhodospira sp. BSL-9* | 1 | 0.005 | 0.004 | Ectothiorhodospiraceae |  |
| Bacteria | 1978566 | *Cryobacterium sp. LW097* | 1 | 0.005 | 0.004 | Microbacteriaceae |  |
| Bacteria | 1771 | *Mycobacterium phlei* | 1 | 0.005 | 0.004 | Mycobacteriaceae |  |
| Bacteria | 1931241 | *Pseudomonas sp. S-6-2* | 1 | 0.005 | 0.004 | Pseudomonadaceae |  |
| Bacteria | 82541 | *Cupriavidus gilardii* | 1 | 0.005 | 0.004 | Burkholderiaceae |  |
| Bacteria | 160386 | *Corynebacterium casei* | 1 | 0.005 | 0.004 | Corynebacteriaceae |  |
| Bacteria | 299146 | *Micromonospora narathiwatensis* | 1 | 0.005 | 0.004 | Micromonosporaceae |  |
| Bacteria | 1416806 | *Bordetella genomosp. 8* | 1 | 0.005 | 0.004 | Alcaligenaceae |  |
| Bacteria | 1136497 | *Brevibacterium siliguriense* | 1 | 0.005 | 0.004 | Brevibacteriaceae |  |
| Bacteria | 1721 | *Corynebacterium callunae* | 1 | 0.005 | 0.004 | Corynebacteriaceae |  |
| Bacteria | 164546 | *Cupriavidus taiwanensis* | 1 | 0.005 | 0.004 | Burkholderiaceae |  |
| Bacteria | 729 | *Haemophilus parainfluenzae* | 1 | 0.005 | 0.004 | Pasteurellaceae |  |
| Bacteria | 37330 | *Nocardia nova* | 1 | 0.005 | 0.004 | Nocardiaceae |  |
| Bacteria | 60520 | *Lactobacillus paraplantarum* | 1 | 0.005 | 0.004 | Lactobacillaceae |  |
| Bacteria | 156978 | *Corynebacterium imitans* | 1 | 0.005 | 0.004 | Corynebacteriaceae |  |
| Bacteria | 156976 | *Corynebacterium riegelii* | 1 | 0.005 | 0.004 | Corynebacteriaceae |  |
| Bacteria | 1440240 | *Bradyrhizobium sp. CCBAU 15618* | 1 | 0.005 | 0.004 | Bradyrhizobiaceae |  |
| Bacteria | 63 | *Vitreoscilla filiformis* | 1 | 0.005 | 0.004 | Neisseriaceae |  |
| Bacteria | 240495 | *Pseudonocardia dioxanivorans* | 1 | 0.005 | 0.004 | Pseudonocardiaceae |  |
| Bacteria | 244563 | *Bradyrhizobium sp. HWK12* | 1 | 0.005 | 0.004 | Bradyrhizobiaceae |  |
| Bacteria | 1581 | *Lactobacillus buchneri* | 1 | 0.005 | 0.004 | Lactobacillaceae |  |
| Bacteria | 76731 | *Roseateles depolymerans* | 1 | 0.005 | 0.004 | NO_NAME |  |
| Bacteria | 118562 | *Arthrospira platensis* | 1 | 0.005 | 0.004 | Microcoleaceae |  |
| Bacteria | 2045 | *Pimelobacter simplex* | 1 | 0.005 | 0.004 | Nocardioidaceae |  |
| Bacteria | 1658672 | *Ottowia sp. oral taxon 894* | 1 | 0.005 | 0.004 | Comamonadaceae |  |
| Bacteria | 1866 | *Actinoplanes missouriensis* | 1 | 0.005 | 0.004 | Micromonosporaceae |  |
| Bacteria | 1637862 | *Burkholderia sp. LA-2-3-30-S1-D2* | 1 | 0.005 | 0.004 | Burkholderiaceae |  |
| Bacteria | 337191 | *Gordonia sp. KTR9* | 1 | 0.005 | 0.004 | Gordoniaceae |  |
| Bacteria | 33 | *Myxococcus fulvus* | 1 | 0.005 | 0.004 | Myxococcaceae |  |
| Bacteria | 529704 | *Frateuria terrea* | 1 | 0.005 | 0.004 | Rhodanobacteraceae |  |
| Bacteria | 1461581 | *Pseudomonas saudimassiliensis* | 1 | 0.005 | 0.004 | Pseudomonadaceae |  |
| Bacteria | 235568 | *Arsenophonus endosymbiont of Australiococcus greville* | 1 | 0.005 | 0.004 | Morganellaceae |  |
| Bacteria | 187491 | *Corynebacterium glaucum* | 1 | 0.005 | 0.004 | Corynebacteriaceae |  |
| Bacteria | 340345 | *Actinoalloteichus hymeniacidonis* | 1 | 0.005 | 0.004 | Pseudonocardiaceae |  |
| Bacteria | 314722 | *Pseudoxanthomonas suwonensis* | 1 | 0.005 | 0.004 | Xanthomonadaceae |  |
| Bacteria | 134962 | *Nocardia soli* | 1 | 0.005 | 0.004 | Nocardiaceae |  |
| Bacteria | 1440194 | *Bradyrhizobium sp. CCBAU 051155* | 1 | 0.005 | 0.004 | Bradyrhizobiaceae |  |
| Bacteria | 1218495 | *Lactobacillus apinorum* | 1 | 0.005 | 0.004 | Lactobacillaceae |  |
| Bacteria | 33050 | *Sphingopyxis macrogoltabida* | 1 | 0.005 | 0.004 | Sphingomonadaceae |  |
| Bacteria | 47885 | *Pseudomonas oryzihabitans* | 1 | 0.005 | 0.004 | Pseudomonadaceae |  |
| Bacteria | 199710 | *Plantibacter agrosticola* | 1 | 0.005 | 0.004 | Microbacteriaceae |  |
| Bacteria | 2746 | *Halomonas elongata* | 1 | 0.005 | 0.004 | Halomonadaceae |  |
| Bacteria | 225991 | *Comamonas aquatica* | 1 | 0.005 | 0.004 | Comamonadaceae |  |
| Bacteria | 675864 | *Auraticoccus monumenti* | 1 | 0.005 | 0.004 | Propionibacteriaceae |  |
| Bacteria | 1675686 | *Sulfurifustis variabilis* | 1 | 0.005 | 0.004 | Acidiferrobacteraceae |  |
| Bacteria | 1028989 | *Pseudomonas sp. StFLB209* | 1 | 0.005 | 0.004 | Pseudomonadaceae |  |
| Bacteria | 55212 | *Erwinia rhapontici* | 1 | 0.005 | 0.004 | Erwiniaceae |  |
| Bacteria | 103816 | *Rhodococcus pyridinivorans* | 1 | 0.005 | 0.004 | Nocardiaceae |  |
| Bacteria | 55211 | *Erwinia persicina* | 1 | 0.005 | 0.004 | Erwiniaceae |  |
| Bacteria | 943830 | *Tardiphaga robiniae* | 1 | 0.005 | 0.004 | Bradyrhizobiaceae |  |
| Bacteria | 575200 | *Corynebacterium maris* | 1 | 0.005 | 0.004 | Corynebacteriaceae |  |
| Bacteria | 261654 | *Micromonospora auratinigra* | 1 | 0.005 | 0.004 | Micromonosporaceae |  |
| Bacteria | 106649 | *Acinetobacter guillouiae* | 1 | 0.005 | 0.004 | Moraxellaceae |  |
| Bacteria | 1588023 | *Arthrobacter sp. Hiyo8* | 1 | 0.005 | 0.004 | Micrococcaceae |  |
| Bacteria | 1070035 | *Bradyrhizobium sp. ARR560* | 1 | 0.005 | 0.004 | Bradyrhizobiaceae |  |
| Bacteria | 358 | *Agrobacterium tumefaciens* | 1 | 0.005 | 0.004 | Rhizobiaceae |  |
| Bacteria | 179270 | *alpha proteobacterium RD5-C2* | 1 | 0.005 | 0.004 | NO_NAME |  |
| Bacteria | 1324352 | *Chryseobacterium gallinarum* | 1 | 0.005 | 0.004 | Flavobacteriaceae |  |
| Bacteria | 1784 | *Mycobacterium simiae* | 1 | 0.005 | 0.004 | Mycobacteriaceae |  |
| Bacteria | 55508 | *Janthinobacterium agaricidamnosum* | 1 | 0.005 | 0.004 | Oxalobacteraceae |  |
| Bacteria | 53407 | *Pseudomonas asplenii* | 1 | 0.005 | 0.004 | Pseudomonadaceae |  |
| Bacteria | 1392877 | *Pseudomonas oryzae* | 1 | 0.005 | 0.004 | Pseudomonadaceae |  |
| Bacteria | 42444 | *Marinovum algicola* | 1 | 0.005 | 0.004 | Rhodobacteraceae |  |
| Bacteria | 1384459 | *Methyloceanibacter caenitepidi* | 1 | 0.005 | 0.004 | NO_NAME |  |
| Bacteria | 502682 | *Erythrobacter gangjinensis* | 1 | 0.005 | 0.004 | Erythrobacteraceae |  |
| Bacteria | 281 | *Xanthobacter flavus* | 1 | 0.005 | 0.004 | Xanthobacteraceae |  |
| Bacteria | 285 | *Comamonas testosteroni* | 1 | 0.005 | 0.004 | Comamonadaceae |  |
| Bacteria | 1704044 | *Arthrobacter sp. ERGS1:01* | 1 | 0.005 | 0.004 | Micrococcaceae |  |
| Bacteria | 67267 | *Streptomyces alboflavus* | 1 | 0.005 | 0.004 | Streptomycetaceae |  |
| Bacteria | 388357 | *Kocuria turfanensis* | 1 | 0.005 | 0.004 | Micrococcaceae |  |
| Bacteria | 53358 | *Intrasporangium calvum* | 1 | 0.005 | 0.004 | Intrasporangiaceae |  |
| Bacteria | 263 | *Francisella tularensis* | 1 | 0.005 | 0.004 | Francisellaceae |  |
| Bacteria | 146923 | *Streptomyces parvulus* | 1 | 0.005 | 0.004 | Streptomycetaceae |  |
| Bacteria | 1623409 | *Bradyrhizobium sp. DGR24* | 1 | 0.005 | 0.004 | Bradyrhizobiaceae |  |
| Bacteria | 1561 | *Clostridium baratii* | 1 | 0.005 | 0.004 | Clostridiaceae |  |
| Bacteria | 257708 | *Roseomonas gilardii* | 1 | 0.005 | 0.004 | Acetobacteraceae |  |
| Bacteria | 38289 | *Corynebacterium jeikeium* | 1 | 0.005 | 0.004 | Corynebacteriaceae |  |
| Bacteria | 1810504 | *Immundisolibacter cernigliae* | 1 | 0.005 | 0.004 | Immundisolibacteraceae |  |
| Bacteria | 180282 | *Delftia tsuruhatensis* | 1 | 0.005 | 0.004 | Comamonadaceae |  |
| Bacteria | 367825 | *Cupriavidus sp. USMAA1020* | 1 | 0.005 | 0.004 | Burkholderiaceae |  |
| Bacteria | 465721 | *Steroidobacter denitrificans* | 1 | 0.005 | 0.004 | Sinobacteraceae |  |
| Bacteria | 418223 | *Methylobacterium phyllosphaerae* | 1 | 0.005 | 0.004 | Methylobacteriaceae |  |
| Bacteria | 1849967 | *Streptomyces sp. SAT1* | 1 | 0.005 | 0.004 | Streptomycetaceae |  |
| Bacteria | 171437 | *Tistrella mobilis* | 1 | 0.005 | 0.004 | Rhodospirillaceae |  |
| Bacteria | 1692238 | *Enterobacter sp. FY-07* | 1 | 0.005 | 0.004 | Enterobacteriaceae |  |
| Bacteria | 499555 | *Dietzia timorensis* | 1 | 0.005 | 0.004 | Dietziaceae |  |
| Bacteria | 1632864 | *Planctomyces sp. SH-PL14* | 1 | 0.005 | 0.004 | Planctomycetaceae |  |
| Bacteria | 986 | *Flavobacterium johnsoniae* | 1 | 0.005 | 0.004 | Flavobacteriaceae |  |
| Bacteria | 1842539 | *Bosea sp. RAC05* | 1 | 0.005 | 0.004 | Bradyrhizobiaceae |  |
| Bacteria | 580165 | *Bacillus cytotoxicus* | 1 | 0.005 | 0.004 | Bacillaceae |  |
| Bacteria | 147645 | *Paracoccus yeei* | 1 | 0.005 | 0.004 | Rhodobacteraceae |  |
| Bacteria | 198620 | *Pseudomonas koreensis* | 1 | 0.005 | 0.004 | Pseudomonadaceae |  |
| Bacteria | 1235591 | *Pseudorhodoplanes sinuspersici* | 1 | 0.005 | 0.004 | NO_NAME |  |
| Bacteria | 217204 | *Achromobacter insolitus* | 1 | 0.005 | 0.004 | Alcaligenaceae |  |
| Bacteria | 1678028 | *Massilia sp. NR 4-1* | 1 | 0.005 | 0.004 | Oxalobacteraceae |  |
| Bacteria | 915 | *Nitrosomonas europaea* | 1 | 0.005 | 0.004 | Nitrosomonadaceae |  |
| Bacteria | 533 | *Beijerinckia indica* | 1 | 0.005 | 0.004 | Beijerinckiaceae |  |
| Bacteria | 1327989 | *Serratia sp. FS14* | 1 | 0.005 | 0.004 | Yersiniaceae |  |
| Bacteria | 1610493 | *Tessaracoccus flavus* | 1 | 0.005 | 0.004 | Propionibacteriaceae |  |
| Bacteria | 35617 | *Mycobacterium indicus pranii* | 1 | 0.005 | 0.004 | Mycobacteriaceae |  |
| Bacteria | 29543 | *Pelobacter propionicus* | 1 | 0.005 | 0.004 | Desulfuromonadaceae |  |
| Bacteria | 1534110 | *Pseudomonas sp. DR 5-09* | 1 | 0.005 | 0.004 | Pseudomonadaceae |  |
| Bacteria | 1930 | *Streptomyces scabiei* | 1 | 0.005 | 0.004 | Streptomycetaceae |  |
| Bacteria | 1938607 | *Sphingomonas sp. LM7* | 1 | 0.005 | 0.004 | Sphingomonadaceae |  |
| Bacteria | 709883 | *Micromonospora zamorensis* | 1 | 0.005 | 0.004 | Micromonosporaceae |  |
| Bacteria | 443602 | *Bradyrhizobium sp. Lop10.4* | 1 | 0.005 | 0.004 | Bradyrhizobiaceae |  |
| Bacteria | 1934254 | *Klebsiella sp. M5al* | 1 | 0.005 | 0.004 | Enterobacteriaceae |  |
| Bacteria | 38302 | *Corynebacterium mycetoides* | 1 | 0.005 | 0.004 | Corynebacteriaceae |  |
| Bacteria | 28151 | *Serratia proteamaculans* | 1 | 0.005 | 0.004 | Yersiniaceae |  |
| Bacteria | 184914 | *Corallococcus coralloides* | 1 | 0.005 | 0.004 | Myxococcaceae |  |
| Bacteria | 1612552 | *Actinoalloteichus sp. ADI127-7* | 1 | 0.005 | 0.004 | Pseudonocardiaceae |  |
| Bacteria | 1230998 | *Corynebacterium frankenforstense* | 1 | 0.005 | 0.004 | Corynebacteriaceae |  |
| Bacteria | 546874 | *Friedmanniella sagamiharensis* | 1 | 0.005 | 0.004 | Nocardioidaceae |  |
| Bacteria | 1983105 | *Lusitaniella coriacea* | 1 | 0.005 | 0.004 | NO_NAME |  |
| Bacteria | 446683 | *Niabella soli* | 1 | 0.005 | 0.004 | Chitinophagaceae |  |
| Bacteria | 1638 | *Listeria ivanovii* | 1 | 0.005 | 0.004 | Listeriaceae |  |
| Bacteria | 121719 | *Pannonibacter phragmitetus* | 1 | 0.005 | 0.004 | Rhodobacteraceae |  |
| Bacteria | 1751294 | *Streptomyces sp. 4F* | 1 | 0.005 | 0.004 | Streptomycetaceae |  |
| Bacteria | 37329 | *Nocardia farcinica* | 1 | 0.005 | 0.004 | Nocardiaceae |  |
| Bacteria | 37326 | *Nocardia brasiliensis* | 1 | 0.005 | 0.004 | Nocardiaceae |  |
| Bacteria | 356851 | *Micromonospora chokoriensis* | 1 | 0.005 | 0.004 | Micromonosporaceae |  |
| Bacteria | 1486991 | *Candidatus Sodalis pierantonius* | 1 | 0.005 | 0.004 | Pectobacteriaceae |  |
| Bacteria | 1297742 | *Myxococcus hansupus* | 1 | 0.005 | 0.004 | Myxococcaceae |  |
| Bacteria | 75588 | *Pseudomonas libanensis* | 1 | 0.005 | 0.004 | Pseudomonadaceae |  |
| Bacteria | 1599 | *Lactobacillus sakei* | 1 | 0.005 | 0.004 | Lactobacillaceae |  |
| Bacteria | 1851395 | *Actinomyces sp. Chiba101* | 1 | 0.005 | 0.004 | Actinomycetaceae |  |
| Bacteria | 47877 | *Pseudomonas amygdali* | 1 | 0.005 | 0.004 | Pseudomonadaceae |  |
| Bacteria | 396595 | *Thioalkalivibrio sp. K90mix* | 1 | 0.005 | 0.004 | Ectothiorhodospiraceae |  |
| Bacteria | 1904640 | *Betaproteobacteria bacterium GR16-43* | 1 | 0.005 | 0.004 | NO_NAME |  |
| Bacteria | 1581680 | *Candidatus Methylopumilus turicensis* | 1 | 0.005 | 0.004 | Methylophilaceae |  |
| Bacteria | 727 | *Haemophilus influenzae* | 1 | 0.005 | 0.004 | Pasteurellaceae |  |
| Bacteria | 1855331 | *Pseudomonas sp. A214* | 1 | 0.005 | 0.004 | Pseudomonadaceae |  |
| Bacteria | 1148509 | *Pseudomonas prosekii* | 1 | 0.005 | 0.004 | Pseudomonadaceae |  |
| Bacteria | 247637 | *marine gamma proteobacterium HTCC2246* | 1 | 0.005 | 0.004 | Halieaceae |  |
| Bacteria | 630 | *Yersinia enterocolitica* | 1 | 0.005 | 0.004 | Yersiniaceae |  |
| Bacteria | 67827 | *Citrobacter werkmanii* | 1 | 0.005 | 0.004 | Enterobacteriaceae |  |
| Bacteria | 246432 | *Staphylococcus equorum* | 1 | 0.005 | 0.004 | Staphylococcaceae |  |
| Bacteria | 1644131 | *Janthinobacterium sp. 1_2014MBL_MicDiv* | 1 | 0.005 | 0.004 | Oxalobacteraceae |  |
| Bacteria | 34062 | *Moraxella osloensis* | 1 | 0.005 | 0.004 | Moraxellaceae |  |
| Bacteria | 169292 | *Corynebacterium aurimucosum* | 1 | 0.005 | 0.004 | Corynebacteriaceae |  |
| Bacteria | 633 | *Yersinia pseudotuberculosis* | 1 | 0.005 | 0.004 | Yersiniaceae |  |
| Bacteria | 358220 | *Acidovorax sp. KKS102* | 1 | 0.005 | 0.004 | Comamonadaceae |  |
| Bacteria | 1894 | *Streptomyces aureofaciens* | 1 | 0.005 | 0.004 | Streptomycetaceae |  |
| Bacteria | 138074 | *Serratia symbiotica* | 1 | 0.005 | 0.004 | Yersiniaceae |  |
| Bacteria | 2134 | *Spiroplasma melliferum* | 1 | 0.005 | 0.004 | Spiroplasmataceae |  |
| Bacteria | 158500 | *Novosphingobium resinovorum* | 1 | 0.005 | 0.004 | Sphingomonadaceae |  |
| Bacteria | 1387353 | *Paludisphaera borealis* | 1 | 0.005 | 0.004 | Isosphaeraceae |  |
| Bacteria | 41 | *Stigmatella aurantiaca* | 1 | 0.005 | 0.004 | Archangiaceae |  |
| Bacteria | 43658 | *Pseudoalteromonas rubra* | 1 | 0.005 | 0.004 | Pseudoalteromonadaceae |  |
| Bacteria | 1112 | *Porphyrobacter neustonensis* | 1 | 0.005 | 0.004 | Erythrobacteraceae |  |
| Bacteria | 1141883 | *Massilia putida* | 1 | 0.005 | 0.004 | Oxalobacteraceae |  |
| Bacteria | 28077 | *Nitrospirillum amazonense* | 1 | 0.005 | 0.004 | Rhodospirillaceae |  |
| Bacteria | 1842537 | *Hydrogenophaga sp. RAC07* | 1 | 0.005 | 0.004 | Comamonadaceae |  |
| Bacteria | 862751 | *Streptomyces sp. SirexAA-E* | 1 | 0.005 | 0.004 | Streptomycetaceae |  |
| Bacteria | 76759 | *Pseudomonas monteilii* | 1 | 0.005 | 0.004 | Pseudomonadaceae |  |
| Bacteria | 460016 | *Bacillus sp. XAL601* | 1 | 0.005 | 0.004 | Bacillaceae |  |
| Bacteria | 38293 | *Photobacterium damselae* | 1 | 0.005 | 0.004 | Vibrionaceae |  |
| Bacteria | 1897729 | *Halomonas aestuarii* | 1 | 0.005 | 0.004 | Halomonadaceae |  |
| Bacteria | 1839800 | *Yersinia sp. FDAARGOS_228* | 1 | 0.005 | 0.004 | Yersiniaceae |  |
| Bacteria | 717822 | *Bradyrhizobium genosp. TUXTLAS-23* | 1 | 0.005 | 0.004 | Bradyrhizobiaceae |  |
| Bacteria | 470 | *Acinetobacter baumannii* | 1 | 0.005 | 0.004 | Moraxellaceae |  |
| Bacteria | 1666906 | *Halomonas sp. HL-93* | 1 | 0.005 | 0.004 | Halomonadaceae |  |
| Bacteria | 152297 | *Pseudoalteromonas issachenkonii* | 1 | 0.005 | 0.004 | Pseudoalteromonadaceae |  |
| Bacteria | 1869170 | *Rhizobium sp. S41* | 1 | 0.005 | 0.004 | Rhizobiaceae |  |
| Virus | 1100043 | *Apis mellifera filamentous virus* | 248133 | 99.998 | 917.519 | NO_NAME |  |
| Virus | 1435008 | *Citrus endogenous pararetrovirus* | 3 | 0.001 | 0.011 | Retroviridae |  |
| Virus | 113366 | *Heliothis virescens ascovirus 3a* | 2 | 0.001 | 0.007 | Ascoviridae |  |
| Virus | 2011074 | Spiroplasma virus SVTS2 | 1 | 0.000 | 0.004 | Inoviridae |  |

**Table E.** List of species identified in the eucalyptus tree blossom honey and number of reads assigned considering the ≥75% identity level (see Table C for explanation of % of reads).

| **Groups** | **Taxid** | **Scientific name - species** | **No. of reads** | **% of reads** | **‰ of reads_TOT_** | **Family** | **Order** |
| --- | --- | --- | --- | --- | --- | --- | --- |
| Arthropods | 7460 | *Apis mellifera* | 1820 | 66.496 | 6.870 | Apidae | Hymenoptera |
| Arthropods | 7461 | *Apis cerana* | 165 | 6.028 | 0.623 | Apidae | Hymenoptera |
| Arthropods | 597456 | *Habropoda laboriosa* | 162 | 5.919 | 0.612 | Apidae | Hymenoptera |
| Arthropods | 29029 | *Drosophila eugracilis* | 67 | 2.448 | 0.253 | Drosophilidae | Diptera |
| Arthropods | 7463 | *Apis florea* | 56 | 2.046 | 0.211 | Apidae | Hymenoptera |
| Arthropods | 7462 | *Apis dorsata* | 55 | 2.009 | 0.208 | Apidae | Hymenoptera |
| Arthropods | 30025 | *Drosophila ficusphila* | 28 | 1.023 | 0.106 | Drosophilidae | Diptera |
| Arthropods | 30023 | *Drosophila elegans* | 27 | 0.986 | 0.102 | Drosophilidae | Diptera |
| Arthropods | 132113 | *Bombus impatiens* | 14 | 0.512 | 0.053 | Apidae | Hymenoptera |
| Arthropods | 30195 | *Bombus terrestris* | 11 | 0.402 | 0.042 | Apidae | Hymenoptera |
| Arthropods | 7137 | *Galleria mellonella* | 9 | 0.329 | 0.034 | Pyralidae | Lepidoptera |
| Arthropods | 28612 | *Rhagoletis zephyria* | 9 | 0.329 | 0.034 | Tephritidae | Diptera |
| Arthropods | 42026 | *Drosophila bipectinata* | 8 | 0.292 | 0.030 | Drosophilidae | Diptera |
| Arthropods | 7227 | *Drosophila melanogaster* | 5 | 0.183 | 0.019 | Drosophilidae | Diptera |
| Arthropods | 29030 | *Drosophila takahashii* | 4 | 0.146 | 0.015 | Drosophilidae | Diptera |
| Arthropods | 7176 | *Culex quinquefasciatus* | 3 | 0.110 | 0.011 | Culicidae | Diptera |
| Arthropods | 156304 | *Ceratina calcarata* | 3 | 0.110 | 0.011 | Apidae | Hymenoptera |
| Arthropods | 7029 | *Acyrthosiphon pisum* | 3 | 0.110 | 0.011 | Aphididae | Hemiptera |
| Arthropods | 516756 | *Eufriesea mexicana* | 2 | 0.073 | 0.008 | Apidae | Hymenoptera |
| Arthropods | 143995 | *Megachile rotundata* | 2 | 0.073 | 0.008 | Megachilidae | Hymenoptera |
| Arthropods | 680683 | *Amyelois transitella* | 2 | 0.073 | 0.008 | Pyralidae | Lepidoptera |
| Arthropods | 1983172 | *Pounamuella complexa* | 2 | 0.073 | 0.008 | Orsolobidae | Araneae |
| Arthropods | 28643 | *Bombus pensylvanicus* | 2 | 0.073 | 0.008 | Apidae | Hymenoptera |
| Arthropods | 30213 | *Vespula pensylvanica* | 2 | 0.073 | 0.008 | Vespidae | Hymenoptera |
| Arthropods | 2015173 | *Ooceraea biroi* | 2 | 0.073 | 0.008 | Formicidae | Hymenoptera |
| Arthropods | 454923 | *Diachasma alloeum* | 2 | 0.073 | 0.008 | Braconidae | Hymenoptera |
| Arthropods | 7274 | *Drosophila serrata* | 2 | 0.073 | 0.008 | Drosophilidae | Diptera |
| Arthropods | 7468 | *Apis koschevnikovi* | 2 | 0.073 | 0.008 | Apidae | Hymenoptera |
| Arthropods | 30019 | *Drosophila busckii* | 1 | 0.037 | 0.004 | Drosophilidae | Diptera |
| Arthropods | 1111430 | *Ramphastobius sp. A PBK-2011* | 1 | 0.037 | 0.004 | Pteronyssidae | Sarcoptiformes |
| Arthropods | 411798 | *Vollenhovia emeryi* | 1 | 0.037 | 0.004 | Formicidae | Hymenoptera |
| Arthropods | 85668 | *Bombus pullatus* | 1 | 0.037 | 0.004 | Apidae | Hymenoptera |
| Arthropods | 7229 | *Drosophila miranda* | 1 | 0.037 | 0.004 | Drosophilidae | Diptera |
| Arthropods | 37344 | *Athalia rosae* | 1 | 0.037 | 0.004 | Tenthredinidae | Hymenoptera |
| Arthropods | 13068 | *Forficula auricularia* | 1 | 0.037 | 0.004 | Forficulidae | Dermaptera |
| Arthropods | 65145 | *Enicospilus ramidulus* | 1 | 0.037 | 0.004 | Ichneumonidae | Hymenoptera |
| Arthropods | 36166 | *Megaselia scalaris* | 1 | 0.037 | 0.004 | Phoridae | Diptera |
| Arthropods | 1504018 | *Andrena (Melandrena) sp. BF-2014* | 1 | 0.037 | 0.004 | Andrenidae | Hymenoptera |
| Arthropods | 294128 | *Hyalella azteca* | 1 | 0.037 | 0.004 | Hyalellidae | Amphipoda |
| Arthropods | 75184 | *Bacillus rossius* | 1 | 0.037 | 0.004 | Bacillidae | Phasmatodea |
| Arthropods | 414922 | *Polistes formosanus* | 1 | 0.037 | 0.004 | Vespidae | Hymenoptera |
| Arthropods | 142686 | *Ceratosolen solmsi* | 1 | 0.037 | 0.004 | Agaonidae | Hymenoptera |
| Arthropods | 159957 | *Thrips imaginis* | 1 | 0.037 | 0.004 | Thripidae | Thysanoptera |
| Arthropods | 144034 | *Pogonomyrmex barbatus* | 1 | 0.037 | 0.004 | Formicidae | Hymenoptera |
| Arthropods | 85660 | *Bombus hortorum* | 1 | 0.037 | 0.004 | Apidae | Hymenoptera |
| Arthropods | 58824 | *Plodia interpunctella* | 1 | 0.037 | 0.004 | Pyralidae | Lepidoptera |
| Arthropods | 109461 | *Varroa destructor* | 1 | 0.037 | 0.004 | Varroidae | Parasitiformes |
| Arthropods | 599974 | *Euglossa iopoecila* | 1 | 0.037 | 0.004 | Apidae | Hymenoptera |
| Arthropods | 599447 | *Peponapis pruinosa* | 1 | 0.037 | 0.004 | Apidae | Hymenoptera |
| Arthropods | 104421 | *Camponotus floridanus* | 1 | 0.037 | 0.004 | Formicidae | Hymenoptera |
| Arthropods | 1712331 | *Erythracarus aff. nasutus ARP-2015* | 1 | 0.037 | 0.004 | Anystidae | Trombidiformes |
| Arthropods | 254370 | *Ctenoplusia agnata* | 1 | 0.037 | 0.004 | Noctuidae | Lepidoptera |
| Arthropods | 856799 | *Proctophyllodes polyxenus* | 1 | 0.037 | 0.004 | Proctophyllodidae | Astigmata |
| Arthropods | 83312 | *Apis nigrocincta* | 1 | 0.037 | 0.004 | Apidae | Hymenoptera |
| Arthropods | 1536863 | *Aculus sp. NJAUEriHN225* | 1 | 0.037 | 0.004 | Eriophyidae | Trombidiformes |
| Arthropods | 7224 | *Drosophila hydei* | 1 | 0.037 | 0.004 | Drosophilidae | Diptera |
| Plants | 71139 | *Eucalyptus grandis* | 185 | 28.201 | 0.698 | Myrtaceae |  |
| Plants | 223129 | *Orobanche rapum-genistae* | 112 | 17.073 | 0.423 | Orobanchaceae |  |
| Plants | 4686 | *Asparagus officinalis* | 55 | 8.384 | 0.208 | Asparagaceae |  |
| Plants | 223102 | *Orobanche crenata* | 30 | 4.573 | 0.113 | Orobanchaceae |  |
| Plants | 3880 | *Medicago truncatula* | 19 | 2.896 | 0.072 | Fabaceae |  |
| Plants | 38942 | *Quercus robur* | 13 | 1.982 | 0.049 | Fagaceae |  |
| Plants | 4530 | *Oryza sativa* | 11 | 1.677 | 0.042 | Poaceae |  |
| Plants | 63459 | *Chenopodium quinoa* | 10 | 1.524 | 0.038 | Chenopodiaceae |  |
| Plants | 3711 | *Brassica rapa* | 7 | 1.067 | 0.026 | Brassicaceae |  |
| Plants | 42345 | *Phoenix dactylifera* | 6 | 0.915 | 0.023 | Arecaceae |  |
| Plants | 29760 | *Vitis vinifera* | 6 | 0.915 | 0.023 | Vitaceae |  |
| Plants | 3694 | *Populus trichocarpa* | 5 | 0.762 | 0.019 | Salicaceae |  |
| Plants | 4039 | *Daucus carota* | 4 | 0.610 | 0.015 | Apiaceae |  |
| Plants | 3641 | *Theobroma cacao* | 4 | 0.610 | 0.015 | Malvaceae |  |
| Plants | 3899 | *Trifolium repens* | 4 | 0.610 | 0.015 | Fabaceae |  |
| Plants | 326968 | *Ziziphus jujuba* | 3 | 0.457 | 0.011 | Rhamnaceae |  |
| Plants | 34305 | *Lotus japonicus* | 3 | 0.457 | 0.011 | Fabaceae |  |
| Plants | 4565 | *Triticum aestivum* | 3 | 0.457 | 0.011 | Poaceae |  |
| Plants | 35608 | *Artemisia annua* | 3 | 0.457 | 0.011 | Asteraceae |  |
| Plants | 3750 | *Malus domestica* | 3 | 0.457 | 0.011 | Rosaceae |  |
| Plants | 72348 | *Artemisia monosperma* | 3 | 0.457 | 0.011 | Asteraceae |  |
| Plants | 3879 | *Medicago sativa* | 3 | 0.457 | 0.011 | Fabaceae |  |
| Plants | 119949 | *Myrtus communis* | 3 | 0.457 | 0.011 | Myrtaceae |  |
| Plants | 3656 | *Cucumis melo* | 2 | 0.305 | 0.008 | Cucurbitaceae |  |
| Plants | 4679 | *Allium cepa* | 2 | 0.305 | 0.008 | Amaryllidaceae |  |
| Plants | 4146 | *Olea europaea* | 2 | 0.305 | 0.008 | Oleaceae |  |
| Plants | 240028 | *Atriplex halimus* | 2 | 0.305 | 0.008 | Chenopodiaceae |  |
| Plants | 4232 | *Helianthus annuus* | 2 | 0.305 | 0.008 | Asteraceae |  |
| Plants | 34316 | *Eucalyptus camaldulensis* | 2 | 0.305 | 0.008 | Myrtaceae |  |
| Plants | 3827 | *Cicer arietinum* | 2 | 0.305 | 0.008 | Fabaceae |  |
| Plants | 92921 | *Silybum marianum* | 2 | 0.305 | 0.008 | Asteraceae |  |
| Plants | 180498 | *Jatropha curcas* | 2 | 0.305 | 0.008 | Euphorbiaceae |  |
| Plants | 4432 | *Nelumbo nucifera* | 2 | 0.305 | 0.008 | Nelumbonaceae |  |
| Plants | 161934 | *Beta vulgaris* | 2 | 0.305 | 0.008 | Chenopodiaceae |  |
| Plants | 4081 | *Solanum lycopersicum* | 2 | 0.305 | 0.008 | Solanaceae |  |
| Plants | 57918 | *Fragaria vesca* | 2 | 0.305 | 0.008 | Rosaceae |  |
| Plants | 41568 | *Chrysanthemum x morifolium* | 2 | 0.305 | 0.008 | Asteraceae |  |
| Plants | 3914 | *Vigna angularis* | 2 | 0.305 | 0.008 | Fabaceae |  |
| Plants | 3708 | *Brassica napus* | 2 | 0.305 | 0.008 | Brassicaceae |  |
| Plants | 4155 | *Erythranthe guttata* | 2 | 0.305 | 0.008 | Phrymaceae |  |
| Plants | 103481 | *Quercus variabilis* | 2 | 0.305 | 0.008 | Fagaceae |  |
| Plants | 35708 | *Arundo donax* | 1 | 0.152 | 0.004 | Poaceae |  |
| Plants | 183840 | *Eucalyptus microtheca* | 1 | 0.152 | 0.004 | Myrtaceae |  |
| Plants | 4577 | *Zea mays* | 1 | 0.152 | 0.004 | Poaceae |  |
| Plants | 1711294 | *Eucalyptus extensa* | 1 | 0.152 | 0.004 | Myrtaceae |  |
| Plants | 87685 | *Eucalyptus spathulata* | 1 | 0.152 | 0.004 | Myrtaceae |  |
| Plants | 107608 | *Amaranthus palmeri* | 1 | 0.152 | 0.004 | Amaranthaceae |  |
| Plants | 51953 | *Elaeis guineensis* | 1 | 0.152 | 0.004 | Arecaceae |  |
| Plants | 28964 | *Geranium maderense* | 1 | 0.152 | 0.004 | Geraniaceae |  |
| Plants | 3483 | *Cannabis sativa* | 1 | 0.152 | 0.004 | Cannabaceae |  |
| Plants | 3933 | *Eucalyptus gunnii* | 1 | 0.152 | 0.004 | Myrtaceae |  |
| Plants | 34317 | *Eucalyptus globulus* | 1 | 0.152 | 0.004 | Myrtaceae |  |
| Plants | 3847 | *Glycine max* | 1 | 0.152 | 0.004 | Fabaceae |  |
| Plants | 75702 | *Populus euphratica* | 1 | 0.152 | 0.004 | Salicaceae |  |
| Plants | 34320 | *Eucalyptus cloeziana* | 1 | 0.152 | 0.004 | Myrtaceae |  |
| Plants | 452569 | *Eucalyptus cladocalyx* | 1 | 0.152 | 0.004 | Myrtaceae |  |
| Plants | 51503 | *Cynomorium coccineum* | 1 | 0.152 | 0.004 | Cynomoriaceae |  |
| Plants | 37682 | *Aegilops tauschii* | 1 | 0.152 | 0.004 | Poaceae |  |
| Plants | 82528 | *Crocus sativus* | 1 | 0.152 | 0.004 | Iridaceae |  |
| Plants | 58331 | *Quercus suber* | 1 | 0.152 | 0.004 | Fagaceae |  |
| Plants | 4436 | *Batis maritima* | 1 | 0.152 | 0.004 | Bataceae |  |
| Plants | 13329 | *Achillea millefolium* | 1 | 0.152 | 0.004 | Asteraceae |  |
| Plants | 50452 | *Arabis alpina* | 1 | 0.152 | 0.004 | Brassicaceae |  |
| Plants | 3654 | *Citrullus lanatus* | 1 | 0.152 | 0.004 | Cucurbitaceae |  |
| Plants | 44015 | *Glycine tomentella* | 1 | 0.152 | 0.004 | Fabaceae |  |
| Plants | 35883 | *Ipomoea nil* | 1 | 0.152 | 0.004 | Convolvulaceae |  |
| Plants | 38727 | *Panicum virgatum* | 1 | 0.152 | 0.004 | Poaceae |  |
| Plants | 60419 | *Castanea mollissima* | 1 | 0.152 | 0.004 | Fagaceae |  |
| Plants | 35935 | *Parthenium argentatum* | 1 | 0.152 | 0.004 | Asteraceae |  |
| Plants | 591214 | *Ferrocalamus rimosivaginus* | 1 | 0.152 | 0.004 | Poaceae |  |
| Plants | 151219 | *Atriplex spongiosa* | 1 | 0.152 | 0.004 | Chenopodiaceae |  |
| Plants | 225215 | *Azorella acaulis* | 1 | 0.152 | 0.004 | Apiaceae |  |
| Plants | 3906 | *Vicia faba* | 1 | 0.152 | 0.004 | Fabaceae |  |
| Plants | 289753 | *Rhus chinensis* | 1 | 0.152 | 0.004 | Anacardiaceae |  |
| Plants | 3505 | *Betula pendula* | 1 | 0.152 | 0.004 | Betulaceae |  |
| Plants | 1573227 | *Heuchera parviflora* | 1 | 0.152 | 0.004 | Saxifragaceae |  |
| Plants | 183858 | *Eucalyptus torquata* | 1 | 0.152 | 0.004 | Myrtaceae |  |
| Plants | 4615 | *Ananas comosus* | 1 | 0.152 | 0.004 | Bromeliaceae |  |
| Plants | 51240 | *Juglans regia* | 1 | 0.152 | 0.004 | Juglandaceae |  |
| Plants | 13894 | *Cocos nucifera* | 1 | 0.152 | 0.004 | Arecaceae |  |
| Plants | 4513 | *Hordeum vulgare* | 1 | 0.152 | 0.004 | Poaceae |  |
| Plants | 79164 | *Daucus aureus* | 1 | 0.152 | 0.004 | Apiaceae |  |
| Plants | 39352 | *Origanum vulgare* | 1 | 0.152 | 0.004 | Lamiaceae |  |
| Plants | 3352 | *Pinus taeda* | 1 | 0.152 | 0.004 | Pinaceae |  |
| Plants | 130454 | *Arachis ipaensis* | 1 | 0.152 | 0.004 | Fabaceae |  |
| Plants | 39511 | *Agave attenuata* | 1 | 0.152 | 0.004 | Asparagaceae |  |
| Plants | 253611 | *Epimedium wushanense* | 1 | 0.152 | 0.004 | Berberidaceae |  |
| Plants | 3981 | *Hevea brasiliensis* | 1 | 0.152 | 0.004 | Euphorbiaceae |  |
| Fungi | 4956 | *Zygosaccharomyces rouxii* | 456 | 16.257 | 1.721 | Saccharomycetaceae |  |
| Fungi | 1365886 | *Zygosaccharomyces parabailii* | 282 | 10.053 | 1.065 | Saccharomycetaceae |  |
| Fungi | 4896 | *Schizosaccharomyces pombe* | 225 | 8.021 | 0.849 | Schizosaccharomycetaceae |  |
| Fungi | 42258 | *Zygosaccharomyces mellis* | 83 | 2.959 | 0.313 | Saccharomycetaceae |  |
| Fungi | 4934 | *Lachancea kluyveri* | 64 | 2.282 | 0.242 | Saccharomycetaceae |  |
| Fungi | 4950 | *Torulaspora delbrueckii* | 58 | 2.068 | 0.219 | Saccharomycetaceae |  |
| Fungi | 35629 | *Torulaspora pretoriensis* | 23 | 0.820 | 0.087 | Saccharomycetaceae |  |
| Fungi | 48254 | *Torulaspora globosa* | 23 | 0.820 | 0.087 | Saccharomycetaceae |  |
| Fungi | 27291 | *Saccharomyces paradoxus* | 17 | 0.606 | 0.064 | Saccharomycetaceae |  |
| Fungi | 4932 | *Saccharomyces cerevisiae* | 16 | 0.570 | 0.060 | Saccharomycetaceae |  |
| Fungi | 27322 | *Metschnikowia bicuspidata* | 15 | 0.535 | 0.057 | Metschnikowiaceae |  |
| Fungi | 300276 | *Lachancea meyersii* | 14 | 0.499 | 0.053 | Saccharomycetaceae |  |
| Fungi | 4927 | *Wickerhamomyces anomalus* | 12 | 0.428 | 0.045 | Phaffomycetaceae |  |
| Fungi | 36033 | *Vanderwaltozyma polyspora* | 11 | 0.392 | 0.042 | Saccharomycetaceae |  |
| Fungi | 227369 | *Zygosaccharomyces pseudorouxii* | 11 | 0.392 | 0.042 | Saccharomycetaceae |  |
| Fungi | 1230905 | *Lachancea mirantina* | 11 | 0.392 | 0.042 | Saccharomycetaceae |  |
| Fungi | 36911 | *Clavispora lusitaniae* | 8 | 0.285 | 0.030 | Metschnikowiaceae |  |
| Fungi | 500148 | *Metarhizium brunneum* | 8 | 0.285 | 0.030 | Clavicipitaceae |  |
| Fungi | 5478 | *[Candida] glabrata* | 6 | 0.214 | 0.023 | Saccharomycetaceae |  |
| Fungi | 1042127 | *Aureobasidium subglaciale* | 6 | 0.214 | 0.023 | Saccotheciaceae |  |
| Fungi | 5599 | *Alternaria alternata* | 6 | 0.214 | 0.023 | Pleosporaceae |  |
| Fungi | 45354 | *[Candida] intermedia* | 6 | 0.214 | 0.023 | Metschnikowiaceae |  |
| Fungi | 381046 | *Lachancea thermotolerans* | 6 | 0.214 | 0.023 | Saccharomycetaceae |  |
| Fungi | 27288 | *Naumovozyma castellii* | 5 | 0.178 | 0.019 | Saccharomycetaceae |  |
| Fungi | 51657 | *Nakaseomyces delphensis* | 5 | 0.178 | 0.019 | Saccharomycetaceae |  |
| Fungi | 1461763 | *Zygosaccharomyces sapae* | 5 | 0.178 | 0.019 | Saccharomycetaceae |  |
| Fungi | 53488 | *Torulaspora franciscae* | 4 | 0.143 | 0.015 | Saccharomycetaceae |  |
| Fungi | 1072105 | *Lachancea dasiensis* | 4 | 0.143 | 0.015 | Saccharomycetaceae |  |
| Fungi | 1071379 | *Tetrapisispora blattae* | 4 | 0.143 | 0.015 | Saccharomycetaceae |  |
| Fungi | 204045 | *Schizosaccharomyces kambucha* | 4 | 0.143 | 0.015 | Schizosaccharomycetaceae |  |
| Fungi | 4929 | *Meyerozyma guilliermondii* | 4 | 0.143 | 0.015 | Debaryomycetaceae |  |
| Fungi | 679534 | *Lachancea nothofagi* | 4 | 0.143 | 0.015 | Saccharomycetaceae |  |
| Fungi | 588726 | *Kazachstania naganishii* | 3 | 0.107 | 0.011 | Saccharomycetaceae |  |
| Fungi | 45286 | *Eremothecium sinecaudum* | 3 | 0.107 | 0.011 | Saccharomycetaceae |  |
| Fungi | 45285 | *Eremothecium cymbalariae* | 3 | 0.107 | 0.011 | Saccharomycetaceae |  |
| Fungi | 1041607 | *Wickerhamomyces ciferrii* | 3 | 0.107 | 0.011 | Phaffomycetaceae |  |
| Fungi | 40302 | *Nosema ceranae* | 2 | 0.071 | 0.008 | Nosematidae |  |
| Fungi | 4903 | *Cyberlindnera jadinii* | 2 | 0.071 | 0.008 | Phaffomycetaceae |  |
| Fungi | 75736 | *Starmerella bombicola* | 2 | 0.071 | 0.008 | NO_NAME |  |
| Fungi | 717740 | *Hyphopichia burtonii* | 2 | 0.071 | 0.008 | Debaryomycetaceae |  |
| Fungi | 27327 | *Metschnikowia reukaufii* | 2 | 0.071 | 0.008 | Metschnikowiaceae |  |
| Fungi | 498019 | *[Candida] auris* | 2 | 0.071 | 0.008 | Metschnikowiaceae |  |
| Fungi | 1071840 | *Bettsia alvei* | 2 | 0.071 | 0.008 | NO_NAME |  |
| Fungi | 4920 | *Millerozyma farinosa* | 2 | 0.071 | 0.008 | Debaryomycetaceae |  |
| Fungi | 39397 | *Candida sake* | 2 | 0.071 | 0.008 | NO_NAME |  |
| Fungi | 114497 | *Isaria fumosorosea* | 2 | 0.071 | 0.008 | Cordycipitaceae |  |
| Fungi | 706196 | *Saccharomyces arboricola* | 2 | 0.071 | 0.008 | Saccharomycetaceae |  |
| Fungi | 4911 | *Kluyveromyces marxianus* | 2 | 0.071 | 0.008 | Saccharomycetaceae |  |
| Fungi | 1191714 | *Schizosaccharomyces sp. UFLA CHYE5.39* | 1 | 0.036 | 0.004 | Schizosaccharomycetaceae |  |
| Fungi | 384582 | *Zygosaccharomyces sp. G70* | 1 | 0.036 | 0.004 | Saccharomycetaceae |  |
| Fungi | 340170 | *Spathaspora passalidarum* | 1 | 0.036 | 0.004 | Debaryomycetaceae |  |
| Fungi | 1505670 | *Puccinia cf. psidii AE-2014* | 1 | 0.036 | 0.004 | Pucciniaceae |  |
| Fungi | 27289 | *Naumovozyma dairenensis* | 1 | 0.036 | 0.004 | Saccharomycetaceae |  |
| Fungi | 76773 | *Malassezia globosa* | 1 | 0.036 | 0.004 | Malasseziaceae |  |
| Fungi | 353897 | *Metschnikowia aberdeeniae* | 1 | 0.036 | 0.004 | Metschnikowiaceae |  |
| Fungi | 58627 | *Debaryomyces fabryi* | 1 | 0.036 | 0.004 | Debaryomycetaceae |  |
| Fungi | 28985 | *Kluyveromyces lactis* | 1 | 0.036 | 0.004 | Saccharomycetaceae |  |
| Fungi | 576137 | *Phialocephala subalpina* | 1 | 0.036 | 0.004 | NO_NAME |  |
| Fungi | 1090491 | *Zygosaccharomyces sp. NCAIM Y.01994* | 1 | 0.036 | 0.004 | Saccharomycetaceae |  |
| Fungi | 13684 | *Parastagonospora nodorum* | 1 | 0.036 | 0.004 | Phaeosphaeriaceae |  |
| Fungi | 45130 | *Bipolaris sorokiniana* | 1 | 0.036 | 0.004 | Pleosporaceae |  |
| Fungi | 29908 | *Sporothrix schenckii* | 1 | 0.036 | 0.004 | Ophiostomataceae |  |
| Fungi | 1708541 | *Wallemia mellicola* | 1 | 0.036 | 0.004 | NO_NAME |  |
| Fungi | 5016 | *Bipolaris maydis* | 1 | 0.036 | 0.004 | Pleosporaceae |  |
| Fungi | 45517 | *Metschnikowia gruessii* | 1 | 0.036 | 0.004 | Metschnikowiaceae |  |
| Fungi | 1685470 | *Metschnikowia sp. NJ-2015* | 1 | 0.036 | 0.004 | Metschnikowiaceae |  |
| Fungi | 1108849 | *Penicillium rubens* | 1 | 0.036 | 0.004 | Aspergillaceae |  |
| Fungi | 36907 | *Kazachstania telluris* | 1 | 0.036 | 0.004 | Saccharomycetaceae |  |
| Fungi | 391826 | *Candida corydali* | 1 | 0.036 | 0.004 | Debaryomycetaceae |  |
| Fungi | 51914 | *[Candida] castellii* | 1 | 0.036 | 0.004 | Saccharomycetaceae |  |
| Fungi | 391825 | *Candida chauliodis* | 1 | 0.036 | 0.004 | Debaryomycetaceae |  |
| Fungi | 5480 | *Candida parapsilosis* | 1 | 0.036 | 0.004 | Debaryomycetaceae |  |
| Fungi | 1432397 | *Metschnikowia sp. UWOPS 03-147.1* | 1 | 0.036 | 0.004 | Metschnikowiaceae |  |
| Fungi | 36038 | *Barnettozyma californica* | 1 | 0.036 | 0.004 | Phaffomycetaceae |  |
| Fungi | 559561 | *Aureobasidium namibiae* | 1 | 0.036 | 0.004 | Saccotheciaceae |  |
| Fungi | 4957 | *Zygosaccharomyces bisporus* | 1 | 0.036 | 0.004 | Saccharomycetaceae |  |
| Fungi | 4954 | *Zygosaccharomyces bailii* | 1 | 0.036 | 0.004 | Saccharomycetaceae |  |
| Fungi | 113608 | *Tetrapisispora phaffii* | 1 | 0.036 | 0.004 | Saccharomycetaceae |  |
| Fungi | 73501 | *Cordyceps militaris* | 1 | 0.036 | 0.004 | Cordycipitaceae |  |
| Fungi | 566037 | *Saccharomycetaceae sp. 'Ashbya aceri'* | 1 | 0.036 | 0.004 | Saccharomycetaceae |  |
| Fungi | 5482 | *Candida tropicalis* | 1 | 0.036 | 0.004 | Debaryomycetaceae |  |
| Fungi | 29833 | *Hanseniaspora uvarum* | 1 | 0.036 | 0.004 | Saccharomycodaceae |  |
| Fungi | 104416 | *Filobasidium wieringae* | 1 | 0.036 | 0.004 | Filobasidiaceae |  |
| Fungi | 176275 | *Beauveria bassiana* | 1 | 0.036 | 0.004 | Cordycipitaceae |  |
| Fungi | 169388 | *Fusarium solani* | 1 | 0.036 | 0.004 | Nectriaceae |  |
| Fungi | 335852 | *Arthrinium arundinis* | 1 | 0.036 | 0.004 | Apiosporaceae |  |
| Fungi | 29898 | *Rhodotorula graminis* | 1 | 0.036 | 0.004 | Sporidiobolaceae |  |
| Fungi | 36022 | *Cyberlindnera fabianii* | 1 | 0.036 | 0.004 | Phaffomycetaceae |  |
| Fungi | 1134516 | *Metschnikowia sp. Y-37* | 1 | 0.036 | 0.004 | Metschnikowiaceae |  |
| Fungi | 140110 | *Nectria haematococca* | 1 | 0.036 | 0.004 | Nectriaceae |  |
| Fungi | 63577 | *Trichoderma atroviride* | 1 | 0.036 | 0.004 | Hypocreaceae |  |
| Fungi | 128442 | *Talaromyces pinophilus* | 1 | 0.036 | 0.004 | Trichocomaceae |  |
| Fungi | 1725355 | *Saccharomycopsis fibuligera x Saccharomycopsis cf. fibuligera* | 1 | 0.036 | 0.004 | Saccharomycopsidaceae |  |
| Fungi | 220672 | *Leptosphaeria biglobosa* | 1 | 0.036 | 0.004 | Leptosphaeriaceae |  |
| Fungi | 1610689 | *Sarocladium implicatum* | 1 | 0.036 | 0.004 | NO_NAME |  |
| Fungi | 4924 | *Scheffersomyces stipitis* | 1 | 0.036 | 0.004 | Debaryomycetaceae |  |
| Fungi | 205840 | *Pseudeurotium hygrophilum* | 1 | 0.036 | 0.004 | Pseudeurotiaceae |  |
| Fungi | 4959 | *Debaryomyces hansenii* | 1 | 0.036 | 0.004 | Debaryomycetaceae |  |
| Fungi | 88768 | *Metschnikowia sp. IFO1406* | 1 | 0.036 | 0.004 | Metschnikowiaceae |  |
| Fungi | 34381 | *Aspergillus japonicus* | 1 | 0.036 | 0.004 | Aspergillaceae |  |
| Fungi | 349255 | *Metschnikowia cibodasensis* | 1 | 0.036 | 0.004 | Metschnikowiaceae |  |
| Bacteria | 148814 | *Lactobacillus kunkeei* | 37752 | 44.111 | 142.513 | Lactobacillaceae |  |
| Bacteria | 542 | *Zymomonas mobilis* | 11721 | 13.695 | 44.246 | Sphingomonadaceae |  |
| Bacteria | 338565 | *Erwinia tasmaniensis* | 1188 | 1.388 | 4.485 | Erwiniaceae |  |
| Bacteria | 552 | *Erwinia amylovora* | 1173 | 1.371 | 4.428 | Erwiniaceae |  |
| Bacteria | 182337 | *Erwinia billingiae* | 996 | 1.164 | 3.760 | Erwiniaceae |  |
| Bacteria | 442 | *Gluconobacter oxydans* | 818 | 0.956 | 3.088 | Acetobacteraceae |  |
| Bacteria | 1396 | *Bacillus cereus* | 783 | 0.915 | 2.956 | Bacillaceae |  |
| Bacteria | 215689 | *Erwinia sp. Ejp617* | 762 | 0.890 | 2.877 | Erwiniaceae |  |
| Bacteria | 1906742 | *Microbacterium sp. BH-3-3-3* | 750 | 0.876 | 2.831 | Microbacteriaceae |  |
| Bacteria | 2033 | *Microbacterium testaceum* | 715 | 0.835 | 2.699 | Microbacteriaceae |  |
| Bacteria | 79967 | *Erwinia pyrifoliae* | 623 | 0.728 | 2.352 | Erwiniaceae |  |
| Bacteria | 28901 | *Salmonella enterica* | 602 | 0.703 | 2.273 | Enterobacteriaceae |  |
| Bacteria | 549 | *Pantoea agglomerans* | 568 | 0.664 | 2.144 | Erwiniaceae |  |
| Bacteria | 1520 | *Clostridium beijerinckii* | 480 | 0.561 | 1.812 | Clostridiaceae |  |
| Bacteria | 1484157 | *Pantoea sp. PSNIH2* | 470 | 0.549 | 1.774 | Erwiniaceae |  |
| Bacteria | 1891675 | *Pantoea alhagi* | 441 | 0.515 | 1.665 | Erwiniaceae |  |
| Bacteria | 1484158 | *Pantoea sp. PSNIH1* | 430 | 0.502 | 1.623 | Erwiniaceae |  |
| Bacteria | 66269 | *Pantoea stewartii* | 430 | 0.502 | 1.623 | Erwiniaceae |  |
| Bacteria | 470934 | *Pantoea vagans* | 424 | 0.495 | 1.601 | Erwiniaceae |  |
| Bacteria | 431306 | *Acetobacter ghanensis* | 414 | 0.484 | 1.563 | Acetobacteraceae |  |
| Bacteria | 1619313 | *Erwinia gerundensis* | 414 | 0.484 | 1.563 | Erwiniaceae |  |
| Bacteria | 592316 | *Pantoea sp. At-9b* | 395 | 0.462 | 1.491 | Erwiniaceae |  |
| Bacteria | 615 | *Serratia marcescens* | 382 | 0.446 | 1.442 | Yersiniaceae |  |
| Bacteria | 891974 | *Plautia stali symbiont* | 379 | 0.443 | 1.431 | Enterobacteriaceae |  |
| Bacteria | 638 | *Arsenophonus nasoniae* | 364 | 0.425 | 1.374 | Morganellaceae |  |
| Bacteria | 53336 | *Tatumella citrea* | 348 | 0.407 | 1.314 | Erwiniaceae |  |
| Bacteria | 550 | *Enterobacter cloacae* | 325 | 0.380 | 1.227 | Enterobacteriaceae |  |
| Bacteria | 158822 | *Cedecea neteri* | 299 | 0.349 | 1.129 | Enterobacteriaceae |  |
| Bacteria | 1076596 | *Acetobacter persici* | 299 | 0.349 | 1.129 | Acetobacteraceae |  |
| Bacteria | 438 | *Acetobacter pasteurianus* | 297 | 0.347 | 1.121 | Acetobacteraceae |  |
| Bacteria | 553 | *Pantoea ananatis* | 296 | 0.346 | 1.117 | Erwiniaceae |  |
| Bacteria | 562 | *Escherichia coli* | 293 | 0.342 | 1.106 | Enterobacteriaceae |  |
| Bacteria | 446692 | *Acetobacter senegalensis* | 291 | 0.340 | 1.099 | Acetobacteraceae |  |
| Bacteria | 573 | *Klebsiella pneumoniae* | 264 | 0.308 | 0.997 | Enterobacteriaceae |  |
| Bacteria | 1076550 | *Pantoea rwandensis* | 246 | 0.287 | 0.929 | Erwiniaceae |  |
| Bacteria | 61652 | *Serratia rubidaea* | 233 | 0.272 | 0.880 | Yersiniaceae |  |
| Bacteria | 1692238 | *Enterobacter sp. FY-07* | 230 | 0.269 | 0.868 | Enterobacteriaceae |  |
| Bacteria | 545 | *Citrobacter koseri* | 218 | 0.255 | 0.823 | Enterobacteriaceae |  |
| Bacteria | 28152 | *Yersinia kristensenii* | 218 | 0.255 | 0.823 | Yersiniaceae |  |
| Bacteria | 554 | *Pectobacterium carotovorum* | 215 | 0.251 | 0.812 | Pectobacteriaceae |  |
| Bacteria | 546 | *Citrobacter freundii* | 208 | 0.243 | 0.785 | Enterobacteriaceae |  |
| Bacteria | 47917 | *Serratia fonticola* | 208 | 0.243 | 0.785 | Yersiniaceae |  |
| Bacteria | 54291 | *Raoultella ornithinolytica* | 189 | 0.221 | 0.713 | Enterobacteriaceae |  |
| Bacteria | 1358 | *Lactococcus lactis* | 181 | 0.211 | 0.683 | Streptococcaceae |  |
| Bacteria | 571 | *Klebsiella oxytoca* | 169 | 0.197 | 0.638 | Enterobacteriaceae |  |
| Bacteria | 563 | *Shimwellia blattae* | 168 | 0.196 | 0.634 | Enterobacteriaceae |  |
| Bacteria | 61645 | *Enterobacter asburiae* | 163 | 0.190 | 0.615 | Enterobacteriaceae |  |
| Bacteria | 33970 | *Melissococcus plutonius* | 153 | 0.179 | 0.578 | Enterococcaceae |  |
| Bacteria | 82996 | *Serratia plymuthica* | 151 | 0.176 | 0.570 | Yersiniaceae |  |
| Bacteria | 1324350 | *Acinetobacter equi* | 146 | 0.171 | 0.551 | Moraxellaceae |  |
| Bacteria | 1510841 | *Parasaccharibacter apium* | 136 | 0.159 | 0.513 | Acetobacteraceae |  |
| Bacteria | 487316 | *Acinetobacter soli* | 132 | 0.154 | 0.498 | Moraxellaceae |  |
| Bacteria | 158836 | *Enterobacter hormaechei* | 130 | 0.152 | 0.491 | Enterobacteriaceae |  |
| Bacteria | 35703 | *Citrobacter amalonaticus* | 129 | 0.151 | 0.487 | Enterobacteriaceae |  |
| Bacteria | 73098 | *Kluyvera georgiana* | 119 | 0.139 | 0.449 | Enterobacteriaceae |  |
| Bacteria | 1166130 | *Enterobacter sp. R4-368* | 119 | 0.139 | 0.449 | Enterobacteriaceae |  |
| Bacteria | 318683 | *Gluconobacter albidus* | 112 | 0.131 | 0.423 | Acetobacteraceae |  |
| Bacteria | 1089444 | *Dickeya solani* | 112 | 0.131 | 0.423 | Pectobacteriaceae |  |
| Bacteria | 470 | *Acinetobacter baumannii* | 112 | 0.131 | 0.423 | Moraxellaceae |  |
| Bacteria | 67825 | *Citrobacter rodentium* | 110 | 0.129 | 0.415 | Enterobacteriaceae |  |
| Bacteria | 57706 | *Citrobacter braakii* | 108 | 0.126 | 0.408 | Enterobacteriaceae |  |
| Bacteria | 1134687 | *Klebsiella michiganensis* | 103 | 0.120 | 0.389 | Enterobacteriaceae |  |
| Bacteria | 40215 | *Acinetobacter junii* | 100 | 0.117 | 0.377 | Moraxellaceae |  |
| Bacteria | 1196095 | *Gilliamella apicola* | 100 | 0.117 | 0.377 | Orbaceae |  |
| Bacteria | 153496 | *Kozakia baliensis* | 98 | 0.115 | 0.370 | Acetobacteraceae |  |
| Bacteria | 29486 | *Yersinia ruckeri* | 98 | 0.115 | 0.370 | Yersiniaceae |  |
| Bacteria | 435 | *Acetobacter aceti* | 97 | 0.113 | 0.366 | Acetobacteraceae |  |
| Bacteria | 208223 | *Kosakonia cowanii* | 97 | 0.113 | 0.366 | Enterobacteriaceae |  |
| Bacteria | 204042 | *Dickeya zeae* | 95 | 0.111 | 0.359 | Pectobacteriaceae |  |
| Bacteria | 28151 | *Serratia proteamaculans* | 93 | 0.109 | 0.351 | Yersiniaceae |  |
| Bacteria | 83655 | *Leclercia adecarboxylata* | 92 | 0.107 | 0.347 | Enterobacteriaceae |  |
| Bacteria | 61647 | *Pluralibacter gergoviae* | 89 | 0.104 | 0.336 | Enterobacteriaceae |  |
| Bacteria | 413501 | *Cronobacter muytjensii* | 88 | 0.103 | 0.332 | Enterobacteriaceae |  |
| Bacteria | 693444 | *Enterobacteriaceae bacterium strain FGI 57* | 88 | 0.103 | 0.332 | Enterobacteriaceae |  |
| Bacteria | 61646 | *Lelliottia amnigena* | 86 | 0.100 | 0.325 | Enterobacteriaceae |  |
| Bacteria | 614 | *Serratia liquefaciens* | 84 | 0.098 | 0.317 | Yersiniaceae |  |
| Bacteria | 91915 | *Asaia bogorensis* | 82 | 0.096 | 0.310 | Acetobacteraceae |  |
| Bacteria | 1646498 | *Acinetobacter sp. TTH0-4* | 82 | 0.096 | 0.310 | Moraxellaceae |  |
| Bacteria | 1789224 | *Acinetobacter larvae* | 81 | 0.095 | 0.306 | Moraxellaceae |  |
| Bacteria | 33996 | *Gluconacetobacter diazotrophicus* | 81 | 0.095 | 0.306 | Acetobacteraceae |  |
| Bacteria | 497725 | *Kosakonia oryzae* | 79 | 0.092 | 0.298 | Enterobacteriaceae |  |
| Bacteria | 61648 | *Kluyvera intermedia* | 79 | 0.092 | 0.298 | Enterobacteriaceae |  |
| Bacteria | 1597 | *Lactobacillus paracasei* | 79 | 0.092 | 0.298 | Lactobacillaceae |  |
| Bacteria | 62977 | *Acinetobacter sp. ADP1* | 78 | 0.091 | 0.294 | Moraxellaceae |  |
| Bacteria | 138074 | *Serratia symbiotica* | 78 | 0.091 | 0.294 | Yersiniaceae |  |
| Bacteria | 34038 | *Rahnella aquatilis* | 77 | 0.090 | 0.291 | Yersiniaceae |  |
| Bacteria | 106654 | *Acinetobacter nosocomialis* | 75 | 0.088 | 0.283 | Moraxellaceae |  |
| Bacteria | 48296 | *Acinetobacter pittii* | 73 | 0.085 | 0.276 | Moraxellaceae |  |
| Bacteria | 1407071 | *Acinetobacter sp. TGL-Y2* | 73 | 0.085 | 0.276 | Moraxellaceae |  |
| Bacteria | 33995 | *Komagataeibacter europaeus* | 73 | 0.085 | 0.276 | Acetobacteraceae |  |
| Bacteria | 29430 | *Acinetobacter haemolyticus* | 73 | 0.085 | 0.276 | Moraxellaceae |  |
| Bacteria | 413497 | *Cronobacter dublinensis* | 72 | 0.084 | 0.272 | Enterobacteriaceae |  |
| Bacteria | 40214 | *Acinetobacter johnsonii* | 71 | 0.083 | 0.268 | Moraxellaceae |  |
| Bacteria | 1639108 | *Chania multitudinisentens* | 68 | 0.079 | 0.257 | Yersiniaceae |  |
| Bacteria | 1177712 | *Komagataeibacter medellinensis* | 66 | 0.077 | 0.249 | Acetobacteraceae |  |
| Bacteria | 28090 | *Acinetobacter lwoffii* | 65 | 0.076 | 0.245 | Moraxellaceae |  |
| Bacteria | 1334193 | *Enterobacter lignolyticus* | 65 | 0.076 | 0.245 | Enterobacteriaceae |  |
| Bacteria | 633 | *Yersinia pseudotuberculosis* | 65 | 0.076 | 0.245 | Yersiniaceae |  |
| Bacteria | 106649 | *Acinetobacter guillouiae* | 64 | 0.075 | 0.242 | Moraxellaceae |  |
| Bacteria | 2133 | *Spiroplasma citri* | 64 | 0.075 | 0.242 | Spiroplasmataceae |  |
| Bacteria | 375175 | *Lactobacillus backii* | 63 | 0.074 | 0.238 | Lactobacillaceae |  |
| Bacteria | 1686310 | *Bartonella apis* | 62 | 0.072 | 0.234 | Bartonellaceae |  |
| Bacteria | 28141 | *Cronobacter sakazakii* | 60 | 0.070 | 0.226 | Enterobacteriaceae |  |
| Bacteria | 1163710 | *Cronobacter condimenti* | 59 | 0.069 | 0.223 | Enterobacteriaceae |  |
| Bacteria | 28448 | *Komagataeibacter xylinus* | 58 | 0.068 | 0.219 | Acetobacteraceae |  |
| Bacteria | 630 | *Yersinia enterocolitica* | 58 | 0.068 | 0.219 | Yersiniaceae |  |
| Bacteria | 935293 | *Yersinia entomophaga* | 57 | 0.067 | 0.215 | Yersiniaceae |  |
| Bacteria | 204038 | *Dickeya dadantii* | 57 | 0.067 | 0.215 | Pectobacteriaceae |  |
| Bacteria | 1158459 | *Kosakonia sacchari* | 57 | 0.067 | 0.215 | Enterobacteriaceae |  |
| Bacteria | 471 | *Acinetobacter calcoaceticus* | 57 | 0.067 | 0.215 | Moraxellaceae |  |
| Bacteria | 1428 | *Bacillus thuringiensis* | 56 | 0.065 | 0.211 | Bacillaceae |  |
| Bacteria | 1351 | *Enterococcus faecalis* | 55 | 0.064 | 0.208 | Enterococcaceae |  |
| Bacteria | 399742 | *Enterobacter sp. 638* | 55 | 0.064 | 0.208 | Enterobacteriaceae |  |
| Bacteria | 1608473 | *Acinetobacter sp. NCu2D-2* | 55 | 0.064 | 0.208 | Moraxellaceae |  |
| Bacteria | 548 | *Klebsiella aerogenes* | 54 | 0.063 | 0.204 | Enterobacteriaceae |  |
| Bacteria | 1633874 | *Acetobacter sp. SLV-7* | 50 | 0.058 | 0.189 | Acetobacteraceae |  |
| Bacteria | 54736 | *Salmonella bongori* | 49 | 0.057 | 0.185 | Enterobacteriaceae |  |
| Bacteria | 263819 | *Yersinia aleksiciae* | 47 | 0.055 | 0.177 | Yersiniaceae |  |
| Bacteria | 587 | *Providencia rettgeri* | 46 | 0.054 | 0.174 | Morganellaceae |  |
| Bacteria | 569 | *Hafnia alvei* | 44 | 0.051 | 0.166 | Hafniaceae |  |
| Bacteria | 1148157 | *Acinetobacter oleivorans* | 44 | 0.051 | 0.166 | Moraxellaceae |  |
| Bacteria | 265960 | *Komagataeibacter nataicola* | 43 | 0.050 | 0.162 | Acetobacteraceae |  |
| Bacteria | 82985 | *Pragia fontium* | 42 | 0.049 | 0.159 | Budviciaceae |  |
| Bacteria | 29485 | *Yersinia rohdei* | 42 | 0.049 | 0.159 | Yersiniaceae |  |
| Bacteria | 108981 | *Acinetobacter schindleri* | 40 | 0.047 | 0.151 | Moraxellaceae |  |
| Bacteria | 93218 | *Pandoraea apista* | 39 | 0.046 | 0.147 | Burkholderiaceae |  |
| Bacteria | 535744 | *Cronobacter universalis* | 39 | 0.046 | 0.147 | Enterobacteriaceae |  |
| Bacteria | 67780 | *Edwardsiella ictaluri* | 38 | 0.044 | 0.143 | Hafniaceae |  |
| Bacteria | 208962 | *Escherichia albertii* | 38 | 0.044 | 0.143 | Enterobacteriaceae |  |
| Bacteria | 413502 | *Cronobacter turicensis* | 37 | 0.043 | 0.140 | Enterobacteriaceae |  |
| Bacteria | 215221 | *Komagataeibacter rhaeticus* | 37 | 0.043 | 0.140 | Acetobacteraceae |  |
| Bacteria | 1785128 | *Acinetobacter lactucae* | 37 | 0.043 | 0.140 | Moraxellaceae |  |
| Bacteria | 1759437 | *Serratia sp. YD25* | 36 | 0.042 | 0.136 | Yersiniaceae |  |
| Bacteria | 67827 | *Citrobacter werkmanii* | 36 | 0.042 | 0.136 | Enterobacteriaceae |  |
| Bacteria | 1196083 | *Snodgrassella alvi* | 35 | 0.041 | 0.132 | Neisseriaceae |  |
| Bacteria | 1239307 | *Sodalis praecaptivus* | 35 | 0.041 | 0.132 | Pectobacteriaceae |  |
| Bacteria | 1702170 | *Citrobacter sp. FDAARGOS_156* | 34 | 0.040 | 0.128 | Enterobacteriaceae |  |
| Bacteria | 55208 | *Pectobacterium wasabiae* | 33 | 0.039 | 0.125 | Pectobacteriaceae |  |
| Bacteria | 1545702 | *Lactobacillus sp. wkB8* | 33 | 0.039 | 0.125 | Lactobacillaceae |  |
| Bacteria | 29483 | *Yersinia aldovae* | 33 | 0.039 | 0.125 | Yersiniaceae |  |
| Bacteria | 82983 | *Obesumbacterium proteus* | 32 | 0.037 | 0.121 | Hafniaceae |  |
| Bacteria | 63612 | *Sodalis glossinidius* | 32 | 0.037 | 0.121 | Pectobacteriaceae |  |
| Bacteria | 29488 | *Photorhabdus luminescens* | 31 | 0.036 | 0.117 | Morganellaceae |  |
| Bacteria | 1915310 | *Enterobacter cloacae complex sp. ECNIH7* | 31 | 0.036 | 0.117 | Enterobacteriaceae |  |
| Bacteria | 93378 | *Edwardsiella hoshinae* | 30 | 0.035 | 0.113 | Hafniaceae |  |
| Bacteria | 367190 | *Yersinia similis* | 29 | 0.034 | 0.109 | Yersiniaceae |  |
| Bacteria | 40576 | *Xenorhabdus bovienii* | 29 | 0.034 | 0.109 | Morganellaceae |  |
| Bacteria | 291112 | *Photorhabdus asymbiotica* | 27 | 0.032 | 0.102 | Morganellaceae |  |
| Bacteria | 59814 | *Pantoea dispersa* | 27 | 0.032 | 0.102 | Erwiniaceae |  |
| Bacteria | 1267021 | *Frischella perrara* | 27 | 0.032 | 0.102 | Orbaceae |  |
| Bacteria | 1905730 | *Pectobacterium parmentieri* | 26 | 0.030 | 0.098 | Pectobacteriaceae |  |
| Bacteria | 29471 | *Pectobacterium atrosepticum* | 26 | 0.030 | 0.098 | Pectobacteriaceae |  |
| Bacteria | 636 | *Edwardsiella tarda* | 26 | 0.030 | 0.098 | Hafniaceae |  |
| Bacteria | 1463165 | *Klebsiella quasipneumoniae* | 25 | 0.029 | 0.094 | Enterobacteriaceae |  |
| Bacteria | 584 | *Proteus mirabilis* | 25 | 0.029 | 0.094 | Morganellaceae |  |
| Bacteria | 1265478 | *Enterobacteriaceae bacterium bta3-1* | 25 | 0.029 | 0.094 | Enterobacteriaceae |  |
| Bacteria | 564 | *Escherichia fergusonii* | 25 | 0.029 | 0.094 | Enterobacteriaceae |  |
| Bacteria | 283686 | *Kosakonia radicincitans* | 25 | 0.029 | 0.094 | Enterobacteriaceae |  |
| Bacteria | 300019 | *Microbacterium paludicola* | 23 | 0.027 | 0.087 | Microbacteriaceae |  |
| Bacteria | 320497 | *Neoasaia chiangmaiensis* | 23 | 0.027 | 0.087 | Acetobacteraceae |  |
| Bacteria | 1218496 | *Lactobacillus sp. Fhon2N* | 22 | 0.026 | 0.083 | Lactobacillaceae |  |
| Bacteria | 1934254 | *Klebsiella sp. M5al* | 22 | 0.026 | 0.083 | Enterobacteriaceae |  |
| Bacteria | 55212 | *Erwinia rhapontici* | 21 | 0.025 | 0.079 | Erwiniaceae |  |
| Bacteria | 469595 | *Citrobacter sp. 30_2* | 21 | 0.025 | 0.079 | Enterobacteriaceae |  |
| Bacteria | 351679 | *Xenorhabdus hominickii* | 20 | 0.023 | 0.075 | Morganellaceae |  |
| Bacteria | 2019568 | *Citrobacter sp. 92* | 20 | 0.023 | 0.075 | Enterobacteriaceae |  |
| Bacteria | 370764 | *Microbacterium pygmaeum* | 20 | 0.023 | 0.075 | Microbacteriaceae |  |
| Bacteria | 912630 | *Microbacterium sp. LKL04* | 19 | 0.022 | 0.072 | Microbacteriaceae |  |
| Bacteria | 1245 | *Leuconostoc mesenteroides* | 19 | 0.022 | 0.072 | Leuconostocaceae |  |
| Bacteria | 1560339 | *Enterobacter sp. E20* | 19 | 0.022 | 0.072 | Enterobacteriaceae |  |
| Bacteria | 1714373 | *Microbacterium sp. No. 7* | 19 | 0.022 | 0.072 | Microbacteriaceae |  |
| Bacteria | 1870930 | *Enterobacter cloacae complex sp. 35734* | 19 | 0.022 | 0.072 | Enterobacteriaceae |  |
| Bacteria | 187493 | *Thalassolituus oleivorans* | 18 | 0.021 | 0.068 | Oceanospirillaceae |  |
| Bacteria | 1486991 | *Candidatus Sodalis pierantonius* | 18 | 0.021 | 0.068 | Pectobacteriaceae |  |
| Bacteria | 36805 | *Microbacterium aurum* | 17 | 0.020 | 0.064 | Microbacteriaceae |  |
| Bacteria | 351671 | *Xenorhabdus doucetiae* | 17 | 0.020 | 0.064 | Morganellaceae |  |
| Bacteria | 157463 | *Fructobacillus ficulneus* | 17 | 0.020 | 0.064 | Leuconostocaceae |  |
| Bacteria | 1263550 | *Edwardsiella piscicida* | 17 | 0.020 | 0.064 | Hafniaceae |  |
| Bacteria | 138072 | *Candidatus Hamiltonella defensa* | 17 | 0.020 | 0.064 | Enterobacteriaceae |  |
| Bacteria | 84292 | *Microbacterium chocolatum* | 16 | 0.019 | 0.060 | Microbacteriaceae |  |
| Bacteria | 1812935 | *Enterobacter cloacae complex 'Hoffmann cluster IV'* | 16 | 0.019 | 0.060 | Enterobacteriaceae |  |
| Bacteria | 703 | *Plesiomonas shigelloides* | 16 | 0.019 | 0.060 | NO_NAME |  |
| Bacteria | 104336 | *Microbacterium foliorum* | 15 | 0.018 | 0.057 | Microbacteriaceae |  |
| Bacteria | 1327989 | *Serratia sp. FS14* | 15 | 0.018 | 0.057 | Yersiniaceae |  |
| Bacteria | 574560 | *Photorhabdus temperata* | 14 | 0.016 | 0.053 | Morganellaceae |  |
| Bacteria | 244366 | *Klebsiella variicola* | 14 | 0.016 | 0.053 | Enterobacteriaceae |  |
| Bacteria | 28447 | *Clavibacter michiganensis* | 14 | 0.016 | 0.053 | Microbacteriaceae |  |
| Bacteria | 1544694 | *Rosenbergiella epipactidis* | 14 | 0.016 | 0.053 | Enterobacteriaceae |  |
| Bacteria | 1868135 | *Enterobacter sp. HK169* | 14 | 0.016 | 0.053 | Enterobacteriaceae |  |
| Bacteria | 1848580 | *Hafnia sp. CBA7124* | 14 | 0.016 | 0.053 | Hafniaceae |  |
| Bacteria | 1839800 | *Yersinia sp. FDAARGOS_228* | 14 | 0.016 | 0.053 | Yersiniaceae |  |
| Bacteria | 1582 | *Lactobacillus casei* | 14 | 0.016 | 0.053 | Lactobacillaceae |  |
| Bacteria | 670 | *Vibrio parahaemolyticus* | 14 | 0.016 | 0.053 | Vibrionaceae |  |
| Bacteria | 1697053 | *Oblitimonas alkaliphila* | 14 | 0.016 | 0.053 | Pseudomonadaceae |  |
| Bacteria | 1590 | *Lactobacillus plantarum* | 14 | 0.016 | 0.053 | Lactobacillaceae |  |
| Bacteria | 628 | *Xenorhabdus nematophila* | 12 | 0.014 | 0.045 | Morganellaceae |  |
| Bacteria | 676 | *Vibrio fluvialis* | 12 | 0.014 | 0.045 | Vibrionaceae |  |
| Bacteria | 33964 | *Leuconostoc citreum* | 12 | 0.014 | 0.045 | Leuconostocaceae |  |
| Bacteria | 299767 | *Enterobacter ludwigii* | 11 | 0.013 | 0.042 | Enterobacteriaceae |  |
| Bacteria | 40324 | *Stenotrophomonas maltophilia* | 11 | 0.013 | 0.042 | Xanthomonadaceae |  |
| Bacteria | 40577 | *Xenorhabdus poinarii* | 11 | 0.013 | 0.042 | Morganellaceae |  |
| Bacteria | 1809055 | *Acinetobacter sp. DUT-2* | 10 | 0.012 | 0.038 | Moraxellaceae |  |
| Bacteria | 654 | *Aeromonas veronii* | 10 | 0.012 | 0.038 | Aeromonadaceae |  |
| Bacteria | 1827481 | *Enterobacter sp. ODB01* | 10 | 0.012 | 0.038 | Enterobacteriaceae |  |
| Bacteria | 665097 | *Erwinia piriflorinigrans* | 10 | 0.012 | 0.038 | Erwiniaceae |  |
| Bacteria | 255248 | *Leuconostoc garlicum* | 10 | 0.012 | 0.038 | Leuconostocaceae |  |
| Bacteria | 2137 | *Spiroplasma apis* | 10 | 0.012 | 0.038 | Spiroplasmataceae |  |
| Bacteria | 294 | *Pseudomonas fluorescens* | 9 | 0.011 | 0.034 | Pseudomonadaceae |  |
| Bacteria | 644 | *Aeromonas hydrophila* | 9 | 0.011 | 0.034 | Aeromonadaceae |  |
| Bacteria | 36873 | *Paraburkholderia xenovorans* | 9 | 0.011 | 0.034 | Burkholderiaceae |  |
| Bacteria | 202956 | *Acinetobacter towneri* | 9 | 0.011 | 0.034 | Moraxellaceae |  |
| Bacteria | 1219383 | *Acinetobacter boissieri* | 9 | 0.011 | 0.034 | Moraxellaceae |  |
| Bacteria | 69222 | *Erwinia mallotivora* | 9 | 0.011 | 0.034 | Erwiniaceae |  |
| Bacteria | 488142 | *Serratia sp. SCBI* | 8 | 0.009 | 0.030 | Yersiniaceae |  |
| Bacteria | 588 | *Providencia stuartii* | 8 | 0.009 | 0.030 | Morganellaceae |  |
| Bacteria | 1246 | *Leuconostoc lactis* | 8 | 0.009 | 0.030 | Leuconostocaceae |  |
| Bacteria | 146474 | *Acetobacter orientalis* | 8 | 0.009 | 0.030 | Acetobacteraceae |  |
| Bacteria | 1812934 | *Enterobacter cloacae complex 'Hoffmann cluster III'* | 8 | 0.009 | 0.030 | Enterobacteriaceae |  |
| Bacteria | 1938334 | *Microbacterium sp. TPU 3598* | 8 | 0.009 | 0.030 | Microbacteriaceae |  |
| Bacteria | 53444 | *Lactobacillus lindneri* | 8 | 0.009 | 0.030 | Lactobacillaceae |  |
| Bacteria | 727 | *Haemophilus influenzae* | 8 | 0.009 | 0.030 | Pasteurellaceae |  |
| Bacteria | 1905288 | *Klebsiella sp. LTGPAF-6F* | 8 | 0.009 | 0.030 | Enterobacteriaceae |  |
| Bacteria | 1280052 | *Acinetobacter sp. M131* | 7 | 0.008 | 0.026 | Moraxellaceae |  |
| Bacteria | 80852 | *Aliivibrio wodanis* | 7 | 0.008 | 0.026 | Vibrionaceae |  |
| Bacteria | 1575 | *Leifsonia xyli* | 7 | 0.008 | 0.026 | Microbacteriaceae |  |
| Bacteria | 412690 | *Microterricola viridarii* | 7 | 0.008 | 0.026 | Microbacteriaceae |  |
| Bacteria | 741091 | *Rahnella sp. Y9602* | 7 | 0.008 | 0.026 | Yersiniaceae |  |
| Bacteria | 190893 | *Vibrio coralliilyticus* | 7 | 0.008 | 0.026 | Vibrionaceae |  |
| Bacteria | 1980001 | *Cellulosimicrobium sp. TH-20* | 7 | 0.008 | 0.026 | Promicromonosporaceae |  |
| Bacteria | 631 | *Yersinia intermedia* | 7 | 0.008 | 0.026 | Yersiniaceae |  |
| Bacteria | 1244 | *Leuconostoc gelidum* | 7 | 0.008 | 0.026 | Leuconostocaceae |  |
| Bacteria | 68334 | *Erwinia aphidicola* | 7 | 0.008 | 0.026 | Erwiniaceae |  |
| Bacteria | 1166016 | *Pectobacterium sp. SCC3193* | 7 | 0.008 | 0.026 | Pectobacteriaceae |  |
| Bacteria | 47834 | *Spiroplasma kunkelii* | 7 | 0.008 | 0.026 | Spiroplasmataceae |  |
| Bacteria | 738 | *[Haemophilus] parasuis* | 7 | 0.008 | 0.026 | Pasteurellaceae |  |
| Bacteria | 34062 | *Moraxella osloensis* | 7 | 0.008 | 0.026 | Moraxellaceae |  |
| Bacteria | 1390395 | *Sphingomonas sp. LK11* | 6 | 0.007 | 0.023 | Sphingomonadaceae |  |
| Bacteria | 1501 | *Clostridium pasteurianum* | 6 | 0.007 | 0.023 | Clostridiaceae |  |
| Bacteria | 150123 | *Plantibacter flavus* | 6 | 0.007 | 0.023 | Microbacteriaceae |  |
| Bacteria | 668 | *Aliivibrio fischeri* | 6 | 0.007 | 0.023 | Vibrionaceae |  |
| Bacteria | 1930557 | *Shewanella sp. FDAARGOS_354* | 6 | 0.007 | 0.023 | Shewanellaceae |  |
| Bacteria | 1435069 | *Vibrio tritonius* | 6 | 0.007 | 0.023 | Vibrionaceae |  |
| Bacteria | 74109 | *Photobacterium profundum* | 6 | 0.007 | 0.023 | Vibrionaceae |  |
| Bacteria | 657337 | *Rahnella sp. WMR66* | 6 | 0.007 | 0.023 | Yersiniaceae |  |
| Bacteria | 1563632 | *gamma proteobacterium symbiont of Plautia stali* | 6 | 0.007 | 0.023 | NO_NAME |  |
| Bacteria | 367477 | *Microbacterium sp. XT11* | 6 | 0.007 | 0.023 | Microbacteriaceae |  |
| Bacteria | 453304 | *Agromyces aureus* | 6 | 0.007 | 0.023 | Microbacteriaceae |  |
| Bacteria | 55211 | *Erwinia persicina* | 6 | 0.007 | 0.023 | Erwiniaceae |  |
| Bacteria | 1252 | *Leuconostoc carnosum* | 6 | 0.007 | 0.023 | Leuconostocaceae |  |
| Bacteria | 472695 | *Pantoea septica* | 6 | 0.007 | 0.023 | Erwiniaceae |  |
| Bacteria | 347 | *Xanthomonas oryzae* | 5 | 0.006 | 0.019 | Xanthomonadaceae |  |
| Bacteria | 28108 | *Alteromonas macleodii* | 5 | 0.006 | 0.019 | Alteromonadaceae |  |
| Bacteria | 1678128 | *Limnohabitans sp. 63ED37-2* | 5 | 0.006 | 0.019 | Comamonadaceae |  |
| Bacteria | 528191 | *Paenibacillus xylanexedens* | 5 | 0.006 | 0.019 | Paenibacillaceae |  |
| Bacteria | 44012 | *Ferrimonas balearica* | 5 | 0.006 | 0.019 | Ferrimonadaceae |  |
| Bacteria | 43948 | *Tolumonas auensis* | 5 | 0.006 | 0.019 | Aeromonadaceae |  |
| Bacteria | 1853130 | *Pseudomonas sp. A3(2016)* | 5 | 0.006 | 0.019 | Pseudomonadaceae |  |
| Bacteria | 759620 | *Weissella ceti* | 5 | 0.006 | 0.019 | Leuconostocaceae |  |
| Bacteria | 571800 | *Psychrobacter sp. G* | 5 | 0.006 | 0.019 | Moraxellaceae |  |
| Bacteria | 45658 | *Vibrio scophthalmi* | 5 | 0.006 | 0.019 | Vibrionaceae |  |
| Bacteria | 357794 | *Psychromonas ingrahamii* | 5 | 0.006 | 0.019 | Psychromonadaceae |  |
| Bacteria | 1684 | *Bifidobacterium asteroides* | 5 | 0.006 | 0.019 | Bifidobacteriaceae |  |
| Bacteria | 137591 | *Weissella cibaria* | 5 | 0.006 | 0.019 | Leuconostocaceae |  |
| Bacteria | 38293 | *Photobacterium damselae* | 5 | 0.006 | 0.019 | Vibrionaceae |  |
| Bacteria | 1464 | *Paenibacillus larvae* | 5 | 0.006 | 0.019 | Paenibacillaceae |  |
| Bacteria | 317 | *Pseudomonas syringae* | 5 | 0.006 | 0.019 | Pseudomonadaceae |  |
| Bacteria | 1235990 | *Candidatus Pantoea carbekii* | 5 | 0.006 | 0.019 | Erwiniaceae |  |
| Bacteria | 199592 | *Microbacterium paraoxydans* | 5 | 0.006 | 0.019 | Microbacteriaceae |  |
| Bacteria | 1795053 | *Microbacterium sp. PAMC 28756* | 5 | 0.006 | 0.019 | Microbacteriaceae |  |
| Bacteria | 55601 | *Vibrio anguillarum* | 5 | 0.006 | 0.019 | Vibrionaceae |  |
| Bacteria | 364410 | *Granulibacter bethesdensis* | 5 | 0.006 | 0.019 | Acetobacteraceae |  |
| Bacteria | 93973 | *Shewanella japonica* | 5 | 0.006 | 0.019 | Shewanellaceae |  |
| Bacteria | 1916917 | *Microbacterium sp. 1.5R* | 5 | 0.006 | 0.019 | Microbacteriaceae |  |
| Bacteria | 216778 | *Stenotrophomonas rhizophila* | 4 | 0.005 | 0.015 | Xanthomonadaceae |  |
| Bacteria | 669 | *Vibrio harveyi* | 4 | 0.005 | 0.015 | Vibrionaceae |  |
| Bacteria | 1892404 | *Bacillus sp. ABP14* | 4 | 0.005 | 0.015 | Bacillaceae |  |
| Bacteria | 1905847 | *Curtobacterium sp. BH-2-1-1* | 4 | 0.005 | 0.015 | Microbacteriaceae |  |
| Bacteria | 157673 | *[Mannheimia] succiniciproducens* | 4 | 0.005 | 0.015 | Pasteurellaceae |  |
| Bacteria | 216142 | *Pseudomonas rhizosphaerae* | 4 | 0.005 | 0.015 | Pseudomonadaceae |  |
| Bacteria | 1619308 | *Cnuibacter physcomitrellae* | 4 | 0.005 | 0.015 | Microbacteriaceae |  |
| Bacteria | 47735 | *Bibersteinia trehalosi* | 4 | 0.005 | 0.015 | Pasteurellaceae |  |
| Bacteria | 709323 | *Fructobacillus tropaeoli* | 4 | 0.005 | 0.015 | Leuconostocaceae |  |
| Bacteria | 220714 | *Fructobacillus pseudoficulneus* | 4 | 0.005 | 0.015 | Leuconostocaceae |  |
| Bacteria | 561879 | *Bacillus safensis* | 4 | 0.005 | 0.015 | Bacillaceae |  |
| Bacteria | 28258 | *Cobetia marina* | 4 | 0.005 | 0.015 | Halomonadaceae |  |
| Bacteria | 687 | *Vibrio gazogenes* | 4 | 0.005 | 0.015 | Vibrionaceae |  |
| Bacteria | 62322 | *Shewanella baltica* | 4 | 0.005 | 0.015 | Shewanellaceae |  |
| Bacteria | 1978566 | *Cryobacterium sp. LW097* | 4 | 0.005 | 0.015 | Microbacteriaceae |  |
| Bacteria | 70863 | *Shewanella oneidensis* | 4 | 0.005 | 0.015 | Shewanellaceae |  |
| Bacteria | 730 | *[Haemophilus] ducreyi* | 4 | 0.005 | 0.015 | Pasteurellaceae |  |
| Bacteria | 1778262 | *Candidatus Doolittlea endobia* | 4 | 0.005 | 0.015 | Enterobacteriaceae |  |
| Bacteria | 632 | *Yersinia pestis* | 4 | 0.005 | 0.015 | Yersiniaceae |  |
| Bacteria | 673 | *Grimontia hollisae* | 4 | 0.005 | 0.015 | Vibrionaceae |  |
| Bacteria | 1763998 | *Rheinheimera sp. F8* | 4 | 0.005 | 0.015 | Chromatiaceae |  |
| Bacteria | 1679001 | *Pasteurellaceae bacterium NI1060* | 4 | 0.005 | 0.015 | Pasteurellaceae |  |
| Bacteria | 399736 | *Agrococcus jejuensis* | 4 | 0.005 | 0.015 | Microbacteriaceae |  |
| Bacteria | 47715 | *Lactobacillus rhamnosus* | 4 | 0.005 | 0.015 | Lactobacillaceae |  |
| Bacteria | 143813 | *Pseudomonas sp. LAB-08* | 4 | 0.005 | 0.015 | Pseudomonadaceae |  |
| Bacteria | 104087 | *Pseudomonas frederiksbergensis* | 4 | 0.005 | 0.015 | Pseudomonadaceae |  |
| Bacteria | 121719 | *Pannonibacter phragmitetus* | 4 | 0.005 | 0.015 | Rhodobacteraceae |  |
| Bacteria | 729 | *Haemophilus parainfluenzae* | 4 | 0.005 | 0.015 | Pasteurellaceae |  |
| Bacteria | 684552 | *Agrococcus carbonis* | 4 | 0.005 | 0.015 | Microbacteriaceae |  |
| Bacteria | 147645 | *Paracoccus yeei* | 4 | 0.005 | 0.015 | Rhodobacteraceae |  |
| Bacteria | 58049 | *Colwellia sp. MT41* | 4 | 0.005 | 0.015 | Colwelliaceae |  |
| Bacteria | 97478 | *Lactobacillus mucosae* | 4 | 0.005 | 0.015 | Lactobacillaceae |  |
| Bacteria | 716 | *Actinobacillus suis* | 3 | 0.004 | 0.011 | Pasteurellaceae |  |
| Bacteria | 715 | *Actinobacillus pleuropneumoniae* | 3 | 0.004 | 0.011 | Pasteurellaceae |  |
| Bacteria | 637971 | *Lactobacillus koreensis* | 3 | 0.004 | 0.011 | Lactobacillaceae |  |
| Bacteria | 1491 | *Clostridium botulinum* | 3 | 0.004 | 0.011 | Clostridiaceae |  |
| Bacteria | 1492 | *Clostridium butyricum* | 3 | 0.004 | 0.011 | Clostridiaceae |  |
| Bacteria | 1778877 | *gamma proteobacterium 10BT* | 3 | 0.004 | 0.011 | NO_NAME |  |
| Bacteria | 42906 | *Serratia entomophila* | 3 | 0.004 | 0.011 | Yersiniaceae |  |
| Bacteria | 1534 | *Clostridium kluyveri* | 3 | 0.004 | 0.011 | Clostridiaceae |  |
| Bacteria | 28107 | *Pseudoalteromonas espejiana* | 3 | 0.004 | 0.011 | Pseudoalteromonadaceae |  |
| Bacteria | 1138822 | *Lactobacillus curieae* | 3 | 0.004 | 0.011 | Lactobacillaceae |  |
| Bacteria | 332056 | *Sphingobium japonicum* | 3 | 0.004 | 0.011 | Sphingomonadaceae |  |
| Bacteria | 747 | *Pasteurella multocida* | 3 | 0.004 | 0.011 | Pasteurellaceae |  |
| Bacteria | 511 | *Alcaligenes faecalis* | 3 | 0.004 | 0.011 | Alcaligenaceae |  |
| Bacteria | 126162 | *secondary endosymbiont of Glycaspis brimblecombei* | 3 | 0.004 | 0.011 | Enterobacteriaceae |  |
| Bacteria | 69224 | *Erwinia psidii* | 3 | 0.004 | 0.011 | Erwiniaceae |  |
| Bacteria | 136609 | *Leuconostoc kimchii* | 3 | 0.004 | 0.011 | Leuconostocaceae |  |
| Bacteria | 680 | *Vibrio campbellii* | 3 | 0.004 | 0.011 | Vibrionaceae |  |
| Bacteria | 1903694 | *Oceanisphaera sp. AMac2203* | 3 | 0.004 | 0.011 | Aeromonadaceae |  |
| Bacteria | 92490 | *Erwinia toletana* | 3 | 0.004 | 0.011 | Erwiniaceae |  |
| Bacteria | 76758 | *Pseudomonas orientalis* | 3 | 0.004 | 0.011 | Pseudomonadaceae |  |
| Bacteria | 40216 | *Acinetobacter radioresistens* | 3 | 0.004 | 0.011 | Moraxellaceae |  |
| Bacteria | 47880 | *Pseudomonas fulva* | 3 | 0.004 | 0.011 | Pseudomonadaceae |  |
| Bacteria | 1793722 | *Curtobacterium sp. 9128* | 3 | 0.004 | 0.011 | Microbacteriaceae |  |
| Bacteria | 212663 | *Vibrio tasmaniensis* | 3 | 0.004 | 0.011 | Vibrionaceae |  |
| Bacteria | 28173 | *Vibrio nigripulchritudo* | 3 | 0.004 | 0.011 | Vibrionaceae |  |
| Bacteria | 1218495 | *Lactobacillus apinorum* | 3 | 0.004 | 0.011 | Lactobacillaceae |  |
| Bacteria | 1612 | *Lactobacillus farciminis* | 3 | 0.004 | 0.011 | Lactobacillaceae |  |
| Bacteria | 1363 | *Lactococcus garvieae* | 3 | 0.004 | 0.011 | Streptococcaceae |  |
| Bacteria | 135577 | *Idiomarina loihiensis* | 3 | 0.004 | 0.011 | Idiomarinaceae |  |
| Bacteria | 56812 | *Shewanella frigidimarina* | 3 | 0.004 | 0.011 | Shewanellaceae |  |
| Bacteria | 204039 | *Dickeya dianthicola* | 3 | 0.004 | 0.011 | Pectobacteriaceae |  |
| Bacteria | 198618 | *Pseudomonas umsongensis* | 3 | 0.004 | 0.011 | Pseudomonadaceae |  |
| Bacteria | 28229 | *Colwellia psychrerythraea* | 3 | 0.004 | 0.011 | Colwelliaceae |  |
| Bacteria | 2014742 | *Vibrio sp. 2521-89* | 3 | 0.004 | 0.011 | Vibrionaceae |  |
| Bacteria | 36745 | *Clostridium saccharoperbutylacetonicum* | 3 | 0.004 | 0.011 | Clostridiaceae |  |
| Bacteria | 481743 | *Paenibacillus sp. Y412MC10* | 3 | 0.004 | 0.011 | Paenibacillaceae |  |
| Bacteria | 587753 | *Pseudomonas chlororaphis* | 3 | 0.004 | 0.011 | Pseudomonadaceae |  |
| Bacteria | 314282 | *Psychromonas sp. CNPT3* | 3 | 0.004 | 0.011 | Psychromonadaceae |  |
| Bacteria | 29494 | *Vibrio furnissii* | 3 | 0.004 | 0.011 | Vibrionaceae |  |
| Bacteria | 1352 | *Enterococcus faecium* | 3 | 0.004 | 0.011 | Enterococcaceae |  |
| Bacteria | 330276 | *Bacillus sp. Am 08* | 3 | 0.004 | 0.011 | Bacillaceae |  |
| Bacteria | 337191 | *Gordonia sp. KTR9* | 3 | 0.004 | 0.011 | Gordoniaceae |  |
| Bacteria | 1295392 | *Photobacterium gaetbulicola* | 3 | 0.004 | 0.011 | Vibrionaceae |  |
| Bacteria | 314722 | *Pseudoxanthomonas suwonensis* | 3 | 0.004 | 0.011 | Xanthomonadaceae |  |
| Bacteria | 983545 | *Glaciecola sp. 4H-3-7+YE-5* | 3 | 0.004 | 0.011 | Alteromonadaceae |  |
| Bacteria | 9 | *Buchnera aphidicola* | 3 | 0.004 | 0.011 | Erwiniaceae |  |
| Bacteria | 400668 | *Marinomonas sp. MWYL1* | 3 | 0.004 | 0.011 | Oceanospirillaceae |  |
| Bacteria | 158080 | *Chromohalobacter salexigens* | 3 | 0.004 | 0.011 | Halomonadaceae |  |
| Bacteria | 257708 | *Roseomonas gilardii* | 3 | 0.004 | 0.011 | Acetobacteraceae |  |
| Bacteria | 287 | *Pseudomonas aeruginosa* | 3 | 0.004 | 0.011 | Pseudomonadaceae |  |
| Bacteria | 674 | *Vibrio mimicus* | 3 | 0.004 | 0.011 | Vibrionaceae |  |
| Bacteria | 672 | *Vibrio vulnificus* | 3 | 0.004 | 0.011 | Vibrionaceae |  |
| Bacteria | 1173099 | *bacterium endosymbiont of Rhamphus pulicarius* | 3 | 0.004 | 0.011 | Enterobacteriaceae |  |
| Bacteria | 303 | *Pseudomonas putida* | 3 | 0.004 | 0.011 | Pseudomonadaceae |  |
| Bacteria | 255519 | *Atlantibacter subterranea* | 3 | 0.004 | 0.011 | Enterobacteriaceae |  |
| Bacteria | 376489 | *Halotalea alkalilenta* | 3 | 0.004 | 0.011 | Halomonadaceae |  |
| Bacteria | 1638 | *Listeria ivanovii* | 3 | 0.004 | 0.011 | Listeriaceae |  |
| Bacteria | 1816219 | *Colwellia sp. PAMC 21821* | 3 | 0.004 | 0.011 | Colwelliaceae |  |
| Bacteria | 1096243 | *Idiomarina piscisalsi* | 2 | 0.002 | 0.008 | Idiomarinaceae |  |
| Bacteria | 511062 | *Oceanimonas sp. GK1* | 2 | 0.002 | 0.008 | Aeromonadaceae |  |
| Bacteria | 33888 | *Rathayibacter tritici* | 2 | 0.002 | 0.008 | Microbacteriaceae |  |
| Bacteria | 60217 | *Shewanella violacea* | 2 | 0.002 | 0.008 | Shewanellaceae |  |
| Bacteria | 718 | *Actinobacillus equuli* | 2 | 0.002 | 0.008 | Pasteurellaceae |  |
| Bacteria | 714 | *Aggregatibacter actinomycetemcomitans* | 2 | 0.002 | 0.008 | Pasteurellaceae |  |
| Bacteria | 279828 | *Microcella alkaliphila* | 2 | 0.002 | 0.008 | Microbacteriaceae |  |
| Bacteria | 69373 | *Curtobacterium pusillum* | 2 | 0.002 | 0.008 | Microbacteriaceae |  |
| Bacteria | 192 | *Azospirillum brasilense* | 2 | 0.002 | 0.008 | Rhodospirillaceae |  |
| Bacteria | 1249552 | *Pseudohongiella spirulinae* | 2 | 0.002 | 0.008 | NO_NAME |  |
| Bacteria | 553239 | *Vibrio breoganii* | 2 | 0.002 | 0.008 | Vibrionaceae |  |
| Bacteria | 33959 | *Lactobacillus johnsonii* | 2 | 0.002 | 0.008 | Lactobacillaceae |  |
| Bacteria | 1402 | *Bacillus licheniformis* | 2 | 0.002 | 0.008 | Bacillaceae |  |
| Bacteria | 1405 | *Bacillus mycoides* | 2 | 0.002 | 0.008 | Bacillaceae |  |
| Bacteria | 305 | *Ralstonia solanacearum* | 2 | 0.002 | 0.008 | Burkholderiaceae |  |
| Bacteria | 165096 | *Weissella koreensis* | 2 | 0.002 | 0.008 | Leuconostocaceae |  |
| Bacteria | 339 | *Xanthomonas campestris* | 2 | 0.002 | 0.008 | Xanthomonadaceae |  |
| Bacteria | 1074467 | *Lactobacillus heilongjiangensis* | 2 | 0.002 | 0.008 | Lactobacillaceae |  |
| Bacteria | 1267600 | *Pantoea sp. IMH* | 2 | 0.002 | 0.008 | Erwiniaceae |  |
| Bacteria | 946333 | *Rhizobacter gummiphilus* | 2 | 0.002 | 0.008 | NO_NAME |  |
| Bacteria | 33074 | *Zymobacter palmae* | 2 | 0.002 | 0.008 | Halomonadaceae |  |
| Bacteria | 155077 | *Cellvibrio japonicus* | 2 | 0.002 | 0.008 | Cellvibrionaceae |  |
| Bacteria | 1922217 | *Candidatus Erwinia sp. ErCipseudotaxifoliae* | 2 | 0.002 | 0.008 | Erwiniaceae |  |
| Bacteria | 157779 | *Kushneria marisflavi* | 2 | 0.002 | 0.008 | Halomonadaceae |  |
| Bacteria | 1624 | *Lactobacillus salivarius* | 2 | 0.002 | 0.008 | Lactobacillaceae |  |
| Bacteria | 81475 | *Frateuria aurantia* | 2 | 0.002 | 0.008 | Rhodanobacteraceae |  |
| Bacteria | 33069 | *Pseudomonas viridiflava* | 2 | 0.002 | 0.008 | Pseudomonadaceae |  |
| Bacteria | 1933220 | *Sphingobacterium sp. B29* | 2 | 0.002 | 0.008 | Sphingobacteriaceae |  |
| Bacteria | 161398 | *Pseudoalteromonas phenolica* | 2 | 0.002 | 0.008 | Pseudoalteromonadaceae |  |
| Bacteria | 1399115 | *Exiguobacterium sp. MH3* | 2 | 0.002 | 0.008 | NO_NAME |  |
| Bacteria | 1296536 | *Enterobacter xiangfangensis* | 2 | 0.002 | 0.008 | Enterobacteriaceae |  |
| Bacteria | 392416 | *Lactobacillus crustorum* | 2 | 0.002 | 0.008 | Lactobacillaceae |  |
| Bacteria | 139208 | *Isoptericola variabilis* | 2 | 0.002 | 0.008 | Promicromonosporaceae |  |
| Bacteria | 1034889 | *Variovorax sp. HW608* | 2 | 0.002 | 0.008 | Comamonadaceae |  |
| Bacteria | 1967665 | *Colwellia sp. NB097-1* | 2 | 0.002 | 0.008 | Colwelliaceae |  |
| Bacteria | 1763536 | *Thalassotalea crassostreae* | 2 | 0.002 | 0.008 | Colwelliaceae |  |
| Bacteria | 1536772 | *Paenibacillus sp. FSL R7-0273* | 2 | 0.002 | 0.008 | Paenibacillaceae |  |
| Bacteria | 1710 | *Cellulosimicrobium cellulans* | 2 | 0.002 | 0.008 | Promicromonosporaceae |  |
| Bacteria | 82987 | *Tatumella ptyseos* | 2 | 0.002 | 0.008 | Erwiniaceae |  |
| Bacteria | 1194345 | *Candidatus Snodgrassella sp. TA1_30860* | 2 | 0.002 | 0.008 | Neisseriaceae |  |
| Bacteria | 582 | *Morganella morganii* | 2 | 0.002 | 0.008 | Morganellaceae |  |
| Bacteria | 32002 | *Achromobacter denitrificans* | 2 | 0.002 | 0.008 | Alcaligenaceae |  |
| Bacteria | 259059 | *Lactobacillus satsumensis* | 2 | 0.002 | 0.008 | Lactobacillaceae |  |
| Bacteria | 622 | *Shigella dysenteriae* | 2 | 0.002 | 0.008 | Enterobacteriaceae |  |
| Bacteria | 152331 | *Lactobacillus parabuchneri* | 2 | 0.002 | 0.008 | Lactobacillaceae |  |
| Bacteria | 1907766 | *Pseudomonas sp. BS-2016* | 2 | 0.002 | 0.008 | Pseudomonadaceae |  |
| Bacteria | 1505757 | *Siccibacter colletis* | 2 | 0.002 | 0.008 | Enterobacteriaceae |  |
| Bacteria | 202951 | *Acinetobacter bouvetii* | 2 | 0.002 | 0.008 | Moraxellaceae |  |
| Bacteria | 106590 | *Cupriavidus necator* | 2 | 0.002 | 0.008 | Burkholderiaceae |  |
| Bacteria | 55209 | *Pantoea cypripedii* | 2 | 0.002 | 0.008 | Erwiniaceae |  |
| Bacteria | 86304 | *Saccharophagus degradans* | 2 | 0.002 | 0.008 | Cellvibrionaceae |  |
| Bacteria | 750 | *Gallibacterium anatis* | 2 | 0.002 | 0.008 | Pasteurellaceae |  |
| Bacteria | 293371 | *Lactobacillus oligofermentans* | 2 | 0.002 | 0.008 | Lactobacillaceae |  |
| Bacteria | 654878 | *Acinetobacter sp. A3-6* | 2 | 0.002 | 0.008 | Moraxellaceae |  |
| Bacteria | 1178482 | *Halomonas huangheensis* | 2 | 0.002 | 0.008 | Halomonadaceae |  |
| Bacteria | 353 | *Azotobacter chroococcum* | 2 | 0.002 | 0.008 | Pseudomonadaceae |  |
| Bacteria | 82688 | *Lactobacillus nagelii* | 2 | 0.002 | 0.008 | Lactobacillaceae |  |
| Bacteria | 260364 | *Shewanella marisflavi* | 2 | 0.002 | 0.008 | Shewanellaceae |  |
| Bacteria | 1847728 | *Lactobacillus sp. WiKim39* | 2 | 0.002 | 0.008 | Lactobacillaceae |  |
| Bacteria | 158847 | *Megamonas hypermegale* | 2 | 0.002 | 0.008 | Selenomonadaceae |  |
| Bacteria | 240427 | *Lactobacillus paracollinoides* | 2 | 0.002 | 0.008 | Lactobacillaceae |  |
| Bacteria | 691 | *Vibrio natriegens* | 2 | 0.002 | 0.008 | Vibrionaceae |  |
| Bacteria | 208224 | *Enterobacter kobei* | 2 | 0.002 | 0.008 | Enterobacteriaceae |  |
| Bacteria | 386891 | *Moraxella bovoculi* | 2 | 0.002 | 0.008 | Moraxellaceae |  |
| Bacteria | 920 | *Acidithiobacillus ferrooxidans* | 2 | 0.002 | 0.008 | Acidithiobacillaceae |  |
| Bacteria | 927 | *Halothiobacillus neapolitanus* | 2 | 0.002 | 0.008 | Halothiobacillaceae |  |
| Bacteria | 1674922 | *Labrenzia sp. CP4* | 2 | 0.002 | 0.008 | Rhodobacteraceae |  |
| Bacteria | 316 | *Pseudomonas stutzeri* | 2 | 0.002 | 0.008 | Pseudomonadaceae |  |
| Bacteria | 85698 | *Achromobacter xylosoxidans* | 2 | 0.002 | 0.008 | Alcaligenaceae |  |
| Bacteria | 45610 | *Psychrobacter urativorans* | 2 | 0.002 | 0.008 | Moraxellaceae |  |
| Bacteria | 2751 | *Carnobacterium maltaromaticum* | 2 | 0.002 | 0.008 | Carnobacteriaceae |  |
| Bacteria | 104099 | *Acetobacter orleanensis* | 2 | 0.002 | 0.008 | Acetobacteraceae |  |
| Bacteria | 480813 | *Tatumella saanichensis* | 2 | 0.002 | 0.008 | Erwiniaceae |  |
| Bacteria | 234831 | *Pseudoalteromonas sp. SM9913* | 2 | 0.002 | 0.008 | Pseudoalteromonadaceae |  |
| Bacteria | 731 | *Histophilus somni* | 2 | 0.002 | 0.008 | Pasteurellaceae |  |
| Bacteria | 1536769 | *Paenibacillus sp. FSL P4-0081* | 2 | 0.002 | 0.008 | Paenibacillaceae |  |
| Bacteria | 2045 | *Pimelobacter simplex* | 2 | 0.002 | 0.008 | Nocardioidaceae |  |
| Bacteria | 1549858 | *Sphingomonas taxi* | 2 | 0.002 | 0.008 | Sphingomonadaceae |  |
| Bacteria | 55210 | *Brenneria nigrifluens* | 2 | 0.002 | 0.008 | Pectobacteriaceae |  |
| Bacteria | 1707785 | *Massilia sp. WG5* | 2 | 0.002 | 0.008 | Oxalobacteraceae |  |
| Bacteria | 42862 | *Rickettsia felis* | 2 | 0.002 | 0.008 | Rickettsiaceae |  |
| Bacteria | 106648 | *Acinetobacter bereziniae* | 2 | 0.002 | 0.008 | Moraxellaceae |  |
| Bacteria | 1604 | *Lactobacillus amylovorus* | 2 | 0.002 | 0.008 | Lactobacillaceae |  |
| Bacteria | 988805 | *Enterobacteriaceae bacterium 8NS7* | 2 | 0.002 | 0.008 | Enterobacteriaceae |  |
| Bacteria | 988801 | *Rosenbergiella nectarea* | 2 | 0.002 | 0.008 | Enterobacteriaceae |  |
| Bacteria | 358 | *Agrobacterium tumefaciens* | 2 | 0.002 | 0.008 | Rhizobiaceae |  |
| Bacteria | 178901 | *Acetobacter malorum* | 2 | 0.002 | 0.008 | Acetobacteraceae |  |
| Bacteria | 212522 | *Pseudomonas sp. KNA6-3* | 2 | 0.002 | 0.008 | Pseudomonadaceae |  |
| Bacteria | 69220 | *Lelliottia nimipressuralis* | 2 | 0.002 | 0.008 | Enterobacteriaceae |  |
| Bacteria | 280 | *Xanthobacter autotrophicus* | 2 | 0.002 | 0.008 | Xanthobacteraceae |  |
| Bacteria | 1653480 | *Alloactinosynnema sp. L-07* | 2 | 0.002 | 0.008 | Pseudonocardiaceae |  |
| Bacteria | 94132 | *Ramlibacter tataouinensis* | 2 | 0.002 | 0.008 | Comamonadaceae |  |
| Bacteria | 253 | *Chryseobacterium indologenes* | 2 | 0.002 | 0.008 | Flavobacteriaceae |  |
| Bacteria | 1526571 | *Lacimicrobium alkaliphilum* | 2 | 0.002 | 0.008 | Alteromonadaceae |  |
| Bacteria | 1254 | *Pediococcus acidilactici* | 2 | 0.002 | 0.008 | Lactobacillaceae |  |
| Bacteria | 85404 | *Mannheimia varigena* | 2 | 0.002 | 0.008 | Pasteurellaceae |  |
| Bacteria | 1423 | *Bacillus subtilis* | 2 | 0.002 | 0.008 | Bacillaceae |  |
| Bacteria | 533 | *Beijerinckia indica* | 2 | 0.002 | 0.008 | Beijerinckiaceae |  |
| Bacteria | 1938604 | *Aquaspirillum sp. LM1* | 2 | 0.002 | 0.008 | Chromobacteriaceae |  |
| Bacteria | 397260 | *Sphingomonas sanxanigenens* | 2 | 0.002 | 0.008 | Sphingomonadaceae |  |
| Bacteria | 28450 | *Burkholderia pseudomallei* | 2 | 0.002 | 0.008 | Burkholderiaceae |  |
| Bacteria | 663 | *Vibrio alginolyticus* | 2 | 0.002 | 0.008 | Vibrionaceae |  |
| Bacteria | 158899 | *Collimonas fungivorans* | 2 | 0.002 | 0.008 | Oxalobacteraceae |  |
| Bacteria | 593057 | *Enterobacter sp. ATCC 27981* | 2 | 0.002 | 0.008 | Enterobacteriaceae |  |
| Bacteria | 251701 | *Pseudomonas syringae group genomosp. 3* | 2 | 0.002 | 0.008 | Pseudomonadaceae |  |
| Bacteria | 123899 | *Bordetella trematum* | 2 | 0.002 | 0.008 | Alcaligenaceae |  |
| Bacteria | 104623 | *Serratia sp. ATCC 39006* | 2 | 0.002 | 0.008 | Yersiniaceae |  |
| Bacteria | 246432 | *Staphylococcus equorum* | 2 | 0.002 | 0.008 | Staphylococcaceae |  |
| Bacteria | 76761 | *Pseudomonas veronii* | 2 | 0.002 | 0.008 | Pseudomonadaceae |  |
| Bacteria | 267363 | *Lactobacillus zymae* | 2 | 0.002 | 0.008 | Lactobacillaceae |  |
| Bacteria | 507626 | *Halomonas chromatireducens* | 2 | 0.002 | 0.008 | Halomonadaceae |  |
| Bacteria | 138073 | *Candidatus Regiella insecticola* | 2 | 0.002 | 0.008 | Enterobacteriaceae |  |
| Bacteria | 1070130 | *Candidatus Gullanella endobia* | 2 | 0.002 | 0.008 | Enterobacteriaceae |  |
| Bacteria | 60478 | *Shewanella amazonensis* | 2 | 0.002 | 0.008 | Shewanellaceae |  |
| Bacteria | 712633 | *Streptococcus sp. oral taxon 431* | 1 | 0.001 | 0.004 | Streptococcaceae |  |
| Bacteria | 1349819 | *Aureimonas sp. AU20* | 1 | 0.001 | 0.004 | Aurantimonadaceae |  |
| Bacteria | 1792307 | *Bosea sp. PAMC 26642* | 1 | 0.001 | 0.004 | Bradyrhizobiaceae |  |
| Bacteria | 404011 | *Shewanella piezotolerans* | 1 | 0.001 | 0.004 | Shewanellaceae |  |
| Bacteria | 632955 | *Acinetobacter rudis* | 1 | 0.001 | 0.004 | Moraxellaceae |  |
| Bacteria | 354356 | *Niastella koreensis* | 1 | 0.001 | 0.004 | Chitinophagaceae |  |
| Bacteria | 28109 | *Pseudoalteromonas nigrifaciens* | 1 | 0.001 | 0.004 | Pseudoalteromonadaceae |  |
| Bacteria | 1470434 | *Zhongshania aliphaticivorans* | 1 | 0.001 | 0.004 | Spongiibacteraceae |  |
| Bacteria | 37919 | *Rhodococcus opacus* | 1 | 0.001 | 0.004 | Nocardiaceae |  |
| Bacteria | 1638161 | *Aureimonas sp. AU12* | 1 | 0.001 | 0.004 | Aurantimonadaceae |  |
| Bacteria | 1493 | *Clostridium cellulovorans* | 1 | 0.001 | 0.004 | Clostridiaceae |  |
| Bacteria | 703222 | *Kibdelosporangium sp. MJ126-NF4* | 1 | 0.001 | 0.004 | Pseudonocardiaceae |  |
| Bacteria | 1636152 | *Planctomyces sp. SH-PL62* | 1 | 0.001 | 0.004 | Planctomycetaceae |  |
| Bacteria | 1708 | *Cellulomonas fimi* | 1 | 0.001 | 0.004 | Cellulomonadaceae |  |
| Bacteria | 47885 | *Pseudomonas oryzihabitans* | 1 | 0.001 | 0.004 | Pseudomonadaceae |  |
| Bacteria | 657310 | *Enterococcus sp. 7L76* | 1 | 0.001 | 0.004 | Enterococcaceae |  |
| Bacteria | 1250539 | *Pelagibaca abyssi* | 1 | 0.001 | 0.004 | Rhodobacteraceae |  |
| Bacteria | 296 | *Pseudomonas fragi* | 1 | 0.001 | 0.004 | Pseudomonadaceae |  |
| Bacteria | 293 | *Brevundimonas diminuta* | 1 | 0.001 | 0.004 | Caulobacteraceae |  |
| Bacteria | 43771 | *Corynebacterium urealyticum* | 1 | 0.001 | 0.004 | Corynebacteriaceae |  |
| Bacteria | 880157 | *Xenorhabdus khoisanae* | 1 | 0.001 | 0.004 | Morganellaceae |  |
| Bacteria | 405212 | *Alicyclobacillus acidocaldarius* | 1 | 0.001 | 0.004 | Alicyclobacillaceae |  |
| Bacteria | 28035 | *Staphylococcus lugdunensis* | 1 | 0.001 | 0.004 | Staphylococcaceae |  |
| Bacteria | 28038 | *Lactobacillus curvatus* | 1 | 0.001 | 0.004 | Lactobacillaceae |  |
| Bacteria | 285665 | *Micromonospora coriariae* | 1 | 0.001 | 0.004 | Micromonosporaceae |  |
| Bacteria | 640512 | *Burkholderia sp. CCGE1003* | 1 | 0.001 | 0.004 | Burkholderiaceae |  |
| Bacteria | 1985873 | *Sulfuriferula sp. AH1* | 1 | 0.001 | 0.004 | Gallionellaceae |  |
| Bacteria | 47763 | *Streptomyces lydicus* | 1 | 0.001 | 0.004 | Streptomycetaceae |  |
| Bacteria | 1871034 | *Propionimicrobium sp. Marseille-P3275* | 1 | 0.001 | 0.004 | Propionibacteriaceae |  |
| Bacteria | 437505 | *Granulosicoccus antarcticus* | 1 | 0.001 | 0.004 | Granulosicoccaceae |  |
| Bacteria | 86662 | *Bacillus weihenstephanensis* | 1 | 0.001 | 0.004 | Bacillaceae |  |
| Bacteria | 1094342 | *Alcanivorax xenomutans* | 1 | 0.001 | 0.004 | Alcanivoracaceae |  |
| Bacteria | 91624 | *Clostridium acidisoli* | 1 | 0.001 | 0.004 | Clostridiaceae |  |
| Bacteria | 168169 | *Candidatus Ishikawaella capsulata* | 1 | 0.001 | 0.004 | Enterobacteriaceae |  |
| Bacteria | 1838286 | *Lacunisphaera limnophila* | 1 | 0.001 | 0.004 | Opitutaceae |  |
| Bacteria | 337 | *Burkholderia glumae* | 1 | 0.001 | 0.004 | Burkholderiaceae |  |
| Bacteria | 193462 | *Streptomyces niveus* | 1 | 0.001 | 0.004 | Streptomycetaceae |  |
| Bacteria | 1470176 | *Actinoalloteichus hoggarensis* | 1 | 0.001 | 0.004 | Pseudonocardiaceae |  |
| Bacteria | 346 | *Xanthomonas citri* | 1 | 0.001 | 0.004 | Xanthomonadaceae |  |
| Bacteria | 164348 | *Streptomyces puniciscabiei* | 1 | 0.001 | 0.004 | Streptomycetaceae |  |
| Bacteria | 1850250 | *Rhodobacter sp. LPB0142* | 1 | 0.001 | 0.004 | Rhodobacteraceae |  |
| Bacteria | 1218506 | *Lactobacillus kimbladii* | 1 | 0.001 | 0.004 | Lactobacillaceae |  |
| Bacteria | 80878 | *Acidovorax temperans* | 1 | 0.001 | 0.004 | Comamonadaceae |  |
| Bacteria | 1141883 | *Massilia putida* | 1 | 0.001 | 0.004 | Oxalobacteraceae |  |
| Bacteria | 169760 | *Paenibacillus stellifer* | 1 | 0.001 | 0.004 | Paenibacillaceae |  |
| Bacteria | 208439 | *Amycolatopsis japonica* | 1 | 0.001 | 0.004 | Pseudonocardiaceae |  |
| Bacteria | 245188 | *Loktanella vestfoldensis* | 1 | 0.001 | 0.004 | Rhodobacteraceae |  |
| Bacteria | 252514 | *Microbulbifer thermotolerans* | 1 | 0.001 | 0.004 | Microbulbiferaceae |  |
| Bacteria | 332055 | *Sphingobium indicum* | 1 | 0.001 | 0.004 | Sphingomonadaceae |  |
| Bacteria | 81476 | *Pluralibacter pyrinus* | 1 | 0.001 | 0.004 | Enterobacteriaceae |  |
| Bacteria | 1410383 | *Candidatus Tachikawaea gelatinosa* | 1 | 0.001 | 0.004 | Enterobacteriaceae |  |
| Bacteria | 1561023 | *Curtobacterium sp. MR_MD2014* | 1 | 0.001 | 0.004 | Microbacteriaceae |  |
| Bacteria | 82979 | *Budvicia aquatica* | 1 | 0.001 | 0.004 | Budviciaceae |  |
| Bacteria | 312306 | *Pseudomonas entomophila* | 1 | 0.001 | 0.004 | Pseudomonadaceae |  |
| Bacteria | 33910 | *Amycolatopsis mediterranei* | 1 | 0.001 | 0.004 | Pseudonocardiaceae |  |
| Bacteria | 472705 | *Pantoea conspicua* | 1 | 0.001 | 0.004 | Erwiniaceae |  |
| Bacteria | 1416627 | *Oceanisphaera profunda* | 1 | 0.001 | 0.004 | Aeromonadaceae |  |
| Bacteria | 1631871 | *Weissella jogaejeotgali* | 1 | 0.001 | 0.004 | Leuconostocaceae |  |
| Bacteria | 1936081 | *Seonamhaeicola sp. S2-3* | 1 | 0.001 | 0.004 | Flavobacteriaceae |  |
| Bacteria | 1497809 | *Klebsiella sp. 6837* | 1 | 0.001 | 0.004 | Enterobacteriaceae |  |
| Bacteria | 207513 | *symbiont of Wahlgreniella nervata* | 1 | 0.001 | 0.004 | Enterobacteriaceae |  |
| Bacteria | 314275 | *Alteromonas mediterranea* | 1 | 0.001 | 0.004 | Alteromonadaceae |  |
| Bacteria | 129817 | *Pseudomonas brenneri* | 1 | 0.001 | 0.004 | Pseudomonadaceae |  |
| Bacteria | 1945512 | *Cellvibrio sp. PSBB023* | 1 | 0.001 | 0.004 | Cellvibrionaceae |  |
| Bacteria | 670052 | *Cryobacterium arcticum* | 1 | 0.001 | 0.004 | Microbacteriaceae |  |
| Bacteria | 1888 | *Streptomyces albus* | 1 | 0.001 | 0.004 | Streptomycetaceae |  |
| Bacteria | 40567 | *Actinosynnema mirum* | 1 | 0.001 | 0.004 | Pseudonocardiaceae |  |
| Bacteria | 447034 | *Rickettsia endosymbiont of Crassostrea ariakensis* | 1 | 0.001 | 0.004 | Rickettsiaceae |  |
| Bacteria | 2123 | *Mycoplasma putrefaciens* | 1 | 0.001 | 0.004 | Mycoplasmataceae |  |
| Bacteria | 1286 | *Staphylococcus simulans* | 1 | 0.001 | 0.004 | Staphylococcaceae |  |
| Bacteria | 1282 | *Staphylococcus epidermidis* | 1 | 0.001 | 0.004 | Staphylococcaceae |  |
| Bacteria | 1288 | *Staphylococcus xylosus* | 1 | 0.001 | 0.004 | Staphylococcaceae |  |
| Bacteria | 748280 | *Pseudogulbenkiania sp. NH8B* | 1 | 0.001 | 0.004 | Chromobacteriaceae |  |
| Bacteria | 643674 | *Paenalcaligenes hominis* | 1 | 0.001 | 0.004 | Alcaligenaceae |  |
| Bacteria | 58138 | *Desulfotomaculum acetoxidans* | 1 | 0.001 | 0.004 | Peptococcaceae |  |
| Bacteria | 1303 | *Streptococcus oralis* | 1 | 0.001 | 0.004 | Streptococcaceae |  |
| Bacteria | 1108595 | *Chromobacterium vaccinii* | 1 | 0.001 | 0.004 | Chromobacteriaceae |  |
| Bacteria | 134533 | *Acinetobacter parvus* | 1 | 0.001 | 0.004 | Moraxellaceae |  |
| Bacteria | 75588 | *Pseudomonas libanensis* | 1 | 0.001 | 0.004 | Pseudomonadaceae |  |
| Bacteria | 589873 | *Alteromonas australica* | 1 | 0.001 | 0.004 | Alteromonadaceae |  |
| Bacteria | 2015316 | *Bosea sp. AS-1* | 1 | 0.001 | 0.004 | Bradyrhizobiaceae |  |
| Bacteria | 24 | *Shewanella putrefaciens* | 1 | 0.001 | 0.004 | Shewanellaceae |  |
| Bacteria | 1416806 | *Bordetella genomosp. 8* | 1 | 0.001 | 0.004 | Alcaligenaceae |  |
| Bacteria | 645517 | *Altererythrobacter namhicola* | 1 | 0.001 | 0.004 | Erythrobacteraceae |  |
| Bacteria | 121292 | *Pseudarthrobacter sulfonivorans* | 1 | 0.001 | 0.004 | Micrococcaceae |  |
| Bacteria | 1920191 | *Delftia sp. HK171* | 1 | 0.001 | 0.004 | Comamonadaceae |  |
| Bacteria | 629680 | *Brevibacterium sandarakinum* | 1 | 0.001 | 0.004 | Brevibacteriaceae |  |
| Bacteria | 2746 | *Halomonas elongata* | 1 | 0.001 | 0.004 | Halomonadaceae |  |
| Bacteria | 380021 | *Pseudomonas protegens* | 1 | 0.001 | 0.004 | Pseudomonadaceae |  |
| Bacteria | 1746743 | *Microbacterium sp. HBUM178421* | 1 | 0.001 | 0.004 | Microbacteriaceae |  |
| Bacteria | 1384589 | *Erwinia teleogrylli* | 1 | 0.001 | 0.004 | Erwiniaceae |  |
| Bacteria | 43263 | *Pseudomonas alcaligenes* | 1 | 0.001 | 0.004 | Pseudomonadaceae |  |
| Bacteria | 1987723 | *Cellvibrio sp. PSBB006* | 1 | 0.001 | 0.004 | Cellvibrionaceae |  |
| Bacteria | 53412 | *Pseudomonas resinovorans* | 1 | 0.001 | 0.004 | Pseudomonadaceae |  |
| Bacteria | 1620215 | *Sulfuricaulis limicola* | 1 | 0.001 | 0.004 | Acidiferrobacteraceae |  |
| Bacteria | 715451 | *Alteromonas naphthalenivorans* | 1 | 0.001 | 0.004 | Alteromonadaceae |  |
| Bacteria | 1771 | *Mycobacterium phlei* | 1 | 0.001 | 0.004 | Mycobacteriaceae |  |
| Bacteria | 1116375 | *Vibrio sp. EJY3* | 1 | 0.001 | 0.004 | Vibrionaceae |  |
| Bacteria | 1353507 | *Xenorhabdus sp. 157-C* | 1 | 0.001 | 0.004 | Morganellaceae |  |
| Bacteria | 1063 | *Rhodobacter sphaeroides* | 1 | 0.001 | 0.004 | Rhodobacteraceae |  |
| Bacteria | 376 | *Bradyrhizobium sp.* | 1 | 0.001 | 0.004 | Bradyrhizobiaceae |  |
| Bacteria | 758802 | *Mycobacterium litorale* | 1 | 0.001 | 0.004 | Mycobacteriaceae |  |
| Bacteria | 74829 | *Pseudomonas balearica* | 1 | 0.001 | 0.004 | Pseudomonadaceae |  |
| Bacteria | 1505596 | *Blochmannia endosymbiont of Polyrhachis (Hedomyrma) turneri* | 1 | 0.001 | 0.004 | Enterobacteriaceae |  |
| Bacteria | 1335757 | *Spiribacter curvatus* | 1 | 0.001 | 0.004 | Ectothiorhodospiraceae |  |
| Bacteria | 80842 | *Herbaspirillum rubrisubalbicans* | 1 | 0.001 | 0.004 | Oxalobacteraceae |  |
| Bacteria | 472175 | *Nitratireductor basaltis* | 1 | 0.001 | 0.004 | Phyllobacteriaceae |  |
| Bacteria | 414778 | *Planococcus donghaensis* | 1 | 0.001 | 0.004 | Planococcaceae |  |
| Bacteria | 649841 | *Sulfuricella denitrificans* | 1 | 0.001 | 0.004 | Gallionellaceae |  |
| Bacteria | 1778874 | *Klebsiella sp. T4-1* | 1 | 0.001 | 0.004 | Enterobacteriaceae |  |
| Bacteria | 1437774 | *Salinicoccus sp. BAB 3246* | 1 | 0.001 | 0.004 | Staphylococcaceae |  |
| Bacteria | 65741 | *Pseudomonas knackmussii* | 1 | 0.001 | 0.004 | Pseudomonadaceae |  |
| Bacteria | 1276 | *Kytococcus sedentarius* | 1 | 0.001 | 0.004 | Dermacoccaceae |  |
| Bacteria | 1546149 | *Diaphorobacter polyhydroxybutyrativorans* | 1 | 0.001 | 0.004 | Comamonadaceae |  |
| Bacteria | 1882682 | *Microvirga ossetica* | 1 | 0.001 | 0.004 | Methylobacteriaceae |  |
| Bacteria | 1906741 | *Jeongeupia sp. USM3* | 1 | 0.001 | 0.004 | Chromobacteriaceae |  |
| Bacteria | 29438 | *Pseudomonas savastanoi* | 1 | 0.001 | 0.004 | Pseudomonadaceae |  |
| Bacteria | 72000 | *Kocuria rhizophila* | 1 | 0.001 | 0.004 | Micrococcaceae |  |
| Bacteria | 242606 | *Luteibacter rhizovicinus* | 1 | 0.001 | 0.004 | Rhodanobacteraceae |  |
| Bacteria | 238834 | *Clostridium estertheticum* | 1 | 0.001 | 0.004 | Clostridiaceae |  |
| Bacteria | 623 | *Shigella flexneri* | 1 | 0.001 | 0.004 | Enterobacteriaceae |  |
| Bacteria | 79880 | *Bacillus clausii* | 1 | 0.001 | 0.004 | Bacillaceae |  |
| Bacteria | 134676 | *Actinoplanes sp. SE50/110* | 1 | 0.001 | 0.004 | Micromonosporaceae |  |
| Bacteria | 1812101 | *Coetzeea brasiliensis* | 1 | 0.001 | 0.004 | Thorselliaceae |  |
| Bacteria | 109790 | *Lactobacillus jensenii* | 1 | 0.001 | 0.004 | Lactobacillaceae |  |
| Bacteria | 652 | *Aeromonas schubertii* | 1 | 0.001 | 0.004 | Aeromonadaceae |  |
| Bacteria | 1505 | *Paeniclostridium sordellii* | 1 | 0.001 | 0.004 | Peptostreptococcaceae |  |
| Bacteria | 1500686 | *Pseudomonas sp. Os17* | 1 | 0.001 | 0.004 | Pseudomonadaceae |  |
| Bacteria | 1599 | *Lactobacillus sakei* | 1 | 0.001 | 0.004 | Lactobacillaceae |  |
| Bacteria | 248026 | *Cupriavidus pinatubonensis* | 1 | 0.001 | 0.004 | Burkholderiaceae |  |
| Bacteria | 247523 | *Pseudoalteromonas aliena* | 1 | 0.001 | 0.004 | Pseudoalteromonadaceae |  |
| Bacteria | 399969 | *Tatumella punctata* | 1 | 0.001 | 0.004 | Erwiniaceae |  |
| Bacteria | 415229 | *Pseudoxanthomonas spadix* | 1 | 0.001 | 0.004 | Xanthomonadaceae |  |
| Bacteria | 202955 | *Acinetobacter tjernbergiae* | 1 | 0.001 | 0.004 | Moraxellaceae |  |
| Bacteria | 202950 | *Acinetobacter baylyi* | 1 | 0.001 | 0.004 | Moraxellaceae |  |
| Bacteria | 202952 | *Acinetobacter gerneri* | 1 | 0.001 | 0.004 | Moraxellaceae |  |
| Bacteria | 106592 | *Ensifer adhaerens* | 1 | 0.001 | 0.004 | Rhizobiaceae |  |
| Bacteria | 319236 | *Nonlabens sediminis* | 1 | 0.001 | 0.004 | Flavobacteriaceae |  |
| Bacteria | 69328 | *Pseudomonas sp. VLB120* | 1 | 0.001 | 0.004 | Pseudomonadaceae |  |
| Bacteria | 483199 | *Acetobacter fabarum* | 1 | 0.001 | 0.004 | Acetobacteraceae |  |
| Bacteria | 867805 | *bacterium P2-10-1* | 1 | 0.001 | 0.004 | NO_NAME |  |
| Bacteria | 83263 | *Aminobacter aminovorans* | 1 | 0.001 | 0.004 | Phyllobacteriaceae |  |
| Bacteria | 44255 | *Bradyrhizobium oligotrophicum* | 1 | 0.001 | 0.004 | Bradyrhizobiaceae |  |
| Bacteria | 1471 | *Bacillus methanolicus* | 1 | 0.001 | 0.004 | Bacillaceae |  |
| Bacteria | 1690248 | *Arthrobacter sp. LS16* | 1 | 0.001 | 0.004 | Micrococcaceae |  |
| Bacteria | 40318 | *Streptomyces nodosus* | 1 | 0.001 | 0.004 | Streptomycetaceae |  |
| Bacteria | 292913 | *Sphingopyxis sp. 113P3* | 1 | 0.001 | 0.004 | Sphingomonadaceae |  |
| Bacteria | 348824 | *Rhizobium favelukesii* | 1 | 0.001 | 0.004 | Rhizobiaceae |  |
| Bacteria | 1462996 | *Paenibacillus yonginensis* | 1 | 0.001 | 0.004 | Paenibacillaceae |  |
| Bacteria | 1144748 | *Kangiella sediminilitoris* | 1 | 0.001 | 0.004 | Kangiellaceae |  |
| Bacteria | 576611 | *Polynucleobacter asymbioticus* | 1 | 0.001 | 0.004 | Burkholderiaceae |  |
| Bacteria | 748811 | *Sulfuritalea hydrogenivorans* | 1 | 0.001 | 0.004 | Sterolibacteriaceae |  |
| Bacteria | 2331 | *Halanaerobium praevalens* | 1 | 0.001 | 0.004 | Halanaerobiaceae |  |
| Bacteria | 95485 | *Burkholderia stabilis* | 1 | 0.001 | 0.004 | Burkholderiaceae |  |
| Bacteria | 300231 | *Glaciecola nitratireducens* | 1 | 0.001 | 0.004 | Alteromonadaceae |  |
| Bacteria | 1689 | *Bifidobacterium dentium* | 1 | 0.001 | 0.004 | Bifidobacteriaceae |  |
| Bacteria | 1432056 | *Mannheimia sp. USDA-ARS-USMARC-1261* | 1 | 0.001 | 0.004 | Pasteurellaceae |  |
| Bacteria | 1712675 | *Turicibacter sp. H121* | 1 | 0.001 | 0.004 | Erysipelotrichaceae |  |
| Bacteria | 1541173 | *bacterium NJ6* | 1 | 0.001 | 0.004 | NO_NAME |  |
| Bacteria | 279113 | *Collimonas pratensis* | 1 | 0.001 | 0.004 | Oxalobacteraceae |  |
| Bacteria | 279058 | *Collimonas arenae* | 1 | 0.001 | 0.004 | Oxalobacteraceae |  |
| Bacteria | 43675 | *Rothia mucilaginosa* | 1 | 0.001 | 0.004 | Micrococcaceae |  |
| Bacteria | 1915 | *Streptomyces lincolnensis* | 1 | 0.001 | 0.004 | Streptomycetaceae |  |
| Bacteria | 1548 | *Clostridium scatologenes* | 1 | 0.001 | 0.004 | Clostridiaceae |  |
| Bacteria | 1805827 | *Rhodococcus sp. MTM3W5.2* | 1 | 0.001 | 0.004 | Nocardiaceae |  |
| Bacteria | 1465 | *Brevibacillus laterosporus* | 1 | 0.001 | 0.004 | Paenibacillaceae |  |
| Bacteria | 1659194 | *Pseudomonas sp. GR 6-02* | 1 | 0.001 | 0.004 | Pseudomonadaceae |  |
| Bacteria | 134534 | *Acinetobacter gyllenbergii* | 1 | 0.001 | 0.004 | Moraxellaceae |  |
| Bacteria | 761 | *Pasteurella testudinis* | 1 | 0.001 | 0.004 | Pasteurellaceae |  |
| Bacteria | 1313 | *Streptococcus pneumoniae* | 1 | 0.001 | 0.004 | Streptococcaceae |  |
| Bacteria | 1834196 | *Lachnoclostridium sp. YL32* | 1 | 0.001 | 0.004 | Lachnospiraceae |  |
| Bacteria | 1549889 | *Thorsellia kandunguensis* | 1 | 0.001 | 0.004 | Thorselliaceae |  |
| Bacteria | 1978339 | *Chromatiaceae bacterium 2141T.STBD.0c.01a* | 1 | 0.001 | 0.004 | Chromatiaceae |  |
| Bacteria | 1871111 | *Acinetobacter defluvii* | 1 | 0.001 | 0.004 | Moraxellaceae |  |
| Bacteria | 94122 | *Shewanella sp. ANA-3* | 1 | 0.001 | 0.004 | Shewanellaceae |  |
| Bacteria | 190895 | *Vibrio rotiferianus* | 1 | 0.001 | 0.004 | Vibrionaceae |  |
| Bacteria | 1798223 | *Leifsonia sp. 21MFCrub1.1* | 1 | 0.001 | 0.004 | Microbacteriaceae |  |
| Bacteria | 1828 | *Rhodococcus fascians* | 1 | 0.001 | 0.004 | Nocardiaceae |  |
| Bacteria | 477641 | *Modestobacter marinus* | 1 | 0.001 | 0.004 | Geodermatophilaceae |  |
| Bacteria | 921 | *Starkeya novella* | 1 | 0.001 | 0.004 | Xanthobacteraceae |  |
| Bacteria | 183795 | *Pseudomonas mediterranea* | 1 | 0.001 | 0.004 | Pseudomonadaceae |  |
| Bacteria | 349106 | *Psychrobacter sp. PRwf-1* | 1 | 0.001 | 0.004 | Moraxellaceae |  |
| Bacteria | 169669 | *Pseudomonas extremorientalis* | 1 | 0.001 | 0.004 | Pseudomonadaceae |  |
| Bacteria | 1160097 | *Pasteurellaceae bacterium BHMR2* | 1 | 0.001 | 0.004 | Pasteurellaceae |  |
| Bacteria | 680490 | *Dickeya sp. CITA_Q5* | 1 | 0.001 | 0.004 | Pectobacteriaceae |  |
| Bacteria | 187137 | *Oceanithermus profundus* | 1 | 0.001 | 0.004 | Thermaceae |  |
| Bacteria | 46677 | *Pseudomonas agarici* | 1 | 0.001 | 0.004 | Pseudomonadaceae |  |
| Bacteria | 198107 | *Azoarcus sp. CIB* | 1 | 0.001 | 0.004 | Zoogloeaceae |  |
| Bacteria | 1685010 | *Chryseobacterium sp. IHB B 10212* | 1 | 0.001 | 0.004 | Flavobacteriaceae |  |
| Bacteria | 1962264 | *Halomonas sp. 'Soap Lake #7'* | 1 | 0.001 | 0.004 | Halomonadaceae |  |
| Bacteria | 1007676 | *Lactobacillus ginsenosidimutans* | 1 | 0.001 | 0.004 | Lactobacillaceae |  |
| Bacteria | 1442136 | *Ectothiorhodospira sp. BSL-9* | 1 | 0.001 | 0.004 | Ectothiorhodospiraceae |  |
| Bacteria | 649534 | *alpha proteobacterium D323* | 1 | 0.001 | 0.004 | NO_NAME |  |
| Bacteria | 70864 | *Shewanella pealeana* | 1 | 0.001 | 0.004 | Shewanellaceae |  |
| Bacteria | 80854 | *Moritella viscosa* | 1 | 0.001 | 0.004 | Moritellaceae |  |
| Bacteria | 1644893 | *Enterobacter sp. UIWRF1401* | 1 | 0.001 | 0.004 | Enterobacteriaceae |  |
| Bacteria | 1640 | *Listeria seeligeri* | 1 | 0.001 | 0.004 | Listeriaceae |  |
| Bacteria | 1727 | *Corynebacterium variabile* | 1 | 0.001 | 0.004 | Corynebacteriaceae |  |
| Bacteria | 936476 | *Marinomonas posidonica* | 1 | 0.001 | 0.004 | Oceanospirillaceae |  |
| Bacteria | 1136497 | *Brevibacterium siliguriense* | 1 | 0.001 | 0.004 | Brevibacteriaceae |  |
| Bacteria | 1211326 | *Spirosoma aerolatum* | 1 | 0.001 | 0.004 | Cytophagaceae |  |
| Bacteria | 645 | *Aeromonas salmonicida* | 1 | 0.001 | 0.004 | Aeromonadaceae |  |
| Bacteria | 732 | *Aggregatibacter aphrophilus* | 1 | 0.001 | 0.004 | Pasteurellaceae |  |
| Bacteria | 471223 | *Geobacillus sp. WCH70* | 1 | 0.001 | 0.004 | Bacillaceae |  |
| Bacteria | 60481 | *Shewanella sp. MR-7* | 1 | 0.001 | 0.004 | Shewanellaceae |  |
| Bacteria | 60480 | *Shewanella sp. MR-4* | 1 | 0.001 | 0.004 | Shewanellaceae |  |
| Bacteria | 92932 | *Pantoea cedenensis* | 1 | 0.001 | 0.004 | Erwiniaceae |  |
| Bacteria | 75985 | *Mannheimia haemolytica* | 1 | 0.001 | 0.004 | Pasteurellaceae |  |
| Bacteria | 1653478 | *Rhodococcus sp. PBTS 1* | 1 | 0.001 | 0.004 | Nocardiaceae |  |
| Bacteria | 240495 | *Pseudonocardia dioxanivorans* | 1 | 0.001 | 0.004 | Pseudonocardiaceae |  |
| Bacteria | 410330 | *Calyptogena okutanii thioautotrophic gill symbiont* | 1 | 0.001 | 0.004 | NO_NAME |  |
| Bacteria | 351614 | *Xenorhabdus stockiae* | 1 | 0.001 | 0.004 | Morganellaceae |  |
| Bacteria | 1792508 | *Yangia sp. CCB-MM3* | 1 | 0.001 | 0.004 | Rhodobacteraceae |  |
| Bacteria | 1584 | *Lactobacillus delbrueckii* | 1 | 0.001 | 0.004 | Lactobacillaceae |  |
| Bacteria | 1778263 | *Candidatus Hoaglandella endobia* | 1 | 0.001 | 0.004 | Enterobacteriaceae |  |
| Bacteria | 1778264 | *Candidatus Mikella endobia* | 1 | 0.001 | 0.004 | Enterobacteriaceae |  |
| Bacteria | 52242 | *Lactobacillus gallinarum* | 1 | 0.001 | 0.004 | Lactobacillaceae |  |
| Bacteria | 634113 | *Arsenophonus symbiont of Lipoptena fortisetosa* | 1 | 0.001 | 0.004 | Morganellaceae |  |
| Bacteria | 2426 | *Teredinibacter turnerae* | 1 | 0.001 | 0.004 | Cellvibrionaceae |  |
| Bacteria | 394958 | *Clostridium taeniosporum* | 1 | 0.001 | 0.004 | Clostridiaceae |  |
| Bacteria | 1658672 | *Ottowia sp. oral taxon 894* | 1 | 0.001 | 0.004 | Comamonadaceae |  |
| Bacteria | 1658671 | *Arsenicicoccus sp. oral taxon 190* | 1 | 0.001 | 0.004 | Intrasporangiaceae |  |
| Bacteria | 141451 | *Oleiphilus messinensis* | 1 | 0.001 | 0.004 | Oleiphilaceae |  |
| Bacteria | 357233 | *Siccibacter turicensis* | 1 | 0.001 | 0.004 | Enterobacteriaceae |  |
| Bacteria | 1917158 | *Alteromonas sp. RW2A1* | 1 | 0.001 | 0.004 | Alteromonadaceae |  |
| Bacteria | 315405 | *Streptococcus gallolyticus* | 1 | 0.001 | 0.004 | Streptococcaceae |  |
| Bacteria | 351091 | *Oscillibacter valericigenes* | 1 | 0.001 | 0.004 | Oscillospiraceae |  |
| Bacteria | 1519 | *Clostridium tyrobutyricum* | 1 | 0.001 | 0.004 | Clostridiaceae |  |
| Bacteria | 28068 | *Rubrivivax gelatinosus* | 1 | 0.001 | 0.004 | NO_NAME |  |
| Bacteria | 225194 | *Geobacter bemidjiensis* | 1 | 0.001 | 0.004 | Geobacteraceae |  |
| Bacteria | 1461583 | *Lysinibacillus saudimassiliensis* | 1 | 0.001 | 0.004 | Bacillaceae |  |
| Bacteria | 1517 | *Thermoanaerobacterium thermosaccharolyticum* | 1 | 0.001 | 0.004 | Thermoanaerobacterales |  |
| Bacteria | 1513 | *Clostridium tetani* | 1 | 0.001 | 0.004 | Clostridiaceae |  |
| Bacteria | 359303 | *Shewanella loihica* | 1 | 0.001 | 0.004 | Shewanellaceae |  |
| Bacteria | 155892 | *Caulobacter vibrioides* | 1 | 0.001 | 0.004 | Caulobacteraceae |  |
| Bacteria | 1219382 | *Acinetobacter nectaris* | 1 | 0.001 | 0.004 | Moraxellaceae |  |
| Bacteria | 1349605 | *Aquabacterium sp. A7-Y* | 1 | 0.001 | 0.004 | NO_NAME |  |
| Bacteria | 28169 | *Vibrio pelagius* | 1 | 0.001 | 0.004 | Vibrionaceae |  |
| Bacteria | 172042 | *Rothia aeria* | 1 | 0.001 | 0.004 | Micrococcaceae |  |
| Bacteria | 484429 | *Sphingobium sp. YBL2* | 1 | 0.001 | 0.004 | Sphingomonadaceae |  |
| Bacteria | 2743 | *Marinobacter hydrocarbonoclasticus* | 1 | 0.001 | 0.004 | Alteromonadaceae |  |
| Bacteria | 1193095 | *Lactobacillus hokkaidonensis* | 1 | 0.001 | 0.004 | Lactobacillaceae |  |
| Bacteria | 225992 | *Comamonas kerstersii* | 1 | 0.001 | 0.004 | Comamonadaceae |  |
| Bacteria | 675864 | *Auraticoccus monumenti* | 1 | 0.001 | 0.004 | Propionibacteriaceae |  |
| Bacteria | 108980 | *Acinetobacter ursingii* | 1 | 0.001 | 0.004 | Moraxellaceae |  |
| Bacteria | 1408 | *Bacillus pumilus* | 1 | 0.001 | 0.004 | Bacillaceae |  |
| Bacteria | 897 | *Desulfococcus multivorans* | 1 | 0.001 | 0.004 | Desulfobacteraceae |  |
| Bacteria | 103817 | *Janibacter terrae* | 1 | 0.001 | 0.004 | Intrasporangiaceae |  |
| Bacteria | 220990 | *Asaia krungthepensis* | 1 | 0.001 | 0.004 | Acetobacteraceae |  |
| Bacteria | 1007105 | *Pusillimonas sp. T7-7* | 1 | 0.001 | 0.004 | Alcaligenaceae |  |
| Bacteria | 83683 | *Lactobacillus amylolyticus* | 1 | 0.001 | 0.004 | Lactobacillaceae |  |
| Bacteria | 309867 | *Acinetobacter sp. LUH5605* | 1 | 0.001 | 0.004 | Moraxellaceae |  |
| Bacteria | 674703 | *Rhodoplanes sp. Z2-YC6860* | 1 | 0.001 | 0.004 | Hyphomicrobiaceae |  |
| Bacteria | 988804 | *Enterobacteriaceae bacterium 8NS6* | 1 | 0.001 | 0.004 | Enterobacteriaceae |  |
| Bacteria | 354 | *Azotobacter vinelandii* | 1 | 0.001 | 0.004 | Pseudomonadaceae |  |
| Bacteria | 54571 | *Streptomyces venezuelae* | 1 | 0.001 | 0.004 | Streptomycetaceae |  |
| Bacteria | 1150389 | *Flavobacteriaceae bacterium UJ101* | 1 | 0.001 | 0.004 | Flavobacteriaceae |  |
| Bacteria | 33934 | *Anoxybacillus flavithermus* | 1 | 0.001 | 0.004 | Bacillaceae |  |
| Bacteria | 1324352 | *Chryseobacterium gallinarum* | 1 | 0.001 | 0.004 | Flavobacteriaceae |  |
| Bacteria | 1579979 | *Wenzhouxiangella marina* | 1 | 0.001 | 0.004 | Wenzhouxiangellaceae |  |
| Bacteria | 55508 | *Janthinobacterium agaricidamnosum* | 1 | 0.001 | 0.004 | Oxalobacteraceae |  |
| Bacteria | 698828 | *Kushneria sp. X49* | 1 | 0.001 | 0.004 | Halomonadaceae |  |
| Bacteria | 2017485 | *Brachybacterium sp. VR2415* | 1 | 0.001 | 0.004 | Dermabacteraceae |  |
| Bacteria | 90270 | *Xanthomonas gardneri* | 1 | 0.001 | 0.004 | Xanthomonadaceae |  |
| Bacteria | 290111 | *Xenorhabdus ehlersii* | 1 | 0.001 | 0.004 | Morganellaceae |  |
| Bacteria | 571913 | *Luteipulveratus mongoliensis* | 1 | 0.001 | 0.004 | Dermacoccaceae |  |
| Bacteria | 92947 | *Ketogulonicigenium robustum* | 1 | 0.001 | 0.004 | Rhodobacteraceae |  |
| Bacteria | 1076 | *Rhodopseudomonas palustris* | 1 | 0.001 | 0.004 | Bradyrhizobiaceae |  |
| Bacteria | 651740 | *Pseudomonas cedrina* | 1 | 0.001 | 0.004 | Pseudomonadaceae |  |
| Bacteria | 253239 | *Ethanoligenens harbinense* | 1 | 0.001 | 0.004 | Ruminococcaceae |  |
| Bacteria | 1306787 | *Alcanivorax pacificus* | 1 | 0.001 | 0.004 | Alcanivoracaceae |  |
| Bacteria | 666 | *Vibrio cholerae* | 1 | 0.001 | 0.004 | Vibrionaceae |  |
| Bacteria | 285 | *Comamonas testosteroni* | 1 | 0.001 | 0.004 | Comamonadaceae |  |
| Bacteria | 1768242 | *Paucibacter sp. KCTC 42545* | 1 | 0.001 | 0.004 | NO_NAME |  |
| Bacteria | 357276 | *Bacteroides dorei* | 1 | 0.001 | 0.004 | Bacteroidaceae |  |
| Bacteria | 1774273 | *Polaribacter vadi* | 1 | 0.001 | 0.004 | Flavobacteriaceae |  |
| Bacteria | 85085 | *Pseudarthrobacter chlorophenolicus* | 1 | 0.001 | 0.004 | Micrococcaceae |  |
| Bacteria | 38289 | *Corynebacterium jeikeium* | 1 | 0.001 | 0.004 | Corynebacteriaceae |  |
| Bacteria | 1810504 | *Immundisolibacter cernigliae* | 1 | 0.001 | 0.004 | Immundisolibacteraceae |  |
| Bacteria | 28025 | *Bifidobacterium animalis* | 1 | 0.001 | 0.004 | Bifidobacteriaceae |  |
| Bacteria | 43669 | *Brachybacterium faecium* | 1 | 0.001 | 0.004 | Dermabacteraceae |  |
| Bacteria | 63 | *Vitreoscilla filiformis* | 1 | 0.001 | 0.004 | Neisseriaceae |  |
| Bacteria | 686597 | *Martelella sp. AD-3* | 1 | 0.001 | 0.004 | Aurantimonadaceae |  |
| Bacteria | 171437 | *Tistrella mobilis* | 1 | 0.001 | 0.004 | Rhodospirillaceae |  |
| Bacteria | 365349 | *Pelosinus fermentans* | 1 | 0.001 | 0.004 | Sporomusaceae |  |
| Bacteria | 232721 | *Acidovorax sp. JS42* | 1 | 0.001 | 0.004 | Comamonadaceae |  |
| Bacteria | 198620 | *Pseudomonas koreensis* | 1 | 0.001 | 0.004 | Pseudomonadaceae |  |
| Bacteria | 463014 | *Bordetella flabilis* | 1 | 0.001 | 0.004 | Alcaligenaceae |  |
| Bacteria | 1111069 | *Thermus sp. CCB_US3_UF1* | 1 | 0.001 | 0.004 | Thermaceae |  |
| Bacteria | 667019 | *Curvibacter putative symbiont of Hydra magnipapillata* | 1 | 0.001 | 0.004 | Comamonadaceae |  |
| Bacteria | 170573 | *Staphylococcus pettenkoferi* | 1 | 0.001 | 0.004 | Staphylococcaceae |  |
| Bacteria | 1416803 | *Bordetella genomosp. 9* | 1 | 0.001 | 0.004 | Alcaligenaceae |  |
| Bacteria | 1566358 | *Paenibacillus sp. IHBB 10380* | 1 | 0.001 | 0.004 | Paenibacillaceae |  |
| Bacteria | 419007 | *Tatumella terrea* | 1 | 0.001 | 0.004 | Erwiniaceae |  |
| Bacteria | 419475 | *Ochrobactrum pseudogrignonense* | 1 | 0.001 | 0.004 | Brucellaceae |  |
| Bacteria | 200450 | *Pseudomonas trivialis* | 1 | 0.001 | 0.004 | Pseudomonadaceae |  |
| Bacteria | 419479 | *Jiangella alkaliphila* | 1 | 0.001 | 0.004 | Jiangellaceae |  |
| Bacteria | 1572656 | *Ruminococcaceae bacterium CPB6* | 1 | 0.001 | 0.004 | Ruminococcaceae |  |
| Bacteria | 38308 | *Gluconobacter frateurii* | 1 | 0.001 | 0.004 | Acetobacteraceae |  |
| Bacteria | 189426 | *Paenibacillus odorifer* | 1 | 0.001 | 0.004 | Paenibacillaceae |  |
| Bacteria | 415421 | *Asaia lannensis* | 1 | 0.001 | 0.004 | Acetobacteraceae |  |
| Bacteria | 80869 | *Acidovorax citrulli* | 1 | 0.001 | 0.004 | Comamonadaceae |  |
| Bacteria | 576610 | *Polynucleobacter necessarius* | 1 | 0.001 | 0.004 | Burkholderiaceae |  |
| Bacteria | 1207075 | *Pseudomonas sp. UW4* | 1 | 0.001 | 0.004 | Pseudomonadaceae |  |
| Bacteria | 132919 | *Rhodococcus jostii* | 1 | 0.001 | 0.004 | Nocardiaceae |  |
| Bacteria | 1736 | *Eubacterium limosum* | 1 | 0.001 | 0.004 | Eubacteriaceae |  |
| Bacteria | 51229 | *Wigglesworthia glossinidia* | 1 | 0.001 | 0.004 | Erwiniaceae |  |
| Bacteria | 71657 | *Lonsdalea quercina* | 1 | 0.001 | 0.004 | Pectobacteriaceae |  |
| Bacteria | 56448 | *Xanthomonas arboricola* | 1 | 0.001 | 0.004 | Xanthomonadaceae |  |
| Bacteria | 1199245 | *secondary endosymbiont of Ctenarytaina eucalypti* | 1 | 0.001 | 0.004 | Enterobacteriaceae |  |
| Bacteria | 84590 | *Taylorella asinigenitalis* | 1 | 0.001 | 0.004 | Alcaligenaceae |  |
| Bacteria | 84595 | *Gordonia polyisoprenivorans* | 1 | 0.001 | 0.004 | Gordoniaceae |  |
| Bacteria | 988718 | *Acinetobacter sp. 18N3* | 1 | 0.001 | 0.004 | Moraxellaceae |  |
| Bacteria | 1909395 | *Nonomuraea sp. ATCC 55076* | 1 | 0.001 | 0.004 | Streptosporangiaceae |  |
| Bacteria | 1596 | *Lactobacillus gasseri* | 1 | 0.001 | 0.004 | Lactobacillaceae |  |
| Bacteria | 168471 | *Laribacter hongkongensis* | 1 | 0.001 | 0.004 | Chromobacteriaceae |  |
| Bacteria | 1348774 | *Croceicoccus naphthovorans* | 1 | 0.001 | 0.004 | Erythrobacteraceae |  |
| Bacteria | 1985254 | *Xanthomonas phaseoli* | 1 | 0.001 | 0.004 | Xanthomonadaceae |  |
| Bacteria | 47877 | *Pseudomonas amygdali* | 1 | 0.001 | 0.004 | Pseudomonadaceae |  |
| Bacteria | 47879 | *Pseudomonas corrugata* | 1 | 0.001 | 0.004 | Pseudomonadaceae |  |
| Bacteria | 47878 | *Pseudomonas azotoformans* | 1 | 0.001 | 0.004 | Pseudomonadaceae |  |
| Bacteria | 1855331 | *Pseudomonas sp. A214* | 1 | 0.001 | 0.004 | Pseudomonadaceae |  |
| Bacteria | 1148509 | *Pseudomonas prosekii* | 1 | 0.001 | 0.004 | Pseudomonadaceae |  |
| Bacteria | 1747 | *Cutibacterium acnes* | 1 | 0.001 | 0.004 | Propionibacteriaceae |  |
| Bacteria | 1004322 | *Vibrio crosai* | 1 | 0.001 | 0.004 | Vibrionaceae |  |
| Bacteria | 169679 | *Clostridium saccharobutylicum* | 1 | 0.001 | 0.004 | Clostridiaceae |  |
| Bacteria | 1030157 | *Sphingomonas sp. KC8* | 1 | 0.001 | 0.004 | Sphingomonadaceae |  |
| Bacteria | 34073 | *Variovorax paradoxus* | 1 | 0.001 | 0.004 | Comamonadaceae |  |
| Bacteria | 89059 | *Lactobacillus acidipiscis* | 1 | 0.001 | 0.004 | Lactobacillaceae |  |
| Bacteria | 1720344 | *Psychrobacter sp. AntiMn-1* | 1 | 0.001 | 0.004 | Moraxellaceae |  |
| Bacteria | 1644131 | *Janthinobacterium sp. 1_2014MBL_MicDiv* | 1 | 0.001 | 0.004 | Oxalobacteraceae |  |
| Bacteria | 94254 | *Dyadobacter fermentans* | 1 | 0.001 | 0.004 | Cytophagaceae |  |
| Bacteria | 2134 | *Spiroplasma melliferum* | 1 | 0.001 | 0.004 | Spiroplasmataceae |  |
| Bacteria | 1488 | *Clostridium acetobutylicum* | 1 | 0.001 | 0.004 | Clostridiaceae |  |
| Bacteria | 1323749 | *Vibrio salilacus* | 1 | 0.001 | 0.004 | Vibrionaceae |  |
| Bacteria | 267818 | *Lactobacillus kefiranofaciens* | 1 | 0.001 | 0.004 | Lactobacillaceae |  |
| Bacteria | 138336 | *Blastococcus saxobsidens* | 1 | 0.001 | 0.004 | Geodermatophilaceae |  |
| Bacteria | 104100 | *Acetobacter lovaniensis* | 1 | 0.001 | 0.004 | Acetobacteraceae |  |
| Bacteria | 46679 | *Pseudomonas mucidolens* | 1 | 0.001 | 0.004 | Pseudomonadaceae |  |
| Bacteria | 28077 | *Nitrospirillum amazonense* | 1 | 0.001 | 0.004 | Rhodospirillaceae |  |
| Bacteria | 1296 | *Staphylococcus sciuri* | 1 | 0.001 | 0.004 | Staphylococcaceae |  |
| Bacteria | 43657 | *Pseudoalteromonas luteoviolacea* | 1 | 0.001 | 0.004 | Pseudoalteromonadaceae |  |
| Bacteria | 862751 | *Streptomyces sp. SirexAA-E* | 1 | 0.001 | 0.004 | Streptomycetaceae |  |
| Bacteria | 51663 | *Pediococcus damnosus* | 1 | 0.001 | 0.004 | Lactobacillaceae |  |
| Bacteria | 1357916 | *Sphingopyxis sp. QXT-31* | 1 | 0.001 | 0.004 | Sphingomonadaceae |  |
| Bacteria | 988795 | *Enterobacteriaceae bacterium 4NS5* | 1 | 0.001 | 0.004 | Enterobacteriaceae |  |
| Bacteria | 1332080 | *Sphingobium baderi* | 1 | 0.001 | 0.004 | Sphingomonadaceae |  |
| Bacteria | 1560345 | *Sphingomonas panacis* | 1 | 0.001 | 0.004 | Sphingomonadaceae |  |
| Bacteria | 480 | *Moraxella catarrhalis* | 1 | 0.001 | 0.004 | Moraxellaceae |  |
| Bacteria | 133924 | *Leisingera methylohalidivorans* | 1 | 0.001 | 0.004 | Rhodobacteraceae |  |
| Bacteria | 160799 | *Paenibacillus borealis* | 1 | 0.001 | 0.004 | Paenibacillaceae |  |
| Bacteria | 879274 | *Shinella sp. HZN7* | 1 | 0.001 | 0.004 | Rhizobiaceae |  |
| Bacteria | 2001 | *Streptosporangium roseum* | 1 | 0.001 | 0.004 | Streptosporangiaceae |  |
| Bacteria | 988707 | *Acinetobacter sp. 12N1* | 1 | 0.001 | 0.004 | Moraxellaceae |  |
| Bacteria | 589382 | *Agromyces flavus* | 1 | 0.001 | 0.004 | Microbacteriaceae |  |
| Bacteria | 238013 | *Lactobacillus rennini* | 1 | 0.001 | 0.004 | Lactobacillaceae |  |
| Bacteria | 152297 | *Pseudoalteromonas issachenkonii* | 1 | 0.001 | 0.004 | Pseudoalteromonadaceae |  |
| Viruses | 1100043 | *Apis mellifera filamentous virus* | 172213 | 99.969 | 650.098 | NO_NAME |  |
| Viruses | 1285595 | *Musca hytrovirus* | 22 | 0.013 | 0.083 | Hytrosaviridae |  |
| Viruses | 632112 | *Lactobacillus virus Lb338-1* | 9 | 0.005 | 0.034 | Myoviridae |  |
| Viruses | 925984 | *Erwinia phage phiEt88* | 2 | 0.001 | 0.008 | Myoviridae |  |
| Viruses | 1759525 | *Bacillus phage vB_BceS-MY192* | 2 | 0.001 | 0.008 | Siphoviridae |  |
| Viruses | 1985291 | *Escherichia virus SUSP2* | 2 | 0.001 | 0.008 | Myoviridae |  |
| Viruses | 354260 | *Lactococcus phage P335 sensu lato* | 1 | 0.001 | 0.004 | Siphoviridae |  |
| Viruses | 1985354 | *Escherichia virus RB49* | 1 | 0.001 | 0.004 | Myoviridae |  |
| Viruses | 340054 | *Bacteriophage APSE-2* | 1 | 0.001 | 0.004 | Podoviridae |  |
| Viruses | 1249500 | *Bacteriophage APSE* | 1 | 0.001 | 0.004 | Podoviridae |  |
| Viruses | 2011074 | *Spiroplasma virus SVTS2* | 1 | 0.001 | 0.004 | Inoviridae |  |
| Viruses | 1608311 | *Edwardsiella phage PEi26* | 1 | 0.001 | 0.004 | Myoviridae |  |
| Viruses | 1985341 | *Citrobacter virus Merlin* | 1 | 0.001 | 0.004 | Myoviridae |  |
| Viruses | 1965363 | *Yersinia phage fHe-Yen9-01* | 1 | 0.001 | 0.004 | Myoviridae |  |

**Table F.** List of species identified in the orange tree blossom honey and number of reads assigned considering the ≥97% identity level (see Table C for explanation of % of reads).

| **Groups** | **Taxid** | **Scientific name - species** | **No. of reads** | **% of reads** | **‰ of reads_TOT_** | **Family** | **Order** |
| --- | --- | --- | --- | --- | --- | --- | --- |
| Arthropods | 7460 | *Apis mellifera* | 37 | 35.238 | 0.137 | Apidae | Hymenoptera |
| Arthropods | 28612 | *Rhagoletis zephyria* | 7 | 6.667 | 0.026 | Tephritidae | Diptera |
| Arthropods | 7260 | *Drosophila willistoni* | 3 | 2.857 | 0.011 | Drosophilidae | Diptera |
| Arthropods | 597456 | *Habropoda laboriosa* | 3 | 2.857 | 0.011 | Apidae | Hymenoptera |
| Arthropods | 104508 | *Euphydryas editha* | 1 | 0.952 | 0.004 | Nymphalidae | Lepidoptera |
| Arthropods | 41112 | *Oryzaephilus surinamensis* | 1 | 0.952 | 0.004 | Silvanidae | Coleoptera |
| Arthropods | 7029 | *Acyrthosiphon pisum* | 1 | 0.952 | 0.004 | Aphididae | Hemiptera |
| Plants | 2711 | *Citrus sinensis* | 37 | 19.892 | 0.137 | Rutaceae |  |
| Plants | 85681 | *Citrus clementina* | 32 | 17.204 | 0.118 | Rutaceae |  |
| Plants | 29760 | *Vitis vinifera* | 30 | 16.129 | 0.111 | Vitaceae |  |
| Plants | 4565 | *Triticum aestivum* | 3 | 1.613 | 0.011 | Poaceae |  |
| Plants | 299721 | *Sulla coronaria* | 3 | 1.613 | 0.011 | Fabaceae |  |
| Plants | 451732 | *Taverniera cuneifolia* | 2 | 1.075 | 0.007 | Fabaceae |  |
| Plants | 3899 | *Trifolium repens* | 2 | 1.075 | 0.007 | Fabaceae |  |
| Plants | 34305 | *Lotus japonicus* | 2 | 1.075 | 0.007 | Fabaceae |  |
| Plants | 3906 | *Vicia faba* | 1 | 0.538 | 0.004 | Fabaceae |  |
| Plants | 3711 | *Brassica rapa* | 1 | 0.538 | 0.004 | Brassicaceae |  |
| Plants | 3821 | *Cajanus cajan* | 1 | 0.538 | 0.004 | Fabaceae |  |
| Plants | 85571 | *Citrus reticulata* | 1 | 0.538 | 0.004 | Rutaceae |  |
| Plants | 3505 | *Betula pendula* | 1 | 0.538 | 0.004 | Betulaceae |  |
| Plants | 38942 | *Quercus robur* | 1 | 0.538 | 0.004 | Fagaceae |  |
| Plants | 39329 | *Lavandula angustifolia* | 1 | 0.538 | 0.004 | Lamiaceae |  |
| Plants | 13894 | *Cocos nucifera* | 1 | 0.538 | 0.004 | Arecaceae |  |
| Plants | 185702 | *Apios americana* | 1 | 0.538 | 0.004 | Fabaceae |  |
| Plants | 109171 | *Pachyrhizus erosus* | 1 | 0.538 | 0.004 | Fabaceae |  |
| Plants | 3694 | *Populus trichocarpa* | 1 | 0.538 | 0.004 | Salicaceae |  |
| Plants | 3854 | *Lathyrus aphaca* | 1 | 0.538 | 0.004 | Fabaceae |  |
| Plants | 159030 | *Murraya koenigii* | 1 | 0.538 | 0.004 | Rutaceae |  |
| Plants | 872936 | *Onobrychis caput-galli* | 1 | 0.538 | 0.004 | Fabaceae |  |
| Plants | 4498 | *Avena sativa* | 1 | 0.538 | 0.004 | Poaceae |  |
| Plants | 2708 | *Citrus limon* | 1 | 0.538 | 0.004 | Rutaceae |  |
| Plants | 245239 | *Oxalis latifolia* | 1 | 0.538 | 0.004 | Oxalidaceae |  |
| Plants | 3914 | *Vigna angularis* | 1 | 0.538 | 0.004 | Fabaceae |  |
| Plants | 229543 | *Hibiscus cannabinus* | 1 | 0.538 | 0.004 | Malvaceae |  |
| Plants | 51503 | *Cynomorium coccineum* | 1 | 0.538 | 0.004 | Cynomoriaceae |  |
| Plants | 38783 | *Avena strigosa* | 1 | 0.538 | 0.004 | Poaceae |  |
| Plants | 4522 | *Lolium perenne* | 1 | 0.538 | 0.004 | Poaceae |  |
| Plants | 13451 | *Corylus avellana* | 1 | 0.538 | 0.004 | Betulaceae |  |
| Plants | 981085 | *Morus notabilis* | 1 | 0.538 | 0.004 | Moraceae |  |
| Plants | 3988 | *Ricinus communis* | 1 | 0.538 | 0.004 | Euphorbiaceae |  |
| Plants | 3847 | *Glycine max* | 1 | 0.538 | 0.004 | Fabaceae |  |
| Plants | 37334 | *Citrus maxima* | 1 | 0.538 | 0.004 | Rutaceae |  |
| Plants | 20805 | *Oxytropis lambertii* | 1 | 0.538 | 0.004 | Fabaceae |  |
| Plants | 28974 | *Averrhoa carambola* | 1 | 0.538 | 0.004 | Oxalidaceae |  |
| Plants | 204362 | *Pinguicula vulgaris* | 1 | 0.538 | 0.004 | Lentibulariaceae |  |
| Fungi | 1108849 | *Penicillium rubens* | 18 | 9.677 | 0.067 | Aspergillaceae |  |
| Fungi | 500148 | *Metarhizium brunneum* | 10 | 5.376 | 0.037 | Clavicipitaceae |  |
| Fungi | 42258 | *Zygosaccharomyces mellis* | 5 | 2.688 | 0.018 | Saccharomycetaceae |  |
| Fungi | 1365886 | *Zygosaccharomyces parabailii* | 3 | 1.613 | 0.011 | Saccharomycetaceae |  |
| Fungi | 5599 | *Alternaria alternata* | 3 | 1.613 | 0.011 | Pleosporaceae |  |
| Fungi | 4956 | *Zygosaccharomyces rouxii* | 2 | 1.075 | 0.007 | Saccharomycetaceae |  |
| Fungi | 40559 | *Botrytis cinerea* | 2 | 1.075 | 0.007 | Sclerotiniaceae |  |
| Fungi | 162425 | *Aspergillus nidulans* | 1 | 0.538 | 0.004 | Aspergillaceae |  |
| Fungi | 79858 | *Pseudogymnoascus pannorum* | 1 | 0.538 | 0.004 | Pseudeurotiaceae |  |
| Fungi | 1071840 | *Bettsia alvei* | 1 | 0.538 | 0.004 | NO_NAME |  |
| Fungi | 112493 | *Teratosphaeria molleriana* | 1 | 0.538 | 0.004 | Teratosphaeriaceae |  |
| Fungi | 159246 | *Monascus eremophilus* | 1 | 0.538 | 0.004 | Aspergillaceae |  |
| Fungi | 307758 | *Ustilago bromivora* | 1 | 0.538 | 0.004 | Ustilaginaceae |  |
| Bacteria | 148814 | *Lactobacillus kunkeei* | 996 | 4.963 | 3.683 | Lactobacillaceae |  |
| Bacteria | 549 | *Pantoea agglomerans* | 419 | 2.088 | 1.549 | Erwiniaceae |  |
| Bacteria | 317 | *Pseudomonas syringae* | 317 | 1.580 | 1.172 | Pseudomonadaceae |  |
| Bacteria | 2033 | *Microbacterium testaceum* | 207 | 1.031 | 0.765 | Microbacteriaceae |  |
| Bacteria | 1906742 | *Microbacterium sp. BH-3-3-3* | 97 | 0.483 | 0.359 | Microbacteriaceae |  |
| Bacteria | 1510841 | *Parasaccharibacter apium* | 54 | 0.269 | 0.200 | Acetobacteraceae |  |
| Bacteria | 2133 | *Spiroplasma citri* | 46 | 0.229 | 0.170 | Spiroplasmataceae |  |
| Bacteria | 33069 | *Pseudomonas viridiflava* | 23 | 0.115 | 0.085 | Pseudomonadaceae |  |
| Bacteria | 2137 | *Spiroplasma apis* | 21 | 0.105 | 0.078 | Spiroplasmataceae |  |
| Bacteria | 329 | *Ralstonia pickettii* | 21 | 0.105 | 0.078 | Burkholderiaceae |  |
| Bacteria | 376 | *Bradyrhizobium sp.* | 17 | 0.085 | 0.063 | Bradyrhizobiaceae |  |
| Bacteria | 1355477 | *Bradyrhizobium diazoefficiens* | 17 | 0.085 | 0.063 | Bradyrhizobiaceae |  |
| Bacteria | 1223566 | *Bradyrhizobium sp. CCGE-LA001* | 15 | 0.075 | 0.055 | Bradyrhizobiaceae |  |
| Bacteria | 335659 | *Bradyrhizobium sp. S23321* | 13 | 0.065 | 0.048 | Bradyrhizobiaceae |  |
| Bacteria | 375 | *Bradyrhizobium japonicum* | 12 | 0.060 | 0.044 | Bradyrhizobiaceae |  |
| Bacteria | 638 | *Arsenophonus nasoniae* | 11 | 0.055 | 0.041 | Morganellaceae |  |
| Bacteria | 1267021 | *Frischella perrara* | 10 | 0.050 | 0.037 | Orbaceae |  |
| Bacteria | 1545702 | *Lactobacillus sp. wkB8* | 10 | 0.050 | 0.037 | Lactobacillaceae |  |
| Bacteria | 1619313 | *Erwinia gerundensis* | 8 | 0.040 | 0.030 | Erwiniaceae |  |
| Bacteria | 1686310 | *Bartonella apis* | 7 | 0.035 | 0.026 | Bartonellaceae |  |
| Bacteria | 294 | *Pseudomonas fluorescens* | 6 | 0.030 | 0.022 | Pseudomonadaceae |  |
| Bacteria | 84292 | *Microbacterium chocolatum* | 6 | 0.030 | 0.022 | Microbacteriaceae |  |
| Bacteria | 29438 | *Pseudomonas savastanoi* | 6 | 0.030 | 0.022 | Pseudomonadaceae |  |
| Bacteria | 1747 | *Cutibacterium acnes* | 6 | 0.030 | 0.022 | Propionibacteriaceae |  |
| Bacteria | 33934 | *Anoxybacillus flavithermus* | 5 | 0.025 | 0.018 | Bacillaceae |  |
| Bacteria | 1196095 | *Gilliamella apicola* | 5 | 0.025 | 0.018 | Orbaceae |  |
| Bacteria | 182337 | *Erwinia billingiae* | 5 | 0.025 | 0.018 | Erwiniaceae |  |
| Bacteria | 1274631 | *Bradyrhizobium icense* | 4 | 0.020 | 0.015 | Bradyrhizobiaceae |  |
| Bacteria | 1076 | *Rhodopseudomonas palustris* | 4 | 0.020 | 0.015 | Bradyrhizobiaceae |  |
| Bacteria | 1437360 | *Bradyrhizobium erythrophlei* | 4 | 0.020 | 0.015 | Bradyrhizobiaceae |  |
| Bacteria | 1882747 | *Afipia sp. GAS231* | 4 | 0.020 | 0.015 | Bradyrhizobiaceae |  |
| Bacteria | 76758 | *Pseudomonas orientalis* | 4 | 0.020 | 0.015 | Pseudomonadaceae |  |
| Bacteria | 232721 | *Acidovorax sp. JS42* | 4 | 0.020 | 0.015 | Comamonadaceae |  |
| Bacteria | 1196083 | *Snodgrassella alvi* | 4 | 0.020 | 0.015 | Neisseriaceae |  |
| Bacteria | 571 | *Klebsiella oxytoca* | 3 | 0.015 | 0.011 | Enterobacteriaceae |  |
| Bacteria | 1604 | *Lactobacillus amylovorus* | 3 | 0.015 | 0.011 | Lactobacillaceae |  |
| Bacteria | 912630 | *Microbacterium sp. LKL04* | 3 | 0.015 | 0.011 | Microbacteriaceae |  |
| Bacteria | 79967 | *Erwinia pyrifoliae* | 3 | 0.015 | 0.011 | Erwiniaceae |  |
| Bacteria | 288000 | *Bradyrhizobium sp. BTAi1* | 3 | 0.015 | 0.011 | Bradyrhizobiaceae |  |
| Bacteria | 215689 | *Erwinia sp. Ejp617* | 3 | 0.015 | 0.011 | Erwiniaceae |  |
| Bacteria | 40324 | *Stenotrophomonas maltophilia* | 3 | 0.015 | 0.011 | Xanthomonadaceae |  |
| Bacteria | 263478 | *rice phyllosphere bacterium A* | 3 | 0.015 | 0.011 | NO_NAME |  |
| Bacteria | 2751 | *Carnobacterium maltaromaticum* | 3 | 0.015 | 0.011 | Carnobacteriaceae |  |
| Bacteria | 470934 | *Pantoea vagans* | 3 | 0.015 | 0.011 | Erwiniaceae |  |
| Bacteria | 300019 | *Microbacterium paludicola* | 2 | 0.010 | 0.007 | Microbacteriaceae |  |
| Bacteria | 29466 | *Veillonella parvula* | 2 | 0.010 | 0.007 | Veillonellaceae |  |
| Bacteria | 287 | *Pseudomonas aeruginosa* | 2 | 0.010 | 0.007 | Pseudomonadaceae |  |
| Bacteria | 47834 | *Spiroplasma kunkelii* | 2 | 0.010 | 0.007 | Spiroplasmataceae |  |
| Bacteria | 40214 | *Acinetobacter johnsonii* | 2 | 0.010 | 0.007 | Moraxellaceae |  |
| Bacteria | 1546149 | *Diaphorobacter polyhydroxybutyrativorans* | 2 | 0.010 | 0.007 | Comamonadaceae |  |
| Bacteria | 256701 | *Glutamicibacter arilaitensis* | 2 | 0.010 | 0.007 | Micrococcaceae |  |
| Bacteria | 339 | *Xanthomonas campestris* | 2 | 0.010 | 0.007 | Xanthomonadaceae |  |
| Bacteria | 573 | *Klebsiella pneumoniae* | 2 | 0.010 | 0.007 | Enterobacteriaceae |  |
| Bacteria | 562 | *Escherichia coli* | 2 | 0.010 | 0.007 | Enterobacteriaceae |  |
| Bacteria | 1590 | *Lactobacillus plantarum* | 2 | 0.010 | 0.007 | Lactobacillaceae |  |
| Bacteria | 1583341 | *Pseudomonas cerasi* | 2 | 0.010 | 0.007 | Pseudomonadaceae |  |
| Bacteria | 552 | *Erwinia amylovora* | 2 | 0.010 | 0.007 | Erwiniaceae |  |
| Bacteria | 1358 | *Lactococcus lactis* | 2 | 0.010 | 0.007 | Streptococcaceae |  |
| Bacteria | 338565 | *Erwinia tasmaniensis* | 2 | 0.010 | 0.007 | Erwiniaceae |  |
| Bacteria | 553 | *Pantoea ananatis* | 2 | 0.010 | 0.007 | Erwiniaceae |  |
| Bacteria | 200451 | *Pseudomonas poae* | 2 | 0.010 | 0.007 | Pseudomonadaceae |  |
| Bacteria | 1282 | *Staphylococcus epidermidis* | 2 | 0.010 | 0.007 | Staphylococcaceae |  |
| Bacteria | 722472 | *Bradyrhizobium lablabi* | 2 | 0.010 | 0.007 | Bradyrhizobiaceae |  |
| Bacteria | 291112 | *Photorhabdus asymbiotica* | 1 | 0.005 | 0.004 | Morganellaceae |  |
| Bacteria | 546 | *Citrobacter freundii* | 1 | 0.005 | 0.004 | Enterobacteriaceae |  |
| Bacteria | 2151 | *Mesoplasma florum* | 1 | 0.005 | 0.004 | Entomoplasmataceae |  |
| Bacteria | 1464 | *Paenibacillus larvae* | 1 | 0.005 | 0.004 | Paenibacillaceae |  |
| Bacteria | 180282 | *Delftia tsuruhatensis* | 1 | 0.005 | 0.004 | Comamonadaceae |  |
| Bacteria | 115808 | *Bradyrhizobium sp. ORS 285* | 1 | 0.005 | 0.004 | Bradyrhizobiaceae |  |
| Bacteria | 82996 | *Serratia plymuthica* | 1 | 0.005 | 0.004 | Yersiniaceae |  |
| Bacteria | 1891675 | *Pantoea alhagi* | 1 | 0.005 | 0.004 | Erwiniaceae |  |
| Bacteria | 158822 | *Cedecea neteri* | 1 | 0.005 | 0.004 | Enterobacteriaceae |  |
| Bacteria | 43771 | *Corynebacterium urealyticum* | 1 | 0.005 | 0.004 | Corynebacteriaceae |  |
| Bacteria | 69373 | *Curtobacterium pusillum* | 1 | 0.005 | 0.004 | Microbacteriaceae |  |
| Bacteria | 69370 | *Microbacterium trichothecenolyticum* | 1 | 0.005 | 0.004 | Microbacteriaceae |  |
| Bacteria | 48935 | *Novosphingobium aromaticivorans* | 1 | 0.005 | 0.004 | Sphingomonadaceae |  |
| Bacteria | 247637 | *marine gamma proteobacterium HTCC2246* | 1 | 0.005 | 0.004 | Halieaceae |  |
| Bacteria | 28037 | *Streptococcus mitis* | 1 | 0.005 | 0.004 | Streptococcaceae |  |
| Bacteria | 1905847 | *Curtobacterium sp. BH-2-1-1* | 1 | 0.005 | 0.004 | Microbacteriaceae |  |
| Bacteria | 1921510 | *Sphingomonas sp. JJ-A5* | 1 | 0.005 | 0.004 | Sphingomonadaceae |  |
| Bacteria | 1404 | *Bacillus megaterium* | 1 | 0.005 | 0.004 | Bacillaceae |  |
| Bacteria | 305 | *Ralstonia solanacearum* | 1 | 0.005 | 0.004 | Burkholderiaceae |  |
| Bacteria | 160386 | *Corynebacterium casei* | 1 | 0.005 | 0.004 | Corynebacteriaceae |  |
| Bacteria | 235568 | *Arsenophonus endosymbiont of Australiococcus greville* | 1 | 0.005 | 0.004 | Morganellaceae |  |
| Bacteria | 40137 | *Oligotropha carboxidovorans* | 1 | 0.005 | 0.004 | Bradyrhizobiaceae |  |
| Bacteria | 29430 | *Acinetobacter haemolyticus* | 1 | 0.005 | 0.004 | Moraxellaceae |  |
| Bacteria | 370764 | *Microbacterium pygmaeum* | 1 | 0.005 | 0.004 | Microbacteriaceae |  |
| Bacteria | 624 | *Shigella sonnei* | 1 | 0.005 | 0.004 | Enterobacteriaceae |  |
| Bacteria | 1230998 | *Corynebacterium frankenforstense* | 1 | 0.005 | 0.004 | Corynebacteriaceae |  |
| Bacteria | 47770 | *Lactobacillus crispatus* | 1 | 0.005 | 0.004 | Lactobacillaceae |  |
| Bacteria | 408 | *Methylobacterium extorquens* | 1 | 0.005 | 0.004 | Methylobacteriaceae |  |
| Bacteria | 1502 | *Clostridium perfringens* | 1 | 0.005 | 0.004 | Clostridiaceae |  |
| Bacteria | 37329 | *Nocardia farcinica* | 1 | 0.005 | 0.004 | Nocardiaceae |  |
| Bacteria | 1479019 | *Methylobacterium sp. C1* | 1 | 0.005 | 0.004 | Methylobacteriaceae |  |
| Bacteria | 633 | *Yersinia pseudotuberculosis* | 1 | 0.005 | 0.004 | Yersiniaceae |  |
| Bacteria | 651 | *Aeromonas media* | 1 | 0.005 | 0.004 | Aeromonadaceae |  |
| Bacteria | 251701 | *Pseudomonas syringae group genomosp. 3* | 1 | 0.005 | 0.004 | Pseudomonadaceae |  |
| Bacteria | 47878 | *Pseudomonas azotoformans* | 1 | 0.005 | 0.004 | Pseudomonadaceae |  |
| Bacteria | 727 | *Haemophilus influenzae* | 1 | 0.005 | 0.004 | Pasteurellaceae |  |
| Bacteria | 186189 | *Xylanimonas cellulosilytica* | 1 | 0.005 | 0.004 | Promicromonosporaceae | |
| Bacteria | 152682 | *Sphingomonas melonis* | 1 | 0.005 | 0.004 | Sphingomonadaceae |  |
| Bacteria | 1643450 | *Devosia sp. H5989* | 1 | 0.005 | 0.004 | Hyphomicrobiaceae |  |
| Bacteria | 729 | *Haemophilus parainfluenzae* | 1 | 0.005 | 0.004 | Pasteurellaceae |  |
| Bacteria | 1076550 | *Pantoea rwandensis* | 1 | 0.005 | 0.004 | Erwiniaceae |  |
| Bacteria | 443602 | *Bradyrhizobium sp. Lop10.4* | 1 | 0.005 | 0.004 | Bradyrhizobiaceae |  |
| Bacteria | 246432 | *Staphylococcus equorum* | 1 | 0.005 | 0.004 | Staphylococcaceae |  |
| Bacteria | 550 | *Enterobacter cloacae* | 1 | 0.005 | 0.004 | Enterobacteriaceae |  |
| Bacteria | 1218496 | *Lactobacillus sp. Fhon2N* | 1 | 0.005 | 0.004 | Lactobacillaceae |  |
| Bacteria | 2134 | *Spiroplasma melliferum* | 1 | 0.005 | 0.004 | Spiroplasmataceae |  |
| Bacteria | 44255 | *Bradyrhizobium oligotrophicum* | 1 | 0.005 | 0.004 | Bradyrhizobiaceae |  |
| Bacteria | 225991 | *Comamonas aquatica* | 1 | 0.005 | 0.004 | Comamonadaceae |  |
| Bacteria | 29486 | *Yersinia ruckeri* | 1 | 0.005 | 0.004 | Yersiniaceae |  |
| Bacteria | 1983105 | *Lusitaniella coriacea* | 1 | 0.005 | 0.004 | NO_NAME |  |
| Bacteria | 438 | *Acetobacter pasteurianus* | 1 | 0.005 | 0.004 | Acetobacteraceae |  |
| Bacteria | 334852 | *Methylobacterium oryzae* | 1 | 0.005 | 0.004 | Methylobacteriaceae |  |
| Bacteria | 55211 | *Erwinia persicina* | 1 | 0.005 | 0.004 | Erwiniaceae |  |
| Bacteria | 161896 | *Corynebacterium camporealensis* | 1 | 0.005 | 0.004 | Corynebacteriaceae |  |
| Bacteria | 1849491 | *Auricoccus indicus* | 1 | 0.005 | 0.004 | Staphylococcaceae |  |
| Bacteria | 1070035 | *Bradyrhizobium sp. ARR560* | 1 | 0.005 | 0.004 | Bradyrhizobiaceae |  |
| Bacteria | 358 | *Agrobacterium tumefaciens* | 1 | 0.005 | 0.004 | Rhizobiaceae |  |
| Bacteria | 1869170 | *Rhizobium sp. S41* | 1 | 0.005 | 0.004 | Rhizobiaceae |  |
| Virus | 1100043 | *Apis mellifera filamentous virus* | 188358 | 75.908 | 696.490 | NO_NAME |  |
| Virus | 2011074 | *Spiroplasma virus SVTS2* | 1 | 0.000 | 0.004 | Inoviridae |  |
| Virus | 113366 | *Heliothis virescens ascovirus 3a* | 1 | 0.000 | 0.004 | Ascoviridae |  |

**Table G.** List of species identified in the eucalyptus tree blossom honey and number of reads assigned considering the ≥97% identity level (see Table C for explanation of % of reads).

| **Groups** | **Taxid** | **Scientific name - species** | **No. of reads** | **% of reads** | **‰ of reads_TOT_** | **Family** | **Order** |
| --- | --- | --- | --- | --- | --- | --- | --- |
| Arthropods | 7460 | *Apis mellifera* | 1458 | 53.270 | 5.504 | Apidae | Hymenoptera |
| Arthropods | 597456 | *Habropoda laboriosa* | 53 | 1.936 | 0.200 | Apidae | Hymenoptera |
| Arthropods | 7461 | *Apis cerana* | 40 | 1.461 | 0.151 | Apidae | Hymenoptera |
| Arthropods | 7462 | *Apis dorsata* | 21 | 0.767 | 0.079 | Apidae | Hymenoptera |
| Arthropods | 7463 | *Apis florea* | 12 | 0.438 | 0.045 | Apidae | Hymenoptera |
| Arthropods | 30025 | *Drosophila ficusphila* | 9 | 0.329 | 0.034 | Drosophilidae | Diptera |
| Arthropods | 29029 | *Drosophila eugracilis* | 8 | 0.292 | 0.030 | Drosophilidae | Diptera |
| Arthropods | 7227 | *Drosophila melanogaster* | 4 | 0.146 | 0.015 | Drosophilidae | Diptera |
| Arthropods | 30023 | *Drosophila elegans* | 4 | 0.146 | 0.015 | Drosophilidae | Diptera |
| Arthropods | 7224 | *Drosophila hydei* | 1 | 0.037 | 0.004 | Drosophilidae | Diptera |
| Arthropods | 7137 | *Galleria mellonella* | 1 | 0.037 | 0.004 | Pyralidae | Lepidoptera |
| Arthropods | 65145 | *Enicospilus ramidulus* | 1 | 0.037 | 0.004 | Ichneumonidae | Hymenoptera |
| Arthropods | 36166 | *Megaselia scalaris* | 1 | 0.037 | 0.004 | Phoridae | Diptera |
| Arthropods | 29030 | *Drosophila takahashii* | 1 | 0.037 | 0.004 | Drosophilidae | Diptera |
| Arthropods | 30019 | *Drosophila busckii* | 1 | 0.037 | 0.004 | Drosophilidae | Diptera |
| Arthropods | 30213 | *Vespula pensylvanica* | 1 | 0.037 | 0.004 | Vespidae | Hymenoptera |
| Arthropods | 58824 | *Plodia interpunctella* | 1 | 0.037 | 0.004 | Pyralidae | Lepidoptera |
| Arthropods | 109461 | *Varroa destructor* | 1 | 0.037 | 0.004 | Varroidae | Parasitiformes |
| Arthropods | 42026 | *Drosophila bipectinata* | 1 | 0.037 | 0.004 | Drosophilidae | Diptera |
| Arthropods | 1536863 | *Aculus sp. NJAUEriHN225* | 1 | 0.037 | 0.004 | Eriophyidae | Trombidiformes |
| Plants | 71139 | *Eucalyptus grandis* | 71 | 10.823 | 0.268 | Myrtaceae |  |
| Plants | 223102 | *Orobanche crenata* | 19 | 2.896 | 0.072 | Orobanchaceae |  |
| Plants | 223129 | *Orobanche rapum-genistae* | 17 | 2.591 | 0.064 | Orobanchaceae |  |
| Plants | 4530 | *Oryza sativa* | 9 | 1.372 | 0.034 | Poaceae |  |
| Plants | 4686 | *Asparagus officinalis* | 8 | 1.220 | 0.030 | Asparagaceae |  |
| Plants | 42345 | *Phoenix dactylifera* | 4 | 0.610 | 0.015 | Arecaceae |  |
| Plants | 3694 | *Populus trichocarpa* | 4 | 0.610 | 0.015 | Salicaceae |  |
| Plants | 119949 | *Myrtus communis* | 3 | 0.457 | 0.011 | Myrtaceae |  |
| Plants | 29760 | *Vitis vinifera* | 2 | 0.305 | 0.008 | Vitaceae |  |
| Plants | 92921 | *Silybum marianum* | 2 | 0.305 | 0.008 | Asteraceae |  |
| Plants | 3906 | *Vicia faba* | 1 | 0.152 | 0.004 | Fabaceae |  |
| Plants | 289753 | *Rhus chinensis* | 1 | 0.152 | 0.004 | Anacardiaceae |  |
| Plants | 3750 | *Malus domestica* | 1 | 0.152 | 0.004 | Rosaceae |  |
| Plants | 183840 | *Eucalyptus microtheca* | 1 | 0.152 | 0.004 | Myrtaceae |  |
| Plants | 3654 | *Citrullus lanatus* | 1 | 0.152 | 0.004 | Cucurbitaceae |  |
| Plants | 183858 | *Eucalyptus torquata* | 1 | 0.152 | 0.004 | Myrtaceae |  |
| Plants | 75702 | *Populus euphratica* | 1 | 0.152 | 0.004 | Salicaceae |  |
| Plants | 3656 | *Cucumis melo* | 1 | 0.152 | 0.004 | Cucurbitaceae |  |
| Plants | 87685 | *Eucalyptus spathulata* | 1 | 0.152 | 0.004 | Myrtaceae |  |
| Plants | 13329 | *Achillea millefolium* | 1 | 0.152 | 0.004 | Asteraceae |  |
| Plants | 4679 | *Allium cepa* | 1 | 0.152 | 0.004 | Amaryllidaceae |  |
| Plants | 3899 | *Trifolium repens* | 1 | 0.152 | 0.004 | Fabaceae |  |
| Plants | 79164 | *Daucus aureus* | 1 | 0.152 | 0.004 | Apiaceae |  |
| Plants | 28964 | *Geranium maderense* | 1 | 0.152 | 0.004 | Geraniaceae |  |
| Plants | 34320 | *Eucalyptus cloeziana* | 1 | 0.152 | 0.004 | Myrtaceae |  |
| Plants | 4039 | *Daucus carota* | 1 | 0.152 | 0.004 | Apiaceae |  |
| Plants | 39352 | *Origanum vulgare* | 1 | 0.152 | 0.004 | Lamiaceae |  |
| Plants | 1711294 | *Eucalyptus extensa* | 1 | 0.152 | 0.004 | Myrtaceae |  |
| Plants | 326968 | *Ziziphus jujuba* | 1 | 0.152 | 0.004 | Rhamnaceae |  |
| Plants | 39511 | *Agave attenuata* | 1 | 0.152 | 0.004 | Asparagaceae |  |
| Plants | 3880 | *Medicago truncatula* | 1 | 0.152 | 0.004 | Fabaceae |  |
| Plants | 3711 | *Brassica rapa* | 1 | 0.152 | 0.004 | Brassicaceae |  |
| Plants | 4155 | *Erythranthe guttata* | 1 | 0.152 | 0.004 | Phrymaceae |  |
| Plants | 3505 | *Betula pendula* | 1 | 0.152 | 0.004 | Betulaceae |  |
| Plants | 4565 | *Triticum aestivum* | 1 | 0.152 | 0.004 | Poaceae |  |
| Plants | 225215 | *Azorella acaulis* | 1 | 0.152 | 0.004 | Apiaceae |  |
| Fungi | 4896 | *Schizosaccharomyces pombe* | 200 | 7.130 | 0.755 | Schizosaccharomycetaceae |  |
| Fungi | 42258 | *Zygosaccharomyces mellis* | 82 | 2.923 | 0.310 | Saccharomycetaceae |  |
| Fungi | 4956 | *Zygosaccharomyces rouxii* | 59 | 2.103 | 0.223 | Saccharomycetaceae |  |
| Fungi | 1365886 | *Zygosaccharomyces parabailii* | 48 | 1.711 | 0.181 | Saccharomycetaceae |  |
| Fungi | 4950 | *Torulaspora delbrueckii* | 8 | 0.285 | 0.030 | Saccharomycetaceae |  |
| Fungi | 27322 | *Metschnikowia bicuspidata* | 7 | 0.250 | 0.026 | Metschnikowiaceae |  |
| Fungi | 36911 | *Clavispora lusitaniae* | 7 | 0.250 | 0.026 | Metschnikowiaceae |  |
| Fungi | 500148 | *Metarhizium brunneum* | 6 | 0.214 | 0.023 | Clavicipitaceae |  |
| Fungi | 4932 | *Saccharomyces cerevisiae* | 5 | 0.178 | 0.019 | Saccharomycetaceae |  |
| Fungi | 204045 | *Schizosaccharomyces kambucha* | 4 | 0.143 | 0.015 | Schizosaccharomycetaceae |  |
| Fungi | 4927 | *Wickerhamomyces anomalus* | 4 | 0.143 | 0.015 | Phaffomycetaceae |  |
| Fungi | 51657 | *Nakaseomyces delphensis* | 3 | 0.107 | 0.011 | Saccharomycetaceae |  |
| Fungi | 5599 | *Alternaria alternata* | 3 | 0.107 | 0.011 | Pleosporaceae |  |
| Fungi | 1071840 | *Bettsia alvei* | 2 | 0.071 | 0.008 | NO_NAME |  |
| Fungi | 75736 | *Starmerella bombicola* | 2 | 0.071 | 0.008 | NO_NAME |  |
| Fungi | 40302 | *Nosema ceranae* | 2 | 0.071 | 0.008 | Nosematidae |  |
| Fungi | 36033 | *Vanderwaltozyma polyspora* | 2 | 0.071 | 0.008 | Saccharomycetaceae |  |
| Fungi | 381046 | *Lachancea thermotolerans* | 1 | 0.036 | 0.004 | Saccharomycetaceae |  |
| Fungi | 1708541 | *Wallemia mellicola* | 1 | 0.036 | 0.004 | NO_NAME |  |
| Fungi | 1191714 | *Schizosaccharomyces sp. UFLA CHYE5.39* | 1 | 0.036 | 0.004 | Schizosaccharomycetaceae |  |
| Fungi | 384582 | *Zygosaccharomyces sp. G70* | 1 | 0.036 | 0.004 | Saccharomycetaceae |  |
| Fungi | 35629 | *Torulaspora pretoriensis* | 1 | 0.036 | 0.004 | Saccharomycetaceae |  |
| Fungi | 1108849 | *Penicillium rubens* | 1 | 0.036 | 0.004 | Aspergillaceae |  |
| Fungi | 53488 | *Torulaspora franciscae* | 1 | 0.036 | 0.004 | Saccharomycetaceae |  |
| Fungi | 76773 | *Malassezia globosa* | 1 | 0.036 | 0.004 | Malasseziaceae |  |
| Fungi | 45517 | *Metschnikowia gruessii* | 1 | 0.036 | 0.004 | Metschnikowiaceae |  |
| Fungi | 1685470 | *Metschnikowia sp. NJ-2015* | 1 | 0.036 | 0.004 | Metschnikowiaceae |  |
| Fungi | 29833 | *Hanseniaspora uvarum* | 1 | 0.036 | 0.004 | Saccharomycodaceae |  |
| Fungi | 36907 | *Kazachstania telluris* | 1 | 0.036 | 0.004 | Saccharomycetaceae |  |
| Fungi | 353897 | *Metschnikowia aberdeeniae* | 1 | 0.036 | 0.004 | Metschnikowiaceae |  |
| Fungi | 1042127 | *Aureobasidium subglaciale* | 1 | 0.036 | 0.004 | Saccotheciaceae |  |
| Fungi | 104416 | *Filobasidium wieringae* | 1 | 0.036 | 0.004 | Filobasidiaceae |  |
| Fungi | 88768 | *Metschnikowia sp. IFO1406* | 1 | 0.036 | 0.004 | Metschnikowiaceae |  |
| Fungi | 1134516 | *Metschnikowia sp. Y-37* | 1 | 0.036 | 0.004 | Metschnikowiaceae |  |
| Fungi | 1090491 | *Zygosaccharomyces sp. NCAIM Y.01994* | 1 | 0.036 | 0.004 | Saccharomycetaceae |  |
| Fungi | 4934 | *Lachancea kluyveri* | 1 | 0.036 | 0.004 | Saccharomycetaceae |  |
| Fungi | 4957 | *Zygosaccharomyces bisporus* | 1 | 0.036 | 0.004 | Saccharomycetaceae |  |
| Fungi | 1432397 | *Metschnikowia sp. UWOPS 03-147.1* | 1 | 0.036 | 0.004 | Metschnikowiaceae |  |
| Fungi | 1461763 | *Zygosaccharomyces sapae* | 1 | 0.036 | 0.004 | Saccharomycetaceae |  |
| Fungi | 45354 | *[Candida] intermedia* | 1 | 0.036 | 0.004 | Metschnikowiaceae |  |
| Bacteria | 148814 | *Lactobacillus kunkeei* | 21485 | 25.104 | 81.105 | Lactobacillaceae |  |
| Bacteria | 542 | *Zymomonas mobilis* | 11084 | 12.951 | 41.842 | Sphingomonadaceae |  |
| Bacteria | 1396 | *Bacillus cereus* | 759 | 0.887 | 2.865 | Bacillaceae |  |
| Bacteria | 1520 | *Clostridium beijerinckii* | 363 | 0.424 | 1.370 | Clostridiaceae |  |
| Bacteria | 33970 | *Melissococcus plutonius* | 152 | 0.178 | 0.574 | Enterococcaceae |  |
| Bacteria | 442 | *Gluconobacter oxydans* | 148 | 0.173 | 0.559 | Acetobacteraceae |  |
| Bacteria | 1358 | *Lactococcus lactis* | 148 | 0.173 | 0.559 | Streptococcaceae |  |
| Bacteria | 28152 | *Yersinia kristensenii* | 148 | 0.173 | 0.559 | Yersiniaceae |  |
| Bacteria | 545 | *Citrobacter koseri* | 141 | 0.165 | 0.532 | Enterobacteriaceae |  |
| Bacteria | 1510841 | *Parasaccharibacter apium* | 122 | 0.143 | 0.461 | Acetobacteraceae |  |
| Bacteria | 1692238 | *Enterobacter sp. FY-07* | 116 | 0.136 | 0.438 | Enterobacteriaceae |  |
| Bacteria | 446692 | *Acetobacter senegalensis* | 90 | 0.105 | 0.340 | Acetobacteraceae |  |
| Bacteria | 1597 | *Lactobacillus paracasei* | 71 | 0.083 | 0.268 | Lactobacillaceae |  |
| Bacteria | 28901 | *Salmonella enterica* | 70 | 0.082 | 0.264 | Enterobacteriaceae |  |
| Bacteria | 487316 | *Acinetobacter soli* | 70 | 0.082 | 0.264 | Moraxellaceae |  |
| Bacteria | 1076596 | *Acetobacter persici* | 57 | 0.067 | 0.215 | Acetobacteraceae |  |
| Bacteria | 153496 | *Kozakia baliensis* | 54 | 0.063 | 0.204 | Acetobacteraceae |  |
| Bacteria | 552 | *Erwinia amylovora* | 49 | 0.057 | 0.185 | Erwiniaceae |  |
| Bacteria | 215689 | *Erwinia sp. Ejp617* | 48 | 0.056 | 0.181 | Erwiniaceae |  |
| Bacteria | 33995 | *Komagataeibacter europaeus* | 48 | 0.056 | 0.181 | Acetobacteraceae |  |
| Bacteria | 438 | *Acetobacter pasteurianus* | 47 | 0.055 | 0.177 | Acetobacteraceae |  |
| Bacteria | 2133 | *Spiroplasma citri* | 47 | 0.055 | 0.177 | Spiroplasmataceae |  |
| Bacteria | 338565 | *Erwinia tasmaniensis* | 40 | 0.047 | 0.151 | Erwiniaceae |  |
| Bacteria | 1351 | *Enterococcus faecalis* | 40 | 0.047 | 0.151 | Enterococcaceae |  |
| Bacteria | 93218 | *Pandoraea apista* | 37 | 0.043 | 0.140 | Burkholderiaceae |  |
| Bacteria | 66269 | *Pantoea stewartii* | 34 | 0.040 | 0.128 | Erwiniaceae |  |
| Bacteria | 1484157 | *Pantoea sp. PSNIH2* | 33 | 0.039 | 0.125 | Erwiniaceae |  |
| Bacteria | 158836 | *Enterobacter hormaechei* | 32 | 0.037 | 0.121 | Enterobacteriaceae |  |
| Bacteria | 2033 | *Microbacterium testaceum* | 29 | 0.034 | 0.109 | Microbacteriaceae |  |
| Bacteria | 571 | *Klebsiella oxytoca* | 27 | 0.032 | 0.102 | Enterobacteriaceae |  |
| Bacteria | 1428 | *Bacillus thuringiensis* | 27 | 0.032 | 0.102 | Bacillaceae |  |
| Bacteria | 182337 | *Erwinia billingiae* | 27 | 0.032 | 0.102 | Erwiniaceae |  |
| Bacteria | 1166130 | *Enterobacter sp. R4-368* | 27 | 0.032 | 0.102 | Enterobacteriaceae |  |
| Bacteria | 546 | *Citrobacter freundii* | 26 | 0.030 | 0.098 | Enterobacteriaceae |  |
| Bacteria | 431306 | *Acetobacter ghanensis* | 26 | 0.030 | 0.098 | Acetobacteraceae |  |
| Bacteria | 435 | *Acetobacter aceti* | 26 | 0.030 | 0.098 | Acetobacteraceae |  |
| Bacteria | 573 | *Klebsiella pneumoniae* | 26 | 0.030 | 0.098 | Enterobacteriaceae |  |
| Bacteria | 562 | *Escherichia coli* | 26 | 0.030 | 0.098 | Enterobacteriaceae |  |
| Bacteria | 1686310 | *Bartonella apis* | 24 | 0.028 | 0.091 | Bartonellaceae |  |
| Bacteria | 638 | *Arsenophonus nasoniae* | 23 | 0.027 | 0.087 | Morganellaceae |  |
| Bacteria | 592316 | *Pantoea sp. At-9b* | 22 | 0.026 | 0.083 | Erwiniaceae |  |
| Bacteria | 53336 | *Tatumella citrea* | 22 | 0.026 | 0.083 | Erwiniaceae |  |
| Bacteria | 549 | *Pantoea agglomerans* | 21 | 0.025 | 0.079 | Erwiniaceae |  |
| Bacteria | 375175 | *Lactobacillus backii* | 21 | 0.025 | 0.079 | Lactobacillaceae |  |
| Bacteria | 61648 | *Kluyvera intermedia* | 21 | 0.025 | 0.079 | Enterobacteriaceae |  |
| Bacteria | 1906742 | *Microbacterium sp. BH-3-3-3* | 20 | 0.023 | 0.075 | Microbacteriaceae |  |
| Bacteria | 550 | *Enterobacter cloacae* | 19 | 0.022 | 0.072 | Enterobacteriaceae |  |
| Bacteria | 553 | *Pantoea ananatis* | 19 | 0.022 | 0.072 | Erwiniaceae |  |
| Bacteria | 28448 | *Komagataeibacter xylinus* | 18 | 0.021 | 0.068 | Acetobacteraceae |  |
| Bacteria | 1218496 | *Lactobacillus sp. Fhon2N* | 17 | 0.020 | 0.064 | Lactobacillaceae |  |
| Bacteria | 158822 | *Cedecea neteri* | 15 | 0.018 | 0.057 | Enterobacteriaceae |  |
| Bacteria | 91915 | *Asaia bogorensis* | 15 | 0.018 | 0.057 | Acetobacteraceae |  |
| Bacteria | 1196083 | *Snodgrassella alvi* | 15 | 0.018 | 0.057 | Neisseriaceae |  |
| Bacteria | 1545702 | *Lactobacillus sp. wkB8* | 15 | 0.018 | 0.057 | Lactobacillaceae |  |
| Bacteria | 615 | *Serratia marcescens* | 14 | 0.016 | 0.053 | Yersiniaceae |  |
| Bacteria | 891974 | *Plautia stali symbiont* | 14 | 0.016 | 0.053 | Enterobacteriaceae |  |
| Bacteria | 318683 | *Gluconobacter albidus* | 12 | 0.014 | 0.045 | Acetobacteraceae |  |
| Bacteria | 79967 | *Erwinia pyrifoliae* | 12 | 0.014 | 0.045 | Erwiniaceae |  |
| Bacteria | 1633874 | *Acetobacter sp. SLV-7* | 12 | 0.014 | 0.045 | Acetobacteraceae |  |
| Bacteria | 1177712 | *Komagataeibacter medellinensis* | 12 | 0.014 | 0.045 | Acetobacteraceae |  |
| Bacteria | 263819 | *Yersinia aleksiciae* | 12 | 0.014 | 0.045 | Yersiniaceae |  |
| Bacteria | 61645 | *Enterobacter asburiae* | 12 | 0.014 | 0.045 | Enterobacteriaceae |  |
| Bacteria | 1619313 | *Erwinia gerundensis* | 11 | 0.013 | 0.042 | Erwiniaceae |  |
| Bacteria | 215221 | *Komagataeibacter rhaeticus* | 11 | 0.013 | 0.042 | Acetobacteraceae |  |
| Bacteria | 1582 | *Lactobacillus casei* | 11 | 0.013 | 0.042 | Lactobacillaceae |  |
| Bacteria | 82996 | *Serratia plymuthica* | 10 | 0.012 | 0.038 | Yersiniaceae |  |
| Bacteria | 470934 | *Pantoea vagans* | 10 | 0.012 | 0.038 | Erwiniaceae |  |
| Bacteria | 57706 | *Citrobacter braakii* | 10 | 0.012 | 0.038 | Enterobacteriaceae |  |
| Bacteria | 1789224 | *Acinetobacter larvae* | 10 | 0.012 | 0.038 | Moraxellaceae |  |
| Bacteria | 1590 | *Lactobacillus plantarum* | 9 | 0.011 | 0.034 | Lactobacillaceae |  |
| Bacteria | 1267021 | *Frischella perrara* | 9 | 0.011 | 0.034 | Orbaceae |  |
| Bacteria | 106654 | *Acinetobacter nosocomialis* | 9 | 0.011 | 0.034 | Moraxellaceae |  |
| Bacteria | 1544694 | *Rosenbergiella epipactidis* | 8 | 0.009 | 0.030 | Enterobacteriaceae |  |
| Bacteria | 146474 | *Acetobacter orientalis* | 8 | 0.009 | 0.030 | Acetobacteraceae |  |
| Bacteria | 554 | *Pectobacterium carotovorum* | 8 | 0.009 | 0.030 | Pectobacteriaceae |  |
| Bacteria | 633 | *Yersinia pseudotuberculosis* | 8 | 0.009 | 0.030 | Yersiniaceae |  |
| Bacteria | 2137 | *Spiroplasma apis* | 8 | 0.009 | 0.030 | Spiroplasmataceae |  |
| Bacteria | 33996 | *Gluconacetobacter diazotrophicus* | 8 | 0.009 | 0.030 | Acetobacteraceae |  |
| Bacteria | 138074 | *Serratia symbiotica* | 7 | 0.008 | 0.026 | Yersiniaceae |  |
| Bacteria | 40215 | *Acinetobacter junii* | 7 | 0.008 | 0.026 | Moraxellaceae |  |
| Bacteria | 1196095 | *Gilliamella apicola* | 7 | 0.008 | 0.026 | Orbaceae |  |
| Bacteria | 563 | *Shimwellia blattae* | 7 | 0.008 | 0.026 | Enterobacteriaceae |  |
| Bacteria | 470 | *Acinetobacter baumannii* | 7 | 0.008 | 0.026 | Moraxellaceae |  |
| Bacteria | 1484158 | *Pantoea sp. PSNIH1* | 6 | 0.007 | 0.023 | Erwiniaceae |  |
| Bacteria | 1891675 | *Pantoea alhagi* | 6 | 0.007 | 0.023 | Erwiniaceae |  |
| Bacteria | 59814 | *Pantoea dispersa* | 6 | 0.007 | 0.023 | Erwiniaceae |  |
| Bacteria | 497725 | *Kosakonia oryzae* | 6 | 0.007 | 0.023 | Enterobacteriaceae |  |
| Bacteria | 1464 | *Paenibacillus larvae* | 5 | 0.006 | 0.019 | Paenibacillaceae |  |
| Bacteria | 187493 | *Thalassolituus oleivorans* | 5 | 0.006 | 0.019 | Oceanospirillaceae |  |
| Bacteria | 632 | *Yersinia pestis* | 4 | 0.005 | 0.015 | Yersiniaceae |  |
| Bacteria | 1892404 | *Bacillus sp. ABP14* | 4 | 0.005 | 0.015 | Bacillaceae |  |
| Bacteria | 561879 | *Bacillus safensis* | 4 | 0.005 | 0.015 | Bacillaceae |  |
| Bacteria | 1219383 | *Acinetobacter boissieri* | 4 | 0.005 | 0.015 | Moraxellaceae |  |
| Bacteria | 1158459 | *Kosakonia sacchari* | 4 | 0.005 | 0.015 | Enterobacteriaceae |  |
| Bacteria | 35703 | *Citrobacter amalonaticus* | 3 | 0.004 | 0.011 | Enterobacteriaceae |  |
| Bacteria | 36873 | *Paraburkholderia xenovorans* | 3 | 0.004 | 0.011 | Burkholderiaceae |  |
| Bacteria | 1173099 | *bacterium endosymbiont of Rhamphus pulicarius* | 3 | 0.004 | 0.011 | Enterobacteriaceae |  |
| Bacteria | 147645 | *Paracoccus yeei* | 3 | 0.004 | 0.011 | Rhodobacteraceae |  |
| Bacteria | 47715 | *Lactobacillus rhamnosus* | 3 | 0.004 | 0.011 | Lactobacillaceae |  |
| Bacteria | 584 | *Proteus mirabilis* | 3 | 0.004 | 0.011 | Morganellaceae |  |
| Bacteria | 84292 | *Microbacterium chocolatum* | 3 | 0.004 | 0.011 | Microbacteriaceae |  |
| Bacteria | 40324 | *Stenotrophomonas maltophilia* | 3 | 0.004 | 0.011 | Xanthomonadaceae |  |
| Bacteria | 28090 | *Acinetobacter lwoffii* | 3 | 0.004 | 0.011 | Moraxellaceae |  |
| Bacteria | 330276 | *Bacillus sp. Am 08* | 3 | 0.004 | 0.011 | Bacillaceae |  |
| Bacteria | 157463 | *Fructobacillus ficulneus* | 3 | 0.004 | 0.011 | Leuconostocaceae |  |
| Bacteria | 62977 | *Acinetobacter sp. ADP1* | 3 | 0.004 | 0.011 | Moraxellaceae |  |
| Bacteria | 1697053 | *Oblitimonas alkaliphila* | 3 | 0.004 | 0.011 | Pseudomonadaceae |  |
| Bacteria | 1245 | *Leuconostoc mesenteroides* | 3 | 0.004 | 0.011 | Leuconostocaceae |  |
| Bacteria | 108981 | *Acinetobacter schindleri* | 3 | 0.004 | 0.011 | Moraxellaceae |  |
| Bacteria | 1915310 | *Enterobacter cloacae complex sp. ECNIH7* | 3 | 0.004 | 0.011 | Enterobacteriaceae |  |
| Bacteria | 126162 | *secondary endosymbiont of Glycaspis brimblecombei* | 3 | 0.004 | 0.011 | Enterobacteriaceae |  |
| Bacteria | 29471 | *Pectobacterium atrosepticum* | 3 | 0.004 | 0.011 | Pectobacteriaceae |  |
| Bacteria | 1684 | *Bifidobacterium asteroides* | 3 | 0.004 | 0.011 | Bifidobacteriaceae |  |
| Bacteria | 61652 | *Serratia rubidaea* | 2 | 0.002 | 0.008 | Yersiniaceae |  |
| Bacteria | 208223 | *Kosakonia cowanii* | 2 | 0.002 | 0.008 | Enterobacteriaceae |  |
| Bacteria | 54291 | *Raoultella ornithinolytica* | 2 | 0.002 | 0.008 | Enterobacteriaceae |  |
| Bacteria | 1933220 | *Sphingobacterium sp. B29* | 2 | 0.002 | 0.008 | Sphingobacteriaceae |  |
| Bacteria | 1492 | *Clostridium butyricum* | 2 | 0.002 | 0.008 | Clostridiaceae |  |
| Bacteria | 300019 | *Microbacterium paludicola* | 2 | 0.002 | 0.008 | Microbacteriaceae |  |
| Bacteria | 294 | *Pseudomonas fluorescens* | 2 | 0.002 | 0.008 | Pseudomonadaceae |  |
| Bacteria | 317 | *Pseudomonas syringae* | 2 | 0.002 | 0.008 | Pseudomonadaceae |  |
| Bacteria | 703 | *Plesiomonas shigelloides* | 2 | 0.002 | 0.008 | NO_NAME |  |
| Bacteria | 67780 | *Edwardsiella ictaluri* | 2 | 0.002 | 0.008 | Hafniaceae |  |
| Bacteria | 628 | *Xenorhabdus nematophila* | 2 | 0.002 | 0.008 | Morganellaceae |  |
| Bacteria | 1194345 | *Candidatus Snodgrassella sp. TA1_30860* | 2 | 0.002 | 0.008 | Neisseriaceae |  |
| Bacteria | 1402 | *Bacillus licheniformis* | 2 | 0.002 | 0.008 | Bacillaceae |  |
| Bacteria | 709323 | *Fructobacillus tropaeoli* | 2 | 0.002 | 0.008 | Leuconostocaceae |  |
| Bacteria | 1363 | *Lactococcus garvieae* | 2 | 0.002 | 0.008 | Streptococcaceae |  |
| Bacteria | 42862 | *Rickettsia felis* | 2 | 0.002 | 0.008 | Rickettsiaceae |  |
| Bacteria | 630 | *Yersinia enterocolitica* | 2 | 0.002 | 0.008 | Yersiniaceae |  |
| Bacteria | 1604 | *Lactobacillus amylovorus* | 2 | 0.002 | 0.008 | Lactobacillaceae |  |
| Bacteria | 152331 | *Lactobacillus parabuchneri* | 2 | 0.002 | 0.008 | Lactobacillaceae |  |
| Bacteria | 1407071 | *Acinetobacter sp. TGL-Y2* | 2 | 0.002 | 0.008 | Moraxellaceae |  |
| Bacteria | 988801 | *Rosenbergiella nectarea* | 2 | 0.002 | 0.008 | Enterobacteriaceae |  |
| Bacteria | 67827 | *Citrobacter werkmanii* | 2 | 0.002 | 0.008 | Enterobacteriaceae |  |
| Bacteria | 246432 | *Staphylococcus equorum* | 2 | 0.002 | 0.008 | Staphylococcaceae |  |
| Bacteria | 29486 | *Yersinia ruckeri* | 2 | 0.002 | 0.008 | Yersiniaceae |  |
| Bacteria | 81475 | *Frateuria aurantia* | 2 | 0.002 | 0.008 | Rhodanobacteraceae |  |
| Bacteria | 1076550 | *Pantoea rwandensis* | 2 | 0.002 | 0.008 | Erwiniaceae |  |
| Bacteria | 1608473 | *Acinetobacter sp. NCu2D-2* | 2 | 0.002 | 0.008 | Moraxellaceae |  |
| Bacteria | 1218495 | *Lactobacillus apinorum* | 2 | 0.002 | 0.008 | Lactobacillaceae |  |
| Bacteria | 82688 | *Lactobacillus nagelii* | 2 | 0.002 | 0.008 | Lactobacillaceae |  |
| Bacteria | 265960 | *Komagataeibacter nataicola* | 2 | 0.002 | 0.008 | Acetobacteraceae |  |
| Bacteria | 988805 | *Enterobacteriaceae bacterium 8NS7* | 2 | 0.002 | 0.008 | Enterobacteriaceae |  |
| Bacteria | 712633 | *Streptococcus sp. oral taxon 431* | 1 | 0.001 | 0.004 | Streptococcaceae |  |
| Bacteria | 33934 | *Anoxybacillus flavithermus* | 1 | 0.001 | 0.004 | Bacillaceae |  |
| Bacteria | 1712675 | *Turicibacter sp. H121* | 1 | 0.001 | 0.004 | Erysipelotrichaceae |  |
| Bacteria | 240427 | *Lactobacillus paracollinoides* | 1 | 0.001 | 0.004 | Lactobacillaceae |  |
| Bacteria | 178901 | *Acetobacter malorum* | 1 | 0.001 | 0.004 | Acetobacteraceae |  |
| Bacteria | 988707 | *Acinetobacter sp. 12N1* | 1 | 0.001 | 0.004 | Moraxellaceae |  |
| Bacteria | 48296 | *Acinetobacter pittii* | 1 | 0.001 | 0.004 | Moraxellaceae |  |
| Bacteria | 290111 | *Xenorhabdus ehlersii* | 1 | 0.001 | 0.004 | Morganellaceae |  |
| Bacteria | 69224 | *Erwinia psidii* | 1 | 0.001 | 0.004 | Erwiniaceae |  |
| Bacteria | 43675 | *Rothia mucilaginosa* | 1 | 0.001 | 0.004 | Micrococcaceae |  |
| Bacteria | 1408 | *Bacillus pumilus* | 1 | 0.001 | 0.004 | Bacillaceae |  |
| Bacteria | 988718 | *Acinetobacter sp. 18N3* | 1 | 0.001 | 0.004 | Moraxellaceae |  |
| Bacteria | 548 | *Klebsiella aerogenes* | 1 | 0.001 | 0.004 | Enterobacteriaceae |  |
| Bacteria | 285 | *Comamonas testosteroni* | 1 | 0.001 | 0.004 | Comamonadaceae |  |
| Bacteria | 36805 | *Microbacterium aurum* | 1 | 0.001 | 0.004 | Microbacteriaceae |  |
| Bacteria | 28108 | *Alteromonas macleodii* | 1 | 0.001 | 0.004 | Alteromonadaceae |  |
| Bacteria | 255519 | *Atlantibacter subterranea* | 1 | 0.001 | 0.004 | Enterobacteriaceae |  |
| Bacteria | 220990 | *Asaia krungthepensis* | 1 | 0.001 | 0.004 | Acetobacteraceae |  |
| Bacteria | 1746743 | *Microbacterium sp. HBUM178421* | 1 | 0.001 | 0.004 | Microbacteriaceae |  |
| Bacteria | 693444 | *Enterobacteriaceae bacterium strain FGI 57* | 1 | 0.001 | 0.004 | Enterobacteriaceae |  |
| Bacteria | 299767 | *Enterobacter ludwigii* | 1 | 0.001 | 0.004 | Enterobacteriaceae |  |
| Bacteria | 257708 | *Roseomonas gilardii* | 1 | 0.001 | 0.004 | Acetobacteraceae |  |
| Bacteria | 38289 | *Corynebacterium jeikeium* | 1 | 0.001 | 0.004 | Corynebacteriaceae |  |
| Bacteria | 528191 | *Paenibacillus xylanexedens* | 1 | 0.001 | 0.004 | Paenibacillaceae |  |
| Bacteria | 34062 | *Moraxella osloensis* | 1 | 0.001 | 0.004 | Moraxellaceae |  |
| Bacteria | 657310 | *Enterococcus sp. 7L76* | 1 | 0.001 | 0.004 | Enterococcaceae |  |
| Bacteria | 1905288 | *Klebsiella sp. LTGPAF-6F* | 1 | 0.001 | 0.004 | Enterobacteriaceae |  |
| Bacteria | 1463165 | *Klebsiella quasipneumoniae* | 1 | 0.001 | 0.004 | Enterobacteriaceae |  |
| Bacteria | 357276 | *Bacteroides dorei* | 1 | 0.001 | 0.004 | Bacteroidaceae |  |
| Bacteria | 43771 | *Corynebacterium urealyticum* | 1 | 0.001 | 0.004 | Corynebacteriaceae |  |
| Bacteria | 1390395 | *Sphingomonas sp. LK11* | 1 | 0.001 | 0.004 | Sphingomonadaceae |  |
| Bacteria | 95485 | *Burkholderia stabilis* | 1 | 0.001 | 0.004 | Burkholderiaceae |  |
| Bacteria | 1134687 | *Klebsiella michiganensis* | 1 | 0.001 | 0.004 | Enterobacteriaceae |  |
| Bacteria | 1809055 | *Acinetobacter sp. DUT-2* | 1 | 0.001 | 0.004 | Moraxellaceae |  |
| Bacteria | 83655 | *Leclercia adecarboxylata* | 1 | 0.001 | 0.004 | Enterobacteriaceae |  |
| Bacteria | 1327989 | *Serratia sp. FS14* | 1 | 0.001 | 0.004 | Yersiniaceae |  |
| Bacteria | 40214 | *Acinetobacter johnsonii* | 1 | 0.001 | 0.004 | Moraxellaceae |  |
| Bacteria | 488142 | *Serratia sp. SCBI* | 1 | 0.001 | 0.004 | Yersiniaceae |  |
| Bacteria | 588 | *Providencia stuartii* | 1 | 0.001 | 0.004 | Morganellaceae |  |
| Bacteria | 1978566 | *Cryobacterium sp. LW097* | 1 | 0.001 | 0.004 | Microbacteriaceae |  |
| Bacteria | 92932 | *Pantoea cedenensis* | 1 | 0.001 | 0.004 | Erwiniaceae |  |
| Bacteria | 70863 | *Shewanella oneidensis* | 1 | 0.001 | 0.004 | Shewanellaceae |  |
| Bacteria | 259059 | *Lactobacillus satsumensis* | 1 | 0.001 | 0.004 | Lactobacillaceae |  |
| Bacteria | 1747 | *Cutibacterium acnes* | 1 | 0.001 | 0.004 | Propionibacteriaceae |  |
| Bacteria | 33069 | *Pseudomonas viridiflava* | 1 | 0.001 | 0.004 | Pseudomonadaceae |  |
| Bacteria | 644 | *Aeromonas hydrophila* | 1 | 0.001 | 0.004 | Aeromonadaceae |  |
| Bacteria | 469595 | *Citrobacter sp. 30_2* | 1 | 0.001 | 0.004 | Enterobacteriaceae |  |
| Bacteria | 91624 | *Clostridium acidisoli* | 1 | 0.001 | 0.004 | Clostridiaceae |  |
| Bacteria | 339 | *Xanthomonas campestris* | 1 | 0.001 | 0.004 | Xanthomonadaceae |  |
| Bacteria | 1560339 | *Enterobacter sp. E20* | 1 | 0.001 | 0.004 | Enterobacteriaceae |  |
| Bacteria | 67825 | *Citrobacter rodentium* | 1 | 0.001 | 0.004 | Enterobacteriaceae |  |
| Bacteria | 198620 | *Pseudomonas koreensis* | 1 | 0.001 | 0.004 | Pseudomonadaceae |  |
| Bacteria | 33074 | *Zymobacter palmae* | 1 | 0.001 | 0.004 | Halomonadaceae |  |
| Bacteria | 1138822 | *Lactobacillus curieae* | 1 | 0.001 | 0.004 | Lactobacillaceae |  |
| Bacteria | 1816219 | *Colwellia sp. PAMC 21821* | 1 | 0.001 | 0.004 | Colwelliaceae |  |
| Bacteria | 89059 | *Lactobacillus acidipiscis* | 1 | 0.001 | 0.004 | Lactobacillaceae |  |
| Bacteria | 28141 | *Cronobacter sakazakii* | 1 | 0.001 | 0.004 | Enterobacteriaceae |  |
| Bacteria | 232721 | *Acidovorax sp. JS42* | 1 | 0.001 | 0.004 | Comamonadaceae |  |
| Bacteria | 1541173 | *bacterium NJ6* | 1 | 0.001 | 0.004 | NO_NAME |  |
| Bacteria | 80878 | *Acidovorax temperans* | 1 | 0.001 | 0.004 | Comamonadaceae |  |
| Bacteria | 61647 | *Pluralibacter gergoviae* | 1 | 0.001 | 0.004 | Enterobacteriaceae |  |
| Bacteria | 244366 | *Klebsiella variicola* | 1 | 0.001 | 0.004 | Enterobacteriaceae |  |
| Bacteria | 38308 | *Gluconobacter frateurii* | 1 | 0.001 | 0.004 | Acetobacteraceae |  |
| Bacteria | 47877 | *Pseudomonas amygdali* | 1 | 0.001 | 0.004 | Pseudomonadaceae |  |
| Bacteria | 1827481 | *Enterobacter sp. ODB01* | 1 | 0.001 | 0.004 | Enterobacteriaceae |  |
| Bacteria | 85698 | *Achromobacter xylosoxidans* | 1 | 0.001 | 0.004 | Alcaligenaceae |  |
| Bacteria | 413502 | *Cronobacter turicensis* | 1 | 0.001 | 0.004 | Enterobacteriaceae |  |
| Bacteria | 1644893 | *Enterobacter sp. UIWRF1401* | 1 | 0.001 | 0.004 | Enterobacteriaceae |  |
| Bacteria | 569 | *Hafnia alvei* | 1 | 0.001 | 0.004 | Hafniaceae |  |
| Bacteria | 867805 | *bacterium P2-10-1* | 1 | 0.001 | 0.004 | NO_NAME |  |
| Bacteria | 216142 | *Pseudomonas rhizosphaerae* | 1 | 0.001 | 0.004 | Pseudomonadaceae |  |
| Bacteria | 2134 | *Spiroplasma melliferum* | 1 | 0.001 | 0.004 | Spiroplasmataceae |  |
| Bacteria | 207513 | *symbiont of Wahlgreniella nervata* | 1 | 0.001 | 0.004 | Enterobacteriaceae |  |
| Bacteria | 172042 | *Rothia aeria* | 1 | 0.001 | 0.004 | Micrococcaceae |  |
| Bacteria | 104100 | *Acetobacter lovaniensis* | 1 | 0.001 | 0.004 | Acetobacteraceae |  |
| Bacteria | 1296 | *Staphylococcus sciuri* | 1 | 0.001 | 0.004 | Staphylococcaceae |  |
| Bacteria | 1244 | *Leuconostoc gelidum* | 1 | 0.001 | 0.004 | Leuconostocaceae |  |
| Bacteria | 55601 | *Vibrio anguillarum* | 1 | 0.001 | 0.004 | Vibrionaceae |  |
| Bacteria | 988795 | *Enterobacteriaceae bacterium 4NS5* | 1 | 0.001 | 0.004 | Enterobacteriaceae |  |
| Bacteria | 1282 | *Staphylococcus epidermidis* | 1 | 0.001 | 0.004 | Staphylococcaceae |  |
| Bacteria | 1288 | *Staphylococcus xylosus* | 1 | 0.001 | 0.004 | Staphylococcaceae |  |
| Bacteria | 29430 | *Acinetobacter haemolyticus* | 1 | 0.001 | 0.004 | Moraxellaceae |  |
| Bacteria | 988804 | *Enterobacteriaceae bacterium 8NS6* | 1 | 0.001 | 0.004 | Enterobacteriaceae |  |
| Bacteria | 47917 | *Serratia fonticola* | 1 | 0.001 | 0.004 | Yersiniaceae |  |
| Bacteria | 136609 | *Leuconostoc kimchii* | 1 | 0.001 | 0.004 | Leuconostocaceae |  |
| Virus | 1100043 | *Apis mellifera filamentous virus* | 99521 | 57.772 | 375.688 | NO_NAME |  |
| Virus | 1285595 | *Musca hytrovirus* | 14 | 2.134 | 0.053 | Hytrosaviridae |  |
| Virus | 632112 | *Lactobacillus virus Lb338-1* | 4 | 0.610 | 0.015 | Myoviridae |  |
| Virus | 925984 | *Erwinia phage phiEt88* | 1 | 0.152 | 0.004 | Myoviridae |  |
